# Skeletal Morphogenesis of *Microbrachis* and *Hyloplesion* (Tetrapoda: Lepospondyli), and Implications for the Developmental Patterns of Extinct, Early Tetrapods

**DOI:** 10.1371/journal.pone.0128333

**Published:** 2015-06-17

**Authors:** Jennifer C. Olori

**Affiliations:** Department of Geological Sciences, Jackson School of Geosciences, The University of Texas at Austin, Austin, Texas, United States of America; College of the Holy Cross, UNITED STATES

## Abstract

The ontogeny of extant amphibians often is used as a model for that of extinct early tetrapods, despite evidence for a spectrum of developmental modes in temnospondyls and a paucity of ontogenetic data for lepospondyls. I describe the skeletal morphogenesis of the extinct lepospondyls *Microbrachis pelikani* and *Hyloplesion longicostatum* using the largest samples examined for either taxon. Nearly all known specimens were re-examined, allowing for substantial anatomical revisions that affect the scoring of characters commonly used in phylogenetic analyses of early tetrapods. The palate of *H*. *longicostatum* is re-interpreted and suggested to be more similar to that of *M*. *pelikani*, especially in the nature of the contact between the pterygoids. Both taxa possess lateral lines, and *M*. *pelikani* additionally exhibits branchial plates. However, early and rapid ossification of the postcranial skeleton, including a well-developed pubis and ossified epipodials, suggests that neither taxon metamorphosed nor were they neotenic in the sense of branchiosaurids and salamanders. Morphogenetic patterns in the foot suggest that digit 5 was developmentally delayed and the final digit to ossify in *M*. *pelikani* and *H*. *longicostatum*. Overall patterns of postcranial ossification may indicate postaxial dominance in limb and digit formation, but also more developmental variation in early tetrapods than has been appreciated. The phylogenetic position and developmental patterns of *M*. *pelikani* and *H*. *longicostatum* are congruent with the hypothesis that early tetrapods lacked metamorphosis ancestrally and that stem-amniotes exhibited derived features of development, such as rapid and complete ossification of the skeleton, potentially prior to the evolution of the amniotic egg.

## Introduction

The morphologically diverse assemblage of extinct Paleozoic tetrapods traditionally known as ‘microsaurs’ remains controversial with regard to their relationships to both other extinct and living tetrapods. ‘Microsaurs’ were relatively small-bodied, terrestrial animals although many were aquatic and others appear to have been limb-reduced burrowers [1]. The small size of these animals, the presumed holospondylous nature of their vertebrae, and the prevalence of limb reduction were features used historically to ally ‘microsaurs’, nectridians, äistopods, and lysorophids, within the larger group Lepospondyli. Recent large-scale phylogenetic analyses have demonstrated that lepospondyls form a natural assemblage, although the monophyly of ‘microsaurs’ is no longer supported [2–9]. In some phylogenetic hypotheses ‘microsaur’ taxa form successive outgroups leading up to more derived lepospondyls [2,6] whereas in others ‘microsaurs’ are paraphyletic with respect to either all living amphibians (e.g., [9]) or just caecilians [3,4]. A relationship with caecilians is particularly controversial because of many shared anatomical features with basal amniotes, and the fact that modern analyses invariably recover Lepospondyli as more closely related to amniotes than to temnospondyls (including frogs and salamanders), whether or not lepospondyls include caecilians [3–6,8].

Incorporation of ontogenetic data into the reconstruction and critical evaluation of phylogenetic hypotheses, especially with regard to early tetrapod characters, relationships, and the origin of amphibians, is becoming more common (e.g., [3,8,10]). However, the utility of developmental data is limited by lack of ontogenetic information for many Paleozoic taxa. Despite a flurry of interest in early tetrapod ontogeny, especially that of temnospondyls, only a few authors investigated the skeletal development of lepospondyls [11–19]. Lepospondyls frequently are overlooked as an appreciable source of ontogenetic information because it is assumed that like amniotes, they are direct developers with no metamorphosis [20] and cannot inform the evolution of development in Tetrapoda. In particular, little has been done to overhaul traditional views of lepospondyl biology or to reassess the group within a modern ‘evo-devo’ framework. Considering that recently there has been a transformation in perspectives on the distribution of metamorphosis among early tetrapods, a detailed assessment of the developmental patterns in lepospondyls is necessary. Rather than a primitive feature associated with an ontogenetic habitat shift from an aquatic to a terrestrial lifestyle, metamorphosis, defined as a concentration of developmental events [21] often including whole-sale reorganization of the body and skeletal remodeling [22,23], appears to be a derived feature of development present only in extant amphibians and perhaps their closest relatives [24–27].

The shift in perspectives on metamorphosis also has lead to a reassessment of related, heterochronic processes in the evolutionary history of tetrapods, such as neoteny [26,28]. Neoteny is a shift in developmental timing that results in a paedomorphic morphology, paedomorphosis being the retention of juvenile or larval features of an ancestor in the adult of the descendent (i.e, ontogenetic truncation, [29]). The term neoteny originally was used to describe species-level patterns in salamanders in which development was truncated prior to metamorphosis [30]. In this original sense, the neoteny described in salamanders was similar to definitions of paedomorphosis. To clear up confusion between the terms, neoteny was restricted to mean a delay in somatic development, relative to reproductive development, that results in paedomorphic features in sexually mature individuals [31]. Later, the meaning of neoteny was further changed to describe a process that results in paedomorphosis as a result of a decrease in the developmental rate of a feature [29,32]. However, more recently it was recognized that defining neoteny as a decrease in developmental rate was inappropriate because it failed to distinguish reproductive from somatic heterochrony, and lacked the separate recognition of interspecific and intraspecific heterochrony [33]. The latter distinction is important, because although paedomorphosis always refers to a phylogenetic pattern, whereby adult descendents resemble an earlier ontogenetic stage of the ancestor, a single species can have both neotenic and metamorphic populations of individuals, and in fact, some salamanders are facultatively neotenic [34,35]. As a result, the process of slowing the developmental rate as identified by Alberch et al. [32] and Kluge [29], is called *deceleration* [33]. Neoteny is applied to taxa in which sexual maturity occurs during a somatically larval stage of development, but the ancestral condition included metamorphosis (e.g., [28]).

Because neoteny is related to development at the cellular level (i.e., somatic vs. reproductive), this begs the question of whether it can be recognized in the fossil record. In fossils, only skeletal morphology usually is preserved, and there is little indication of absolute ontogenetic age. However, some of the consequences of neoteny, namely paedomorphic features, can be identified when skeletal morphology is affected. In fossils of early tetrapods such features were considered to include ossified branchial plates, a larger number of ceratobranchial bones, bar-like ceratohyals, lateral line canals, hypertrophy of larval skull ornamentation, elaboration of larval teeth, unossified epipodials, and generally weakly developed postcranial elements [24,26,36,37]. However, skeletal evidence for paedomorphosis alone cannot inform whether particular paedomorphic patterns are a result of neoteny or other heterochronic processes.

As in the particular case of extant salamanders, if neoteny is viewed as an alternative to the pathway of metamorphosis [26–28], then it may be possible to predict neoteny in extinct taxa when there is evidence for metamorphosis in some ‘populations’, and additional evidence that other ‘populations’ or closely related taxa with paedomorphic skeletal features, failed to metamorphose. Evidence for alternative developmental pathways of neoteny and metamorphosis were documented in only one group of early tetrapods, the branchiosaurid temnospondyls (*Apateon*, [27,38]). Among lepospondyls, *Microbrachis pelikani* is the only taxon that historically was suggested to have neotenic features [1,3]. Principal evidence for neoteny in *M*. *pelikani* comes from the presence of ossifications associated with external gills. Evidence of soft structures of gills, preserved in branchiosaurids [39], was never observed in specimens of *M*. *pelikani* [15]. Bony branchial plates (‘ceratobranchial dental ossicles’ of [15]) however, were reported [1], although that interpretation was apparently retracted later [9,40]. Additionally, *M*. *pelikani* is one of only two ‘microsaurs’ reported to possess lateral lines (the other is *Saxonerpeton*). Retention of lateral lines, even in the largest and presumably most mature specimens of *M*. *pelikani*, also was used to argue for neoteny in this taxon [1, 3].

Lack of knowledge about the growth and development of lepospondyls is detrimental to the advancement of early tetrapod research because developmental data have important implications for functional studies, maturity assessment, and the characters used for phylogenetic analysis. As an example, it was demonstrated that much of the morphological variation observed in temnospondyls and used to characterize taxa may be intraspecific (i.e., ontogenetic) rather than interspecific [36], and failure to distinguish ontogenetic and phylogenetic variation in phylogenetic analyses leads to unstable hypotheses of relationships [41]. As the level of morphogenesis (i.e., development of the structures and surfaces of bones) increases, features of bones that were absent at earlier stages of development may appear in more mature individuals, which has consequences for character scoring [36]. On the other hand, recognition of ontogenetic variation can lead to the identification of new phylogenetically informative ontogenetic characters that can subsequently be used in broadly comparative studies of relationships [3].

In this paper, I provide descriptions of the skeletal morphogenesis of both *Microbrachis pelikani* and *Hyloplesion longicostatum*. The growth and ontogeny of those taxa is compared to that of extinct temnospondyls and basal amniotes, as well as modern tetrapods. Reconstruction and comparison of postcranial ossification sequences for *M*. *pelikani* and *H*. *longicostatum* were provided in an earlier study [16] as was an investigation of allometric growth in those two taxa [17]. Finally, because almost all known specimens of *M*. *pelikani* (100 individuals) and *H*. *longicostatum* (18 individuals) were examined, I was able to revise and clarify previously ambiguous or unknown skeletal morphologies in these two lepospondyls.

## Materials and Methods

I included a total of 100 specimens of *M*. *pelikani* and 18 specimens of *H*. *longicostatum* in my study. No permits were required for the described study, which complied with all relevant regulations. All specimens were from pre-existing museum collections. The distribution of specimens by skull length and all specimen numbers were published previously [16,17] and also are provided in S1 and S2 Tables. All known specimens of *M*. *pelikani* and all but two specimens of *H*. *longicostatum* come from the Upper Carboniferous ‘Gaskohle’ of the Humboldt Mine, Nýřany, Czech Republic [42]. A small number of *H*. *longicostatum* also were collected from the nearby Třemošná locality, another coal deposit [1]. The Třemošná specimens were not included in the present study because I chose to restrict the investigation to a single geographic population accumulated over a relatively short period of geologic time. The fossil bed at Nýřany, referred to as the ‘Plattelkohle,’ is a 30 cm thick Lagerstätte of finely laminated coal-shales. Previous stratigraphic work based on examination of Recent varvitic coal swamps [43] suggested that the Plattelkohle was deposited gradually over a time-span as short as 300–700 years [44]. The depositional setting is inferred to be a relatively stagnant, anoxic lake or swamp with no benthic fauna [44]. As a result, the quality of preservation frequently is high, with part and counterpart specimens commonly preserved as articulated or associated skeletons. Even in places where the original bone was dissolved through either natural processes or for artificial casting, the remaining natural molds preserve fine details, such as dermal sculpture, scale ornamentation, tiny foramina, and delicate processes.

### Morphogenesis

Cranial and postcranial elements of *H*. *longicostatum* and *M*. *pelikani* were assessed for previously overlooked or ambiguous morphological features in addition to qualitative morphogenetic changes that occur during development. The development of processes, foramina, sculpture, and other osteological features are described for each bone. Developmental changes in morphology occasionally are related to skull size only to provide a relative indication of their comparative timing of appearance. Chronological data are not meant to be treated as formal ontogenetic stages, only as a metric for comparison in the absence of individual age data. Therefore ‘stage’ is used to describe the developmental sequence of one particular element only and cannot be compared across elements (i.e., stage 1 of radius is not equivalent to stage 1 of femur).

Anatomical terms follow those of Carroll and Gaskill [1] and Pawley [36]. Although *Microbrachis* was described previously (e.g., [1,9,15,45,46]), my study includes the largest number of specimens examined for this taxon. Descriptions of select elements for both *M*. *pelikani* and *H*. *longicostatum* focus on ontogenetic changes, variation, and new anatomical information. In some cases, the anatomical re-descriptions and identification of ontogenetically variable features require revision of previously published character codings for *M*. *pelikani* and *H*. *longicostatum* (e.g., [3,4,6,9,47]).

### Phylogenetics

In order to assess the consequences of scoring modifications, I re-ran the most comprehensive phylogenetic analyses available for early tetrapods that also included a broad sampling of lepospondyls [5,6]. The analysis of Huttenlocker et al. [5] was based directly on Anderson et al. [4] with some corrections and additions of characters and lepospondyl taxa. The majority of the characters utilized by Anderson [3] and Vallin and Laurin [9] already were incorporated into the analysis presented by Anderson et al. [4], whereas those of Zanon [47] were redundant with multiple subsequent analyses. Note that Sigurdson and Green [8] made extensive (and mostly justifiable) revisions to the scoring of both Ruta and Coates [6] and Anderson et al. [4] in their supermatrix analysis of tetrapod relationships, including the exclusion of many characters. Because my objective was not to reconstruct tetrapod relationships, but to only to draw attention to and evaluate relatively few scoring modifications for specific lepospondyls, I chose to use the original matrices of Huttenlocker et al. [5] (equals that of [4]) and Ruta and Coates [6] for consistent comparison.

The matrix of Ruta and Coates [6] was re-evaluated using 1000 replicates in TNT, Version 1.1 [48], while that of Huttenlocker et al. [5] was reassessed using multiple, 100 replicate runs. The tree collapsing rule in TNT was set to ‘min. length = 0’ for both analyses. All other parameters followed those provided in the original publications, which utilized PAUP* [49,50].

## Results

### *Microbrachis pelikani*

#### Dermal Ossifications, Sculpture, Lateral Lines, and Branchial Plates

**Fig 1:**
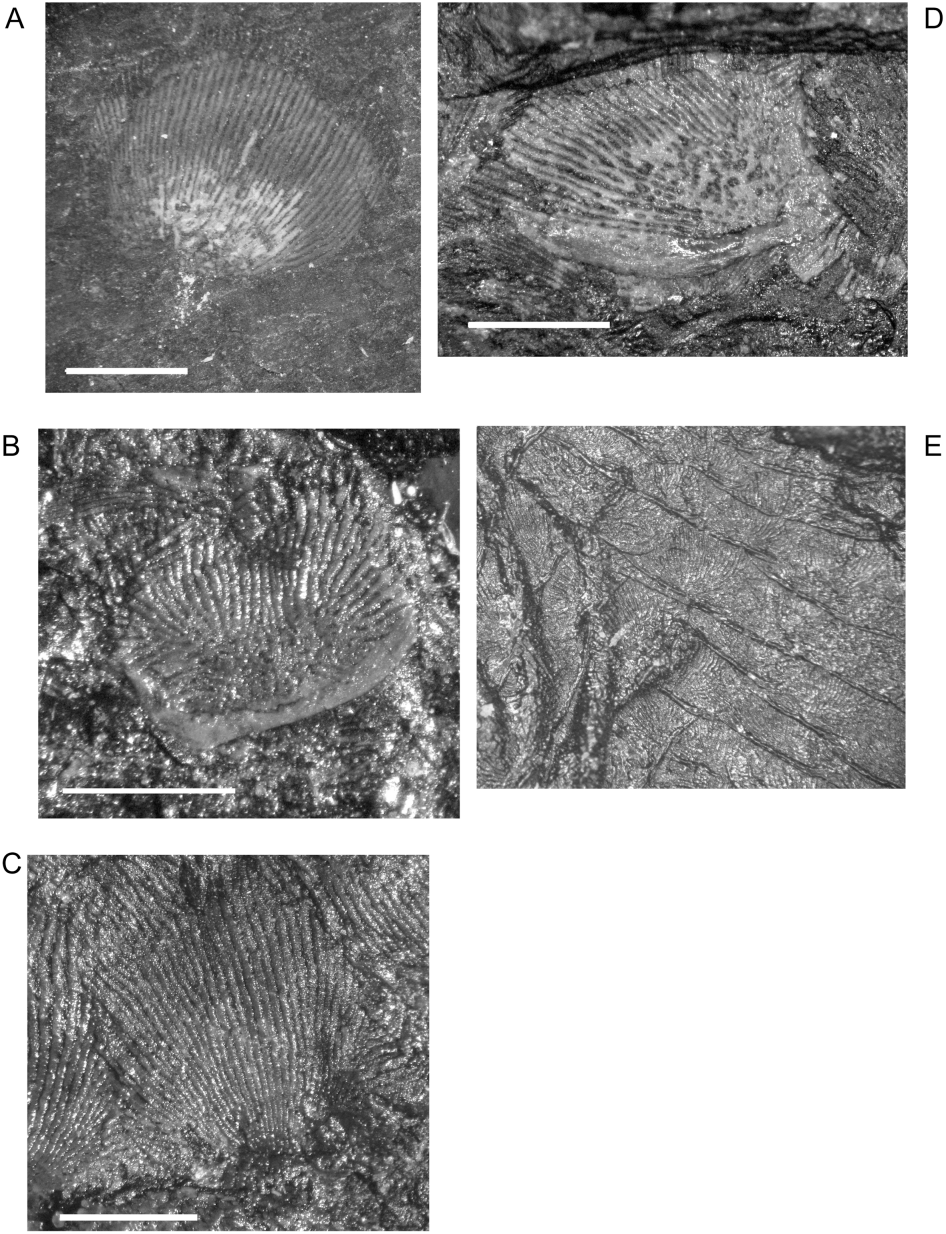
Dermal scales of *M*. *pelikani*. Individual scales (A-D) and in rows (E). Note heavy ridge in B and D. Scale bars = 1mm.

‘Dermal scales’ of ‘microsaurian’ lepospondyls frequently were described in the literature as oat- or rod-shaped elements with a radiating pattern of ornamentation [1,47]. The scales are composed of bone (Fig 1A) and thus ‘dermal scales’ or ‘dermal squamation’ are common terminology for similar features in temnospondyls and other early tetrapods (e.g., [51,52]). Both dorsal and ventral scales were noted previously, and can be distinguished morphologically [15,46]. I also observed two types of dermal ossifications, although association with the ventral or dorsal surfaces of the skeletons was not always clear. In one type of scale (usually dorsal), the overall shape is roughly circular and the ornamentation is composed of relatively short, semi-parallel striae. Additionally, where the longitudinal striae converge at the edge of the scale that is overlapped by imbricating scales, there generally is a net-like pattern of ornamentation formed by shorter, perpendicular striae (Fig 1B). In small specimens with tiny scales that type of ornamentation can resemble a starburst with few, nonparallel, radiating striae and less cross-hatching at the point of convergence. The second type of scale (usually ventral) is more elongate and oval in shape. The striae are accordingly elongate, generally parallel to one another, and there is no cross-hatch pattern where the striae converge (Fig 1C). Both types of scales are unmodified during ontogeny although they do increase in size along with the rest of the body (i.e., larger animals have proportionately larger scales), and the ornamentation becomes stronger. However, the largest individuals of *M. pelikani* also developed a heavy ridge that curves along the edge of the scale (Fig 1B and 1D) where it may have attached or embedded into the dermis, if not completely embedded. The ridge is thick, broad, and smooth and is not the same as the ‘ridges’ described by Milner [15], which are thick lines produced along the edges of the scales where they overlap when preserved in place. When more than one row of scales is preserved in situ, the ‘ridges’ [15] formed by the overlap make the scales appear square (Fig 1E). When isolated, however, it is apparent that the scales are either round or oval. The heaviest ridges usually are found on the scales from the ventral surface of the body.

**Fig 2:**
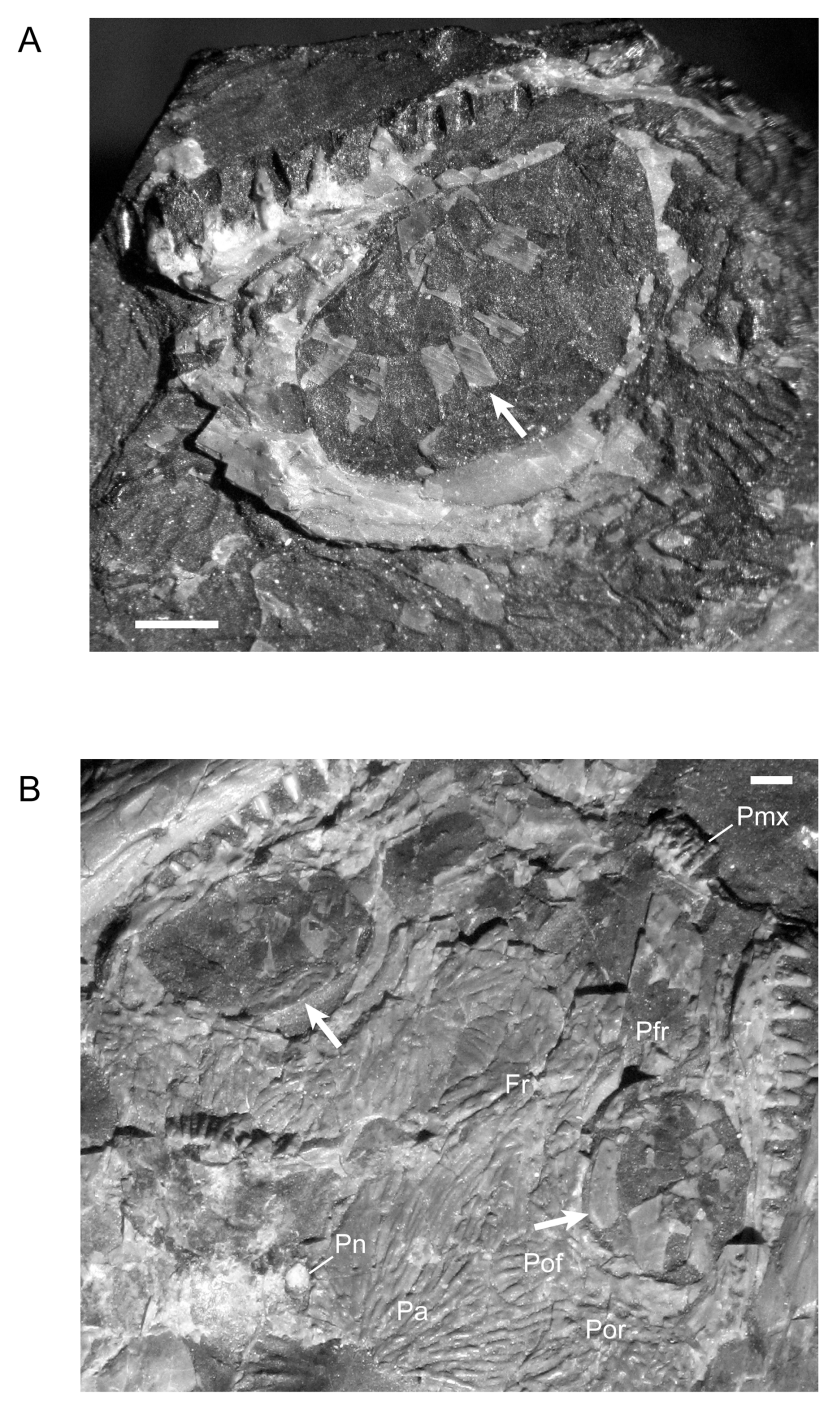
Dermal ossifications of *M*. *pelikani*. A. Scleral ossicles of M3322 (National Museum Prague, (previously Narodini Museum), Prague, Czech Republic); arrow points to single ossicle. B. Palpebral bones and scleral ossicles; arrows points to palpebral bones. Scale bars = 1mm.

Bony scleral ossicles and palpebral elements are present in *M*. *pelikani*. The scleral ossicles are fragile or weakly ossified because, even though they are present in individuals spanning the full size range known for *M*. *pelikani*, they are preserved only rarely [16]. The scleral ossicles are rectangular and form a ring inside the orbit [1]. I never observed a complete ring, but one specimen (M3322; National Museum Prague, (previously Narodini Museum), Prague, Czech Republic) possesses 11 ossicles in semi-articulation (Fig 2A). The ‘palpebral cup’ also was described by Carroll and Gaskill [1], although only from impressions, and the element was reported to be round and convex. In one individual (M3322), however, the palpebral is preserved in bone and shown to be a relatively robust element of oblong shape (Fig 2B), much more like the palpebrals described in other lepospondyls [1]. The palpebral exhibits sculpture similar to that of the dermal roofing elements (see below) and usually is found in a position dorsal to or overlying the scleral ossicles, suggesting that it forms within the eyelid, as in some living crocodilians and lizards. Clear preservation of the palpebral is even less common than for the scleral ossicles.

**Fig 3:**
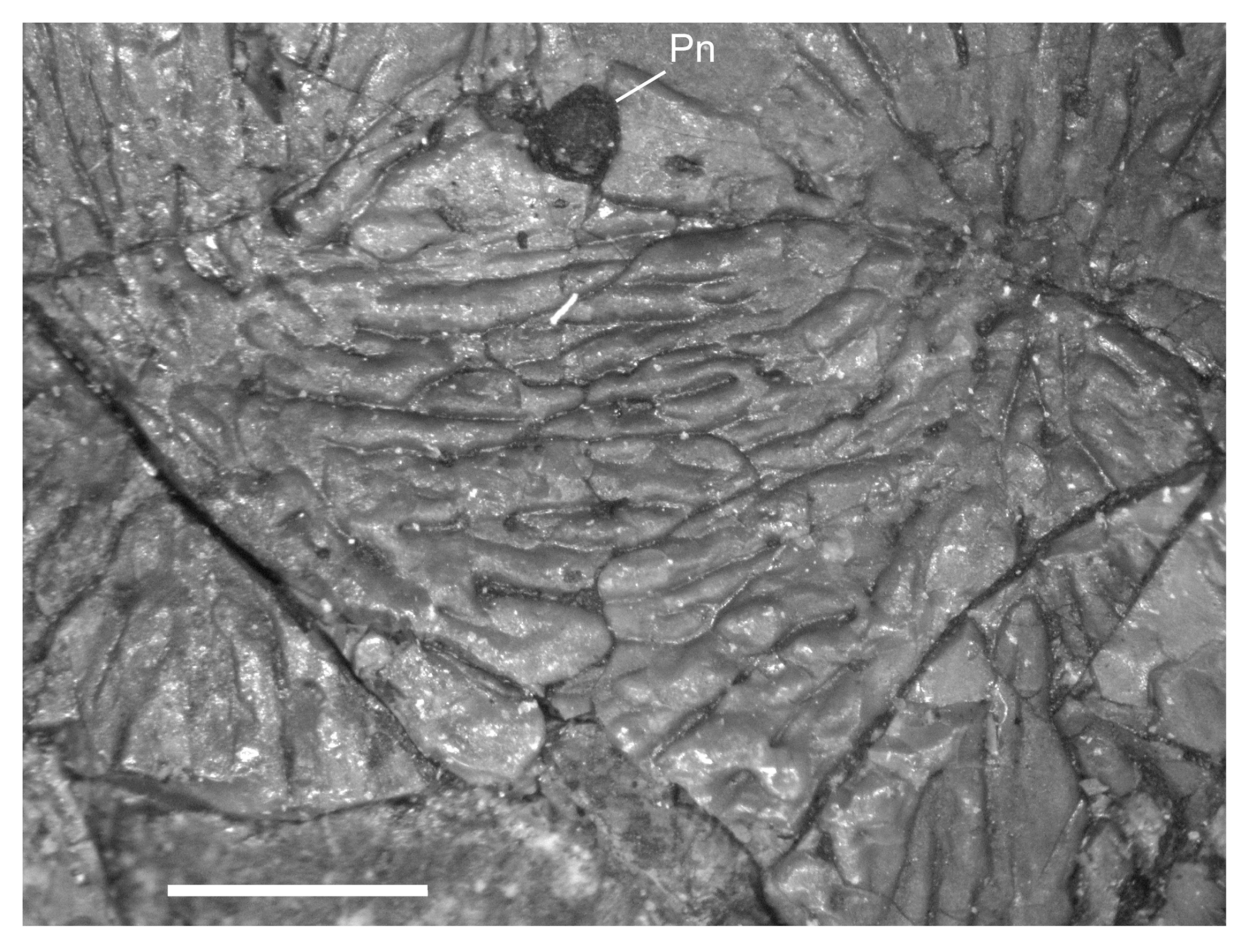
Postparietals of *M*. *pelikani*. Rough sculpture displaying ‘knitting’ across the midline suture of the contralateral postparietals of St.201 (Narodini Museum, (now National Museum Prague), Prague, Czech Republic). Scale bar = 1mm.

As partially described by Carroll and Gaskill [1], dermal sculpture is modified during growth. In the smallest specimen with a preserved skull (skull length = 7mm) the parietal, tabular, squamosal, and dentary are marked with deep, widely spaced, and straight grooves that radiate from the ossification centers. Except for the frontal, which exhibits faint striae, all other dermal skull elements have not yet begun to develop ornamentation. This lag is characteristic of a pattern of sculpture elaboration found in many individuals of *M*. *pelikani* in which the parietal, tabular, squamosal, post-parietal, post-orbital, and articular exhibit the most prominent dermal ornamentation, followed by the frontal, jugal, and postfrontal, which appear moderately sculptured in comparison. The development of the sculpture on the nasal, lacrimal, prefrontal, postfrontal, maxilla, and premaxilla is delayed further and these elements remain relatively smooth in earlier growth stages. During the next stage of sculpture development (skull length ~10–13mm) weak crenulations form at the ossification centers of the most strongly ornamented elements and the once straight grooves show more sinuous traces. During later stages of growth (skull length ~14–17mm), these grooves are more densely spaced and tend to bifurcate into multiple pathways. Additionally, many grooves anastomose, resulting in the appearance of alternating grooves and ridges frequently described in the literature (e.g., [1,47]). In specimens of still larger size (skull length ~18mm+) the crenulations at the ossification centers become so rough that they appear fragmented into many small islands of raised ornamentation. Moreover, the radiating grooves and ridges deepen and thicken while converging to become continuous with those radiating from neighboring dermal elements (Fig 3). Further development of the sculpture produces more densely packed crenulations and ridges that resemble a net-like pattern observed only in the largest individuals (skull length ~25mm+).

**Fig 4:**
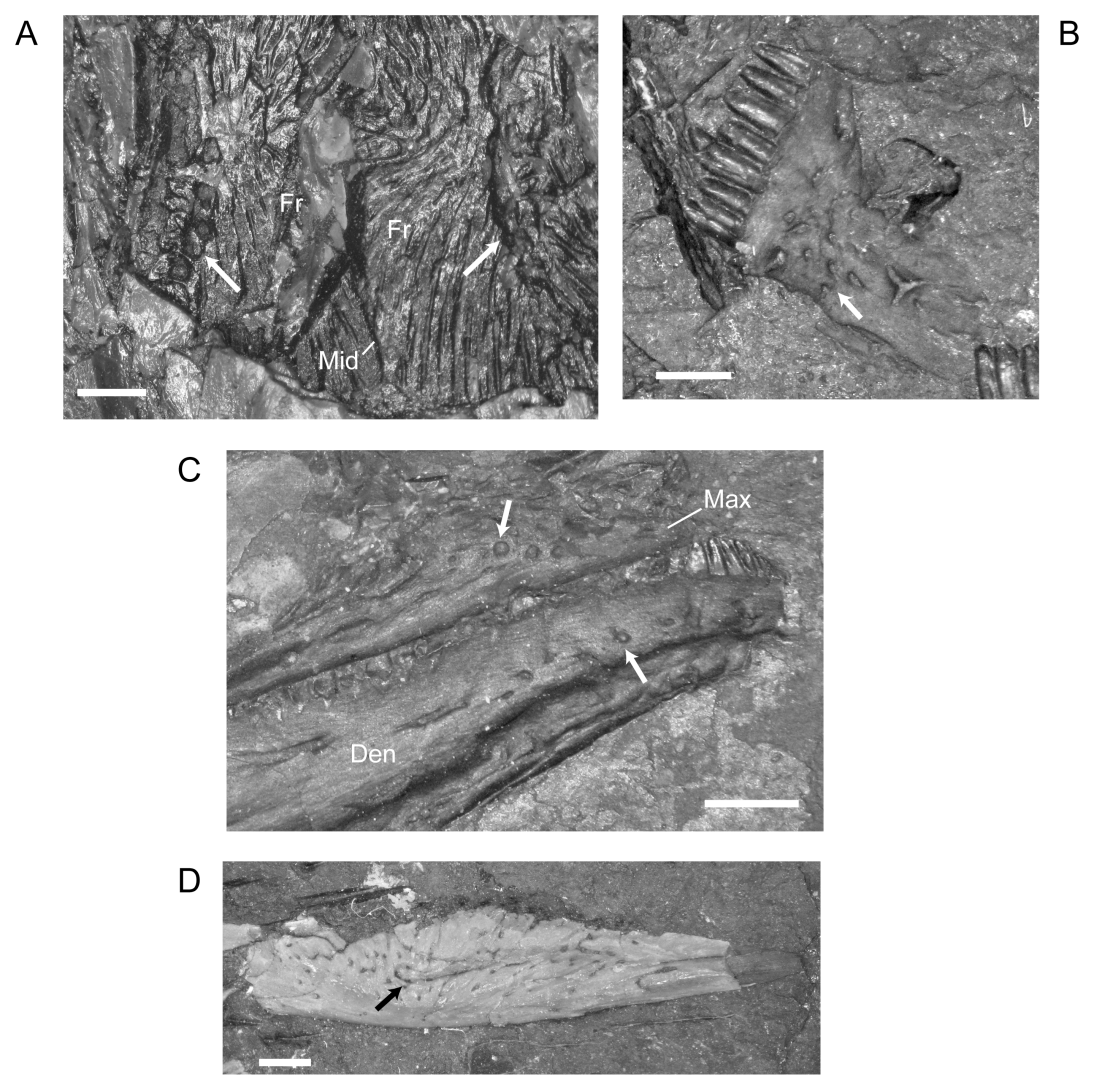
Lateral lines of *M*. *pelikani*. Arrows point to pits and grooves. A. Pits and grooves on paired frontals of NHMW1894-2399 (Naturhistorisches Museum, Vienna, Austria); Impression of dorsal view, anterior up, grooves lateral. B. Pits on premaxilla of CGH3018 (Narodini Museum, (now National Museum Prague), Prague, Czech Republic); Impression of anterior view (3D caused by lighting), teeth denote ventral surface, medial down. C. Pits on maxilla and dentary of NHMW1983_32_49; Impression of lateral view (3D caused by lighting), anterior to the right, dorsal up. D. Groove on articular of CGH5; lateral view of bone, anterior to the right, dorsal up. Scale bars = 1mm.

Traces of lateral lines were present in all specimens of *M*. *pelikani* examined, suggesting that the system is retained throughout ontogeny. However, previous descriptions of the lateral lines as a clear ‘lyre pattern’ resembling that of temnospondyls [1] are exaggerated. Especially in the larger individuals in which sculpture patterns are rough, evidence for lateral lines may occur only as pits rather than deep grooves. In a few cases, even the pits are visible only along the lateral edge of the frontal where that bone contacts the prefrontal and postfrontal. Usually, however, as described by Carroll and Gaskill [1], the pits are contained within a shallow groove and are present on the lacrimal, frontal, prefrontal, postfrontal, jugal, postorbital, articular, and dentary. In addition, I observed well developed clusters and lines of small foramina along the premaxilla and maxilla, respectively, which are probably sensory canals and possibly connected to the lateral line system, although Carroll and Gaskill [1] suggested that these bones did not contain lateral line receptors. I did not observe any pits or grooves on the quadratojugal or squamosal, although such structures were reported previously [1]. Therefore, it appears that the lateral line system was restricted to a ring around the orbit and continued for a short distance posteriorly along the medial margin of the jugal, the lateral surface of the dentary and articular, and as small sensory pits along the maxilla and premaxilla (Fig 4).

**Fig 5:**
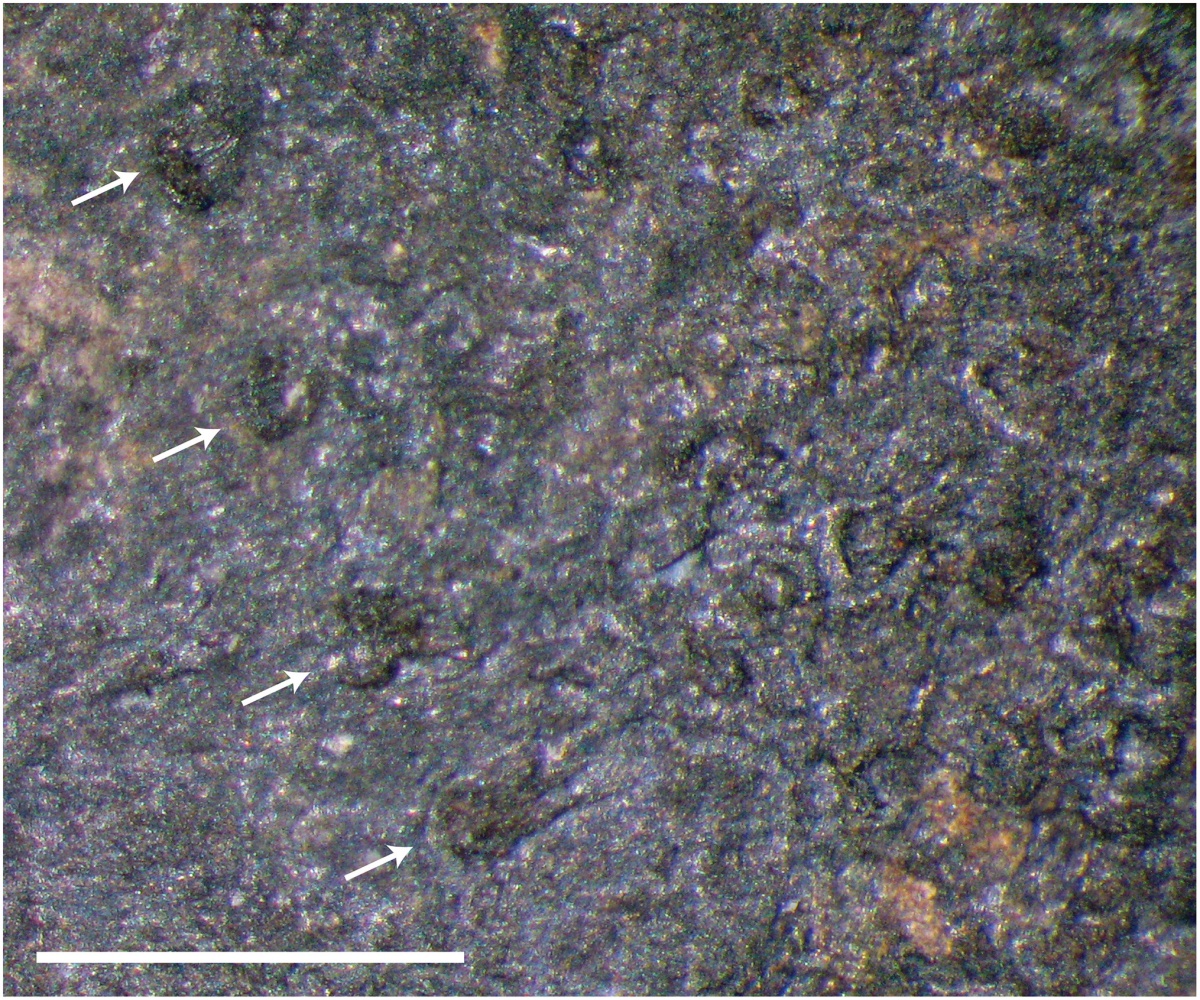
Branchial plates of *M*. *pelikani*, NHMW1898_X_29. Arrows point to plates. Ventral view, anterior up, medial to the left. Note the triangular processes projecting laterally from the ventral-most branchial plate. Scale bar = 1mm.

A major point of historical contention concerning *Microbrachis* is whether or not the taxon possessed external gills. I studied nearly all known specimens of *M*. *pelikani* and examined the structures previously identified as branchial plates [1]. The structures are present in specimens spanning nearly the total size range observed for the taxon, suggesting that they persisted throughout ontogeny, although overall their preservation is relatively rare [16]. My interpretation is that the bony structures probably are branchial plates, although the presence of small, round or octagonal scales in the same location in other microsaurs like *Pantylus* and *Saxonerpeton* ([1]; pers. obs.), as well as the disorganized arrangement of the plates in *Microbrachis*, cast some doubt. The best evidence for the identification of the structures as branchial plates comes from NHMW1898_X_29 (Naturhistorisches Museum, Vienna, Austria), an individual that exhibits such good preservation that under high magnification three triangular-shaped structures, perhaps denticles, can be observed projecting along one side of the plates (Fig 5). In that individual and other specimens in which the plates are present, only a shallow impression remains, attesting to the fragility or perhaps low level of ossification of these structures. Impressions are usually round or oval, and in better-preserved specimens there is a raised spot at the center, which produces a donut or ring-shaped element in casts. The structures frequently are associated with the interclavicle, because both are visible mainly when specimens are preserved in ventral view. When present, the branchial plates generally are found close to the edge of the interclavicle, located between it and the anteriormost vertebrae, when the interclavicle has been displaced from the midline.

#### Snout and Dorsal Roof Elements

**Fig 6:**
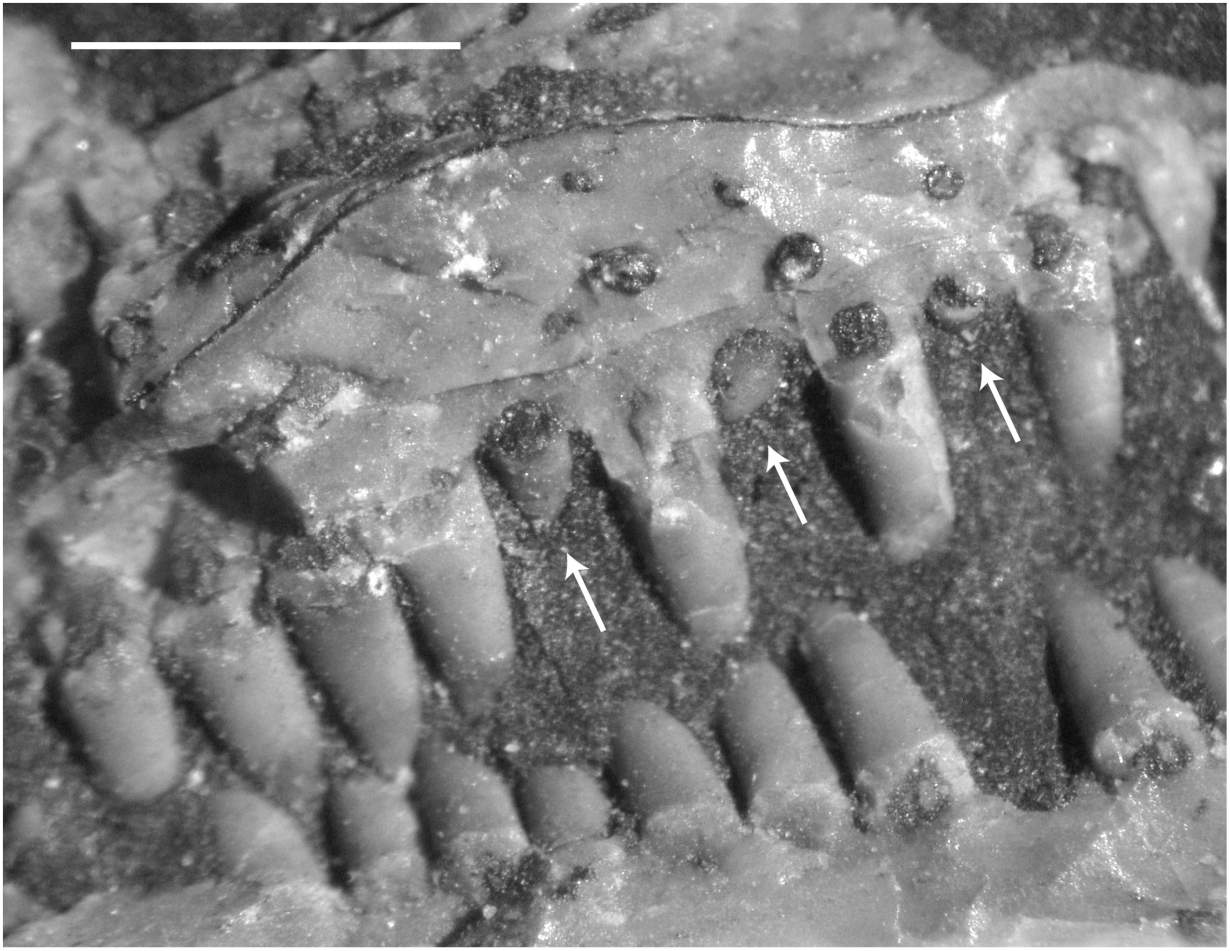
Maxillary replacement teeth of *M*. *pelikani*, M3322. Maxilla is dorsal tooth row, dentary is ventral tooth row. Lateral view, anterior to the right. Arrows point to replacement teeth. Scale bar = 1mm.

Contacts among cranial elements are consistent in individuals of all sizes. The premaxilla and maxilla do not show changes during ontogeny. I recorded a maximum of eight teeth in the premaxilla, although Carroll and Gaskill [1] reported seven. Previous descriptions of maxillary tooth shape and patterns are mostly accurate, as are reports of the variation in maximum tooth number (19–22). However, although it was noted previously that the more posterior maxillary teeth are smaller than the remaining teeth [1], I found that in specimens in which the entire tooth row is visible, both the anterior and posterior (mesial and distal, respectively) maxillary teeth are smaller than those located in the middle (approximately teeth 6–16). Additionally, many individuals exhibit a slight recurvature of the tips of the maxillary teeth, though this feature is unrelated to size. One specimen, M3322, preserves maxillary replacement teeth (Fig 6). The observed pattern may be coincidental, but replacement appears to occur in alternating teeth, with all replacement teeth at the same stage of eruption. Supporting this interpretation is a similar pattern of alternating replacement previously reported for the dentary of *Microbrachis* [1]. However, the replacement teeth described in Carroll and Gaskill [1] consist only of their distal tips, interpreted to be anchored to the dentary by connective tissue. The maxillary replacement teeth instead show the distal tips as emerging directly along the surface of the tooth row, from within sockets. The alternating pattern of replacement suggested for *M. pelikani* may be plesiomorphic for tetrapods and differs from the derived and rare simultaneous replacement reported for the microsaurs *Euryodus* and *Cardiocephalus* [53].

**Fig 7:**
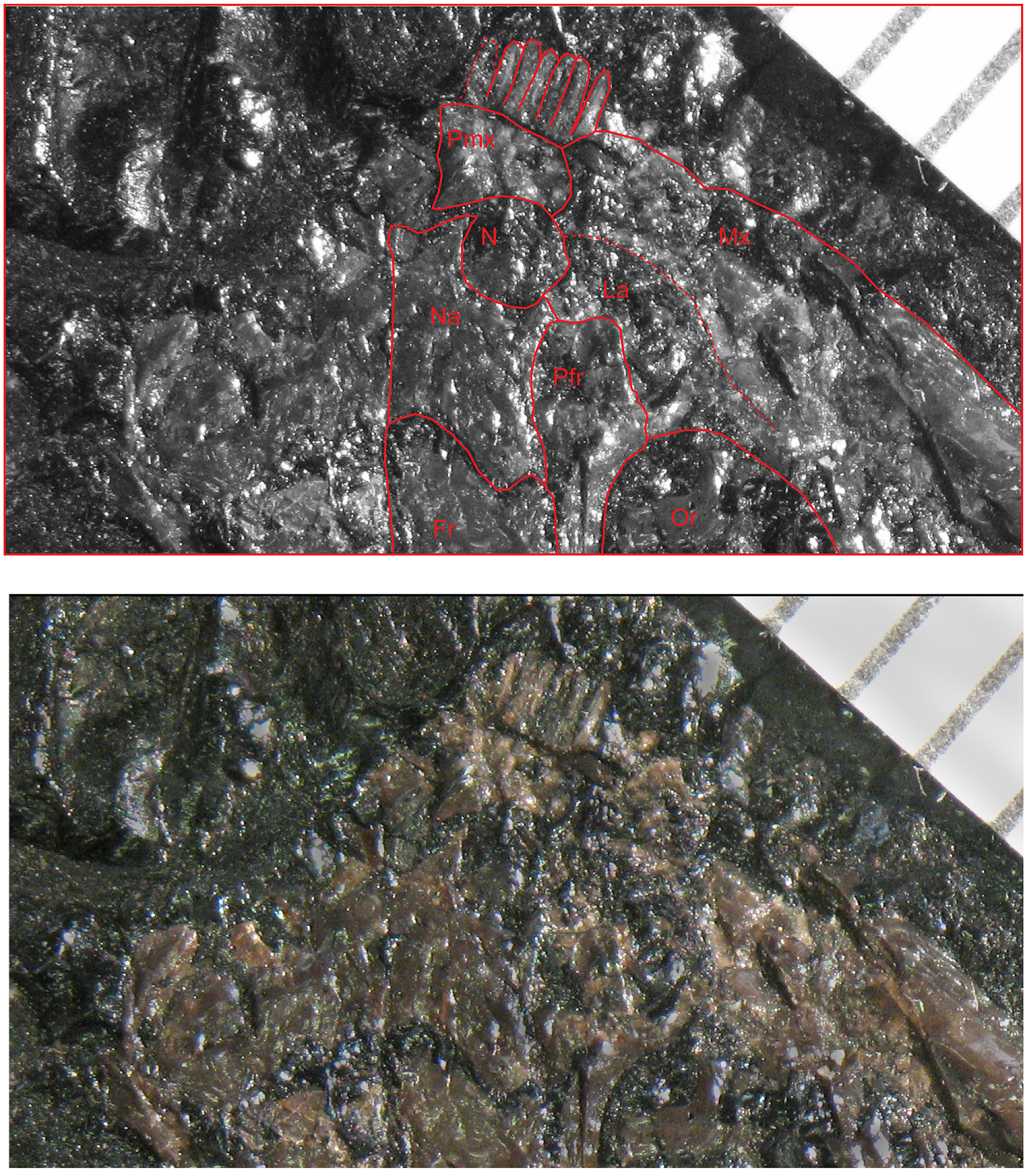
Narial region in *M*. *pelikani*. Dorsal view of bones in St.200, anterior is up, lateral to the right. Top image includes line drawing interpretation of sutures observed in original photograph (bottom). Fr, frontal; La, lacrimal; Mx, maxilla; N, naris; Na, nasal; Or, orbit; Pmx, premaxilla. Scale bar is in 1 mm increments.

**Fig 8:**
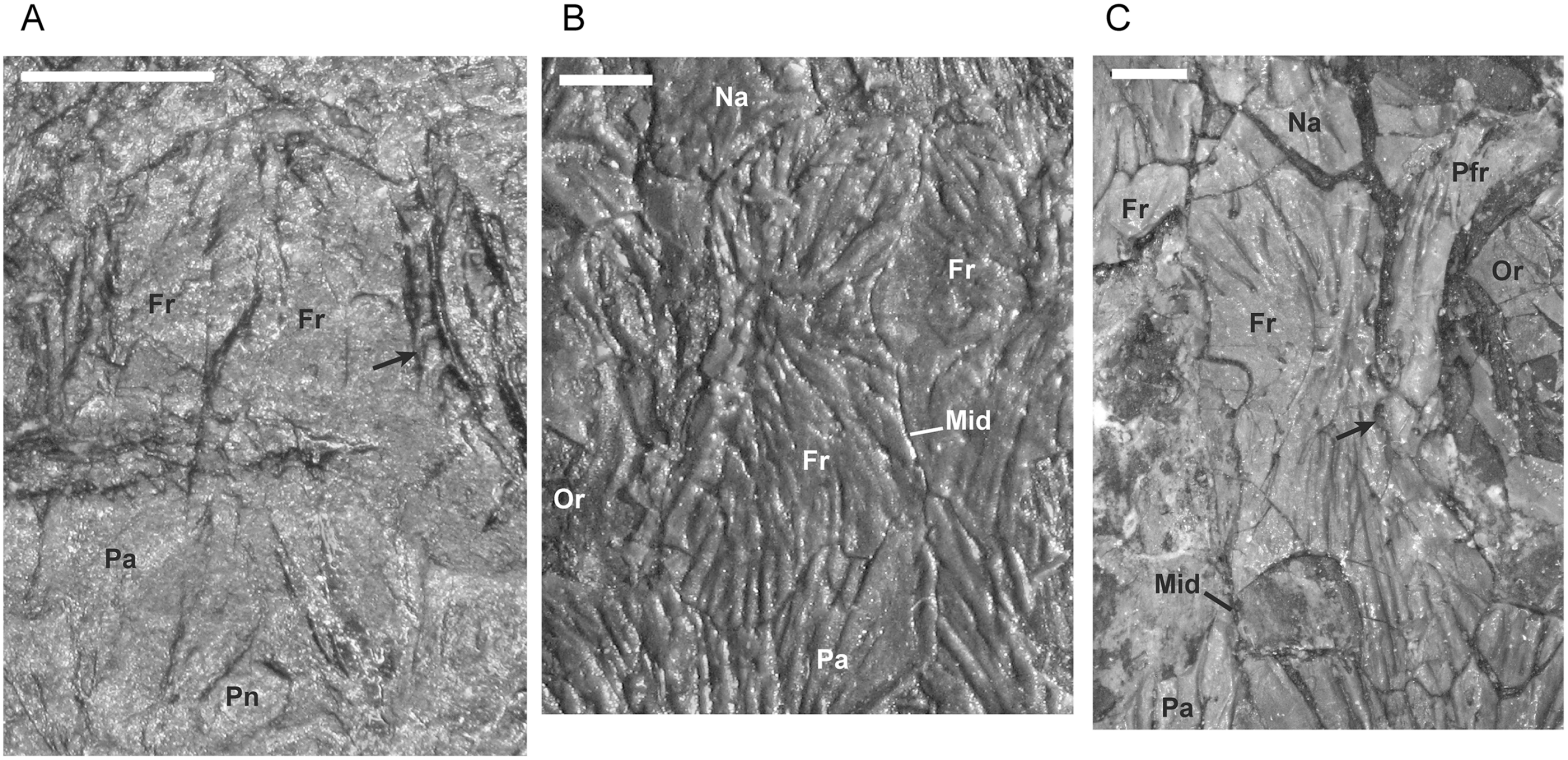
Shape change in the frontal during development of *M*. *pelikani*. Arrows point to lateral line pits. A. Small individual, NHMW1893_32_66. Dorsal view, anterior up. B. Medium individual, M1700. Dorsal view, anterior up. C. Large individual, St.201. Dorsal view, anterior up. Fr, frontal; Mid, midline suture; Na, nasal; Or, orbit; Pa, parietal; Pfr, prefrontal; Pn, pineal foramen. Scale bars = 1mm.

The portion of the skull surrounding the external naris is often the least well preserved area, leading to frequent speculation about the narial margins. Despite having a larger sample size than those used in previous studies [1,9], I did not find evidence of a septomaxilla in *M*. *pelikani*. The element may be unossified or perhaps weakly ossified, resulting in poor preservation potential. The only new information that I can provide about the nasal is a more complete description of the contact with the lacrimal. As hinted at in illustrations by Carroll and Gaskill ([1] reference figures 77,78) and Vallin and Laurin ([9] reference figures 2,3), but not discussed by them, the nasal has a short, squared process that projects laterally to meet the anterior end of the lacrimal (Fig 7). That contact contributes to the posterior margin of the external naris and is located anterior to the prefrontal, the latter of which is, thus, excluded from the external naris. As reflected in previously published Thin Plate Splines (TPS; [17]), the anterolateral edge of the nasal that curves posteriorly around the naris to form the lacrimal process becomes relatively shorter in larger individuals, suggesting that the diameter of the naris decreases proportionately as well. The only other change exhibited by the nasal is a transition from angular to rounded edges during growth. A similar change occurs in the frontals, which start out with relatively straight margins in small specimens, but the margins become more sinuous with increased growth (Fig 8).

**Fig 9:**
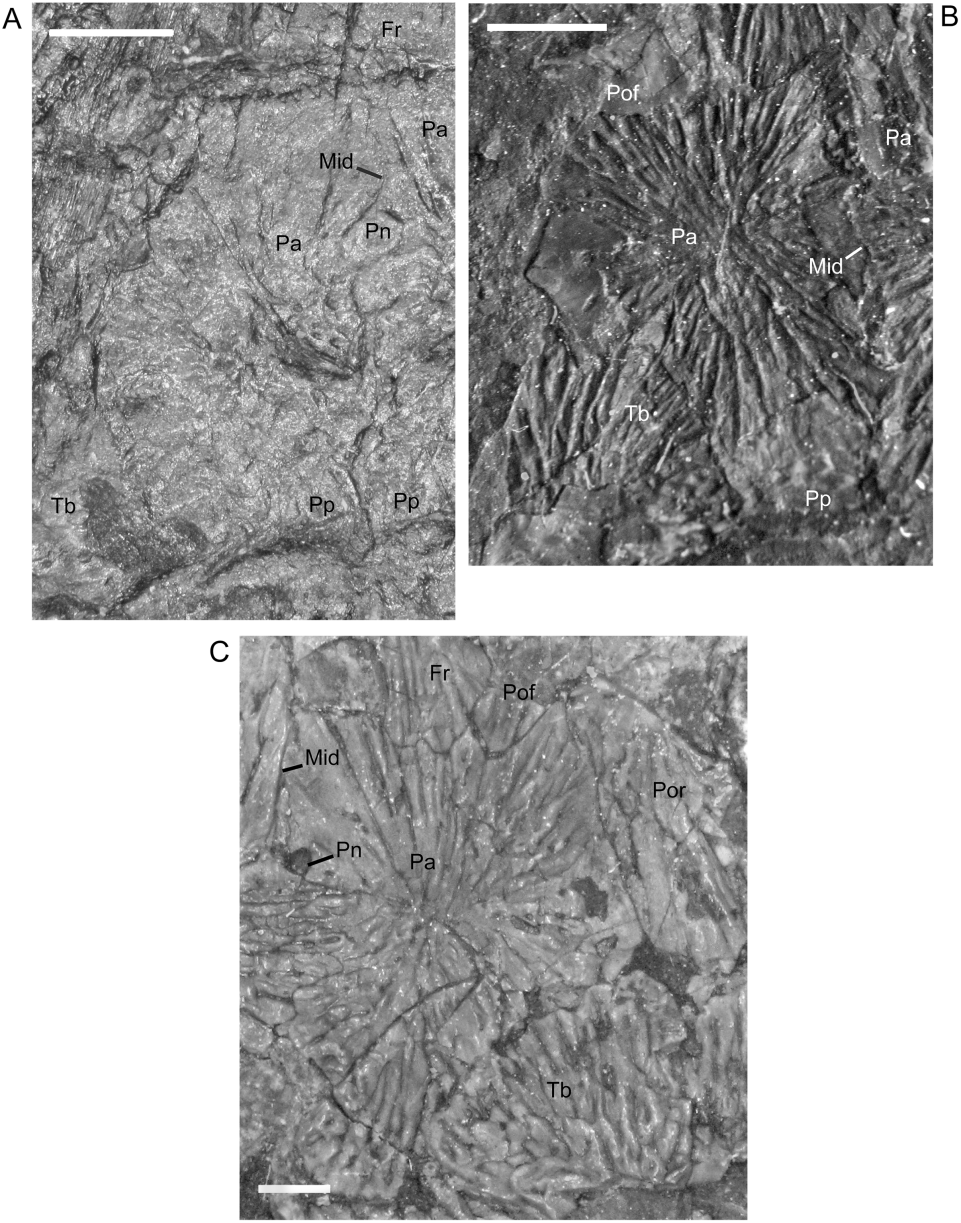
Shape change in the parietal during development of *M*. *pelikani*. A. Small individual NHMW1893_32_66. Dorsal view, anterior up, left parietal. B. Medium individual, MB.Am.833 (Museum für Naturkunde, Leibniz-Institut für Evolutions- und Biodiversitätsforschung, Berlin, Germany). Dorsal view, anterior up, left parietal. C. Large individual, St.201. Dorsal view, anterior up, right parietal. Fr, frontal; Mid, midline suture; Pa, parietal; Pn, pineal foramen; Pof, postfrontal; Por, postorbital; Pp, postparietal; Tb, tabular. Scale bars = 1mm.

In the smallest specimen, the parietal also has relatively straight margins in addition to a less laterally extensive lateral lappet (Fig 9A). In larger individuals, the sutures become increasingly sinuous and, as reflected in TPS analysis [17], the lateral lappet expands in size (Fig 9B). In most specimens, regardless of size, the pineal foramen is relatively large and located closer to the frontal-parietal suture than to the postparietal-parietal suture. Traditional morphometrics suggested that there is a great deal of individual, rather than ontogenetic, variation in both the location and size of the pineal [17]. Contra Vallin and Laurin [9], a pineal is clearly visible in all specimens in which poor preservation is not a factor. The specimen that Vallin and Laurin [9] suggested to be lacking a pineal (MB.Am.815 (Museum für Naturkunde, Leibniz-Institut für Evolutions- und Biodiversitätsforschung, Berlin, Germany); Fig 2B) is actually a modified plaster cast that exhibits many errors in the emphasis of sutures and the shape of cranial elements.

Carroll and Gaskill [1] noted that the dorsal exposure of the postparietal increases with growth. Mistakenly applying that observation to the entire dorsal lamina (versus the occipital portion), including the anterior margin that underlies the parietal, Vallin and Laurin [9] did not observe an ontogenetic trend. I do not agree with either previous interpretation and suggest that the dorsal exposure of the dorsal flange tends to be proportionately smaller in larger individuals, although there is much individual variation and possibly deformation resulting from crushing of the occipital flange. Likely contributing to the past discrepancy is the tendency for the sculpture on the parietal and postparietals to knit together, obscuring the suture between the two elements. However, after examining a large number of individuals, it appears that the usual location of that suture is at the same level as the inflection in the medial margin of the tabular, where the suture with the postparietal along the posterior skull table starts out vertically and then angles laterally to subsequently follow the ventral edge of the lateral lappet of the parietal. Additionally, the contact between the parietal and tabular is not overlapping, in contrast to the situation in *H*. *longicostatum* in which the tabular sits on a broad parietal shelf (see below).

Previously it was reported that a few specimens of *M*. *pelikani* lacked the usual contact between the tabular and postorbital as a result of intervening contact between the squamosal and parietal [9]. However, I examined the potentially polymorphic specimen (MB.Am.825; [9], Fig 2A) and there is a contact between the tabular and postorbital, though it is less extensive than usual. The specimen is not well preserved; the tabular and parietal sutures suggest slight displacement, and the tabular appears to have slightly overridden the squamosal. Additionally, the elements forming the skull table show a small degree of anteroposterior elongation, probably due to deformation, that is not reflected in the drawing by Vallin and Laurin ([9] reference figure 2A), and the suture between the tabular and parietal is figured incorrectly.

#### Cheek and Circumorbital Elements

Skulls frequently are preserved with one cheek splayed out laterally while the other folds under the skull, consistently separating along the tabular-squamosal and postorbital-jugal sutures. That separation suggests that a zone of weakness exists between tabular-squamosal and postorbital-jugal, and that the contacts between those elements are not sutural. In other lepospondyls, such as *Pantylus*, the cheek elements articulate via extensively overlapping contacts and are only loosely connected to the dorsal roof elements (pers. obs.). Although the articulation with the skull table is similarly loose in *M. pelikani*, the cheek elements do not appear to have broad shelves that underlie neighboring elements. The contacts between cheek elements are not tightly sutured either, forming abutting articulations. Much of the rest of the skull also does not exhibit tightly sutured articulations, though elements may overlap. Only the contact between the contralateral parietals and postparietals, and perhaps between the tabular and parietal, exhibit tight or even interdigitating sutures, and that association appears to develop with increased development of the sculpture, although it also is visible on the unornamented, ventral surface of the bones.

In the circumorbital region, previously the lacrimal was reconstructed as falling short of the external naris, and an hypothesized septomaxilla was estimated to fill the space between the lacrimal and naris [1,9]. However, such an extensive exterior exposure of the septomaxilla is not observed in other ‘microsaurian’ lepospondyls, and as noted above, a septomaxilla is not reported, nor obviously present, in *M*. *pelikani*. In the specimens that I observed, the lacrimal extends far anteriorly to border the external naris, although contact between the lacrimal and a lateral process of the nasal excludes the prefrontal from the naris (Fig 7).

Outside of shape changes previously revealed by geometric morphometric analysis [17], the prefrontal, postfrontal, postorbital, jugal, and quadratojugal do not exhibit marked ontogenetic changes. However, the articulation of the posterior circumorbital elements was not described fully in the past. The postfrontal, postorbital, and jugal articulate with one another anteriorly via interlocking contacts. The postorbital (lateral) process of the postfrontal extends laterally to lie anterior to the anteromedial edge of the postorbital and prevent that portion of the postorbital from participating in the orbit. Similarly, the jugal (lateral) process of the postorbital projects laterally to sit anterior to the anteromedial edge of the jugal, preventing that surface from forming the orbit. That arrangement of posterior circumorbitals is identical to the pattern of articulation present in *Pantylus* ([1]; pers. obs.).

#### Palate, Braincase, Quadrate, Stapes

There are small denticles located on the ventral surface of the vomer, palatine, ectopterygoid, pterygoid, and the cultriform process of the parasphenoid [1]. As previously described by Steen [46] and Carroll and Gaskill [1], the denticles on those surfaces always are divided into parallel, longitudinal rows by thin, but prominent, ridges. That pattern, present in individuals of all sizes, is distinctive for *M. pelikani* and can be used to distinguish that taxon from other lepospondyls found at Nýřany. The organization of the denticles forms early in ontogeny; however, in a few of the smaller skulls, the ridges on the cultriform process are present and distinct, but the denticles themselves are not well-developed or are absent, suggesting either a developmental sequence in which the ridges form prior to the denticles, or poor preservation potential for the denticles.

**Fig 10:**
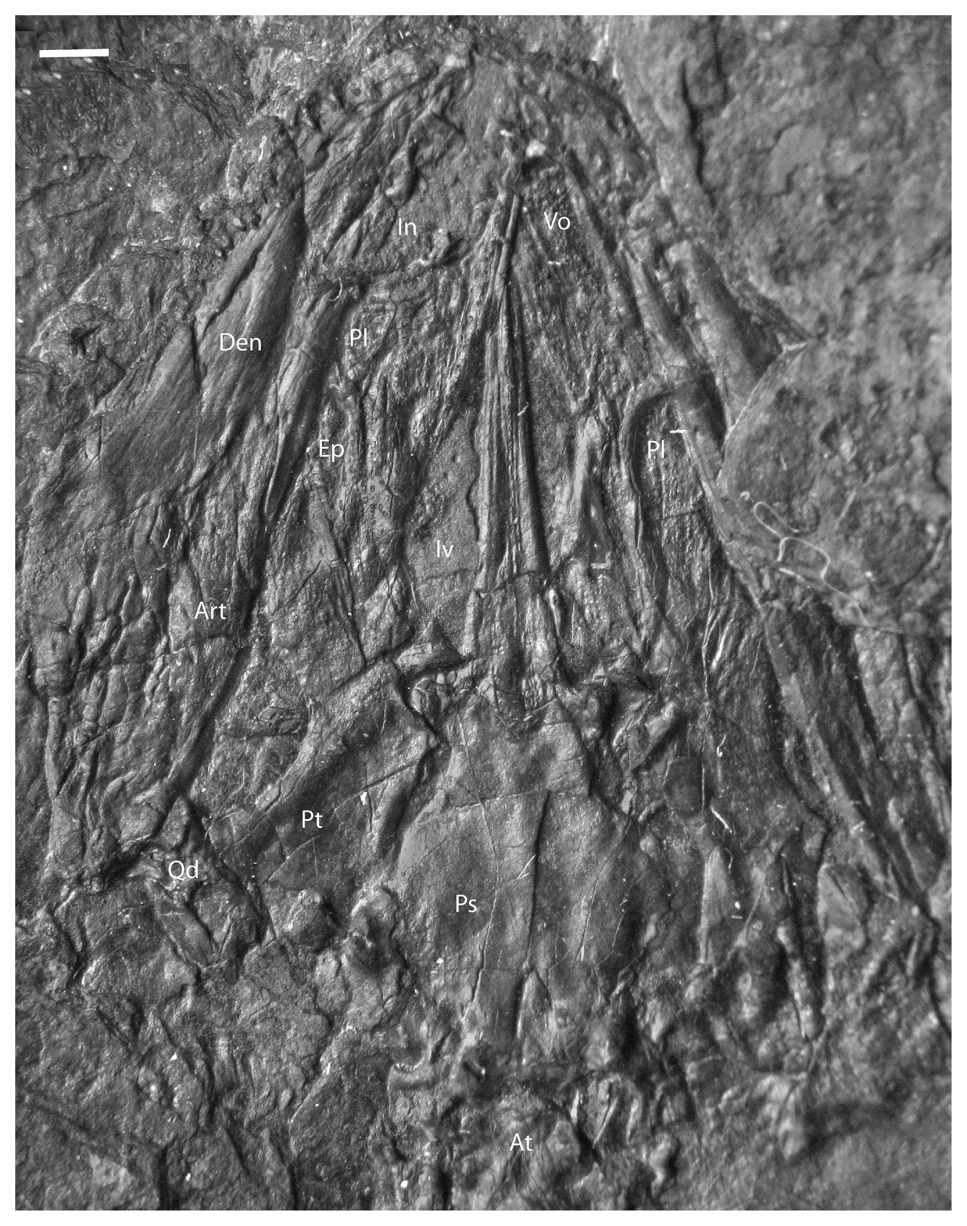
Palate of *M*. *pelikani*, MB.Am.821, showing complete pterygoid. Specimen is an impression, but the angle of the lightening causes a 3D effect. Art, articular; At, atlas; Den, dentary; Ep, ectopterygoid; In, internal naris; Iv, interpterygoid vacuity; Pl, palatine; Ps, parasphenoid; Pt, pterygoid; Qd, quadrate; Vo, vomer. Scale bar = 1mm.

No other ontogenetic changes were observed in the palate. However, the specimens that I examined agree with the interpretation that the contralateral vomers meet at the midline and are situated between the pterygoids and premaxillae ([9] reference figure 5A]. That interpretation contrasts with the reconstruction by Carroll and Gaskill ([1] reference figure 77D), which suggested that the pterygoids project far anteriorly to reach the premaxillae and intervene between the paired vomers. The clearest view of the arrangement is found in MB.Am.821 in which the vomer, pterygoid, and parasphenoid are all in place (Fig 10). Similar semi-articulation is observed in MB.Am.838, although the cast figured by Vallin and Laurin ([9] reference figure 4B] is missing some features. In a more complete cast, the right vomer articulates with the anterior end of the pterygoid and extends farther anteriorly than the later. Moreover, the median edge of the vomer is free along the midline for articulation with the contralateral element. Still, the vomer is disarticulated in nearly all known individuals and, even in MB.Am.821 and MB.Am.838, the region where the vomer should contact the premaxilla is not well-preserved and the contrateral vomer is missing. With the material available, it not possible to reconstruct the relationship between the premaxillae and the palate with complete confidence.

**Fig 11:**
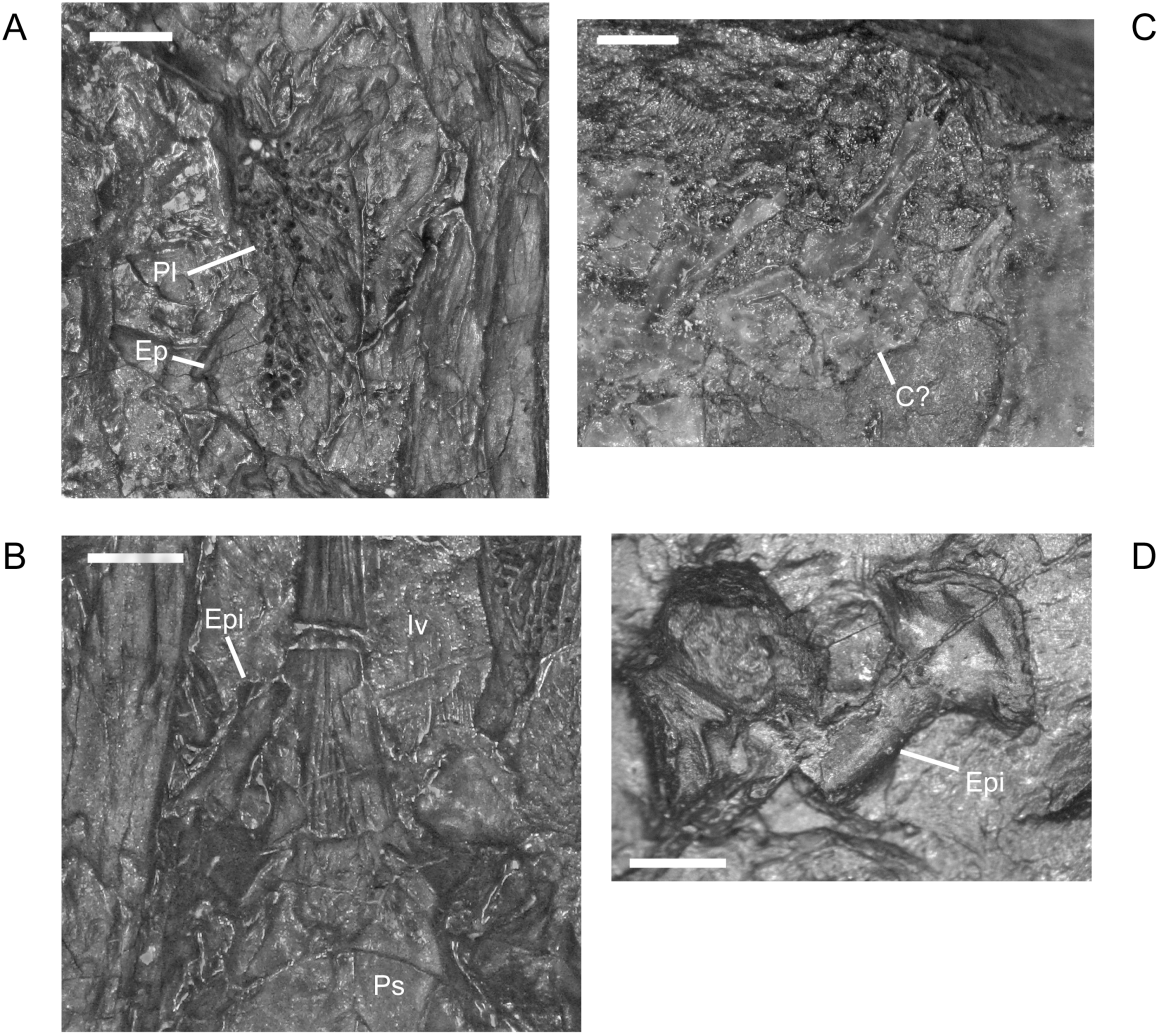
Palate elements, *M*. *pelikani*. A. CGH727 (Narodini Museum, (now National Museum Prague), Prague, Czech Republic); isolated right palatine and ectopterygoid. Ventral view, anterior up, lateral to the right. B. CGH727; displaced right epipterygoid. Ventral view, anterior up. Specimen is impression, but the angle of the lighting causes a 3D effect. C. CGH251; ‘epipterygoid’ figured by Carroll and Gaskill [1]. Probably a circumorbital. D. M1689. Potential isolated epipterygoid. C?, circumorbital?; Ep, ectopterygoid; Epi, epipterygoid; Iv, interpterygoid vacuity; Pl, palatine; Ps, parasphenoid. Scale bars = 1mm.

Specimen CGH727 preserves a disarticulated palatine (Fig 11A). That element is relatively elongate and narrow, more similar to reconstructions by Carroll and Gaskill [1] than to those of Vallin and Laurin [9]. The vomerine (medial) process of the palatine is robust and projects relatively far medially. The largest denticles on the palatine are located at the base of the maxillary process, posterior to the smooth, U-shaped anterior edge that forms the posterior margin of the choana. Additionally, the disarticulated palatine appears to be in contact with an ectopterygoid, a much shorter and rounder bone. Both elements exhibit a straight lateral margin for contact with the maxilla. The base of an epipterygoid, with the dorsal stem broken and missing, appears to be preserved underneath the ectopterygoid.

Previously, an articulated epipterygoid was identified and figured by Steen ([46] reference figure 16C’). That particular specimen (Plzeň Museum 676–677) was unavailable to me, but I located an additional specimen exhibiting the epipterygoid in semi-articulation (Fig 11B). A proposed isolated epipterygoid also was identified previously ([1] reference figure 79D). The skull of the specimen in question, CGH251, is badly preserved and the identified ‘epipterygoid,’ approximately the same size as the tabular, is preserved along with a group of disarticulated circumorbital, cheek, and posterior roofing elements. However, a disarticulated pterygoid also is located next to the ‘epipterygoid’, supporting the identification by Carroll and Gaskill [1]. If the element is not an epipterygoid, then it is likely a circumorbital element, specifically a broken lacrimal, because that is the only bone from that region not represented among the disarticulated elements. Identification as a circumorbital, rather than an epipterygoid, is supported by the smoothly curved edge, which traverses the whole length of the bone, including the portion composed of a lamina or base (Fig 11C). A clearer candidate for an isolated epipterygoid is present in M1689 (Fig 11D). That disarticulated element has a narrow, but robust process (broken), and an expanded, rounded base, conforming more with the morphology of the articulated epipterygoids. The element is too large to be the stapes or a broken transverse process. The epipterygoid of *M*. *pelikani* exhibits a small, round depression in the base of the element, which resembles the condition described for *Pantylus* [1,54].

**Fig 12:**
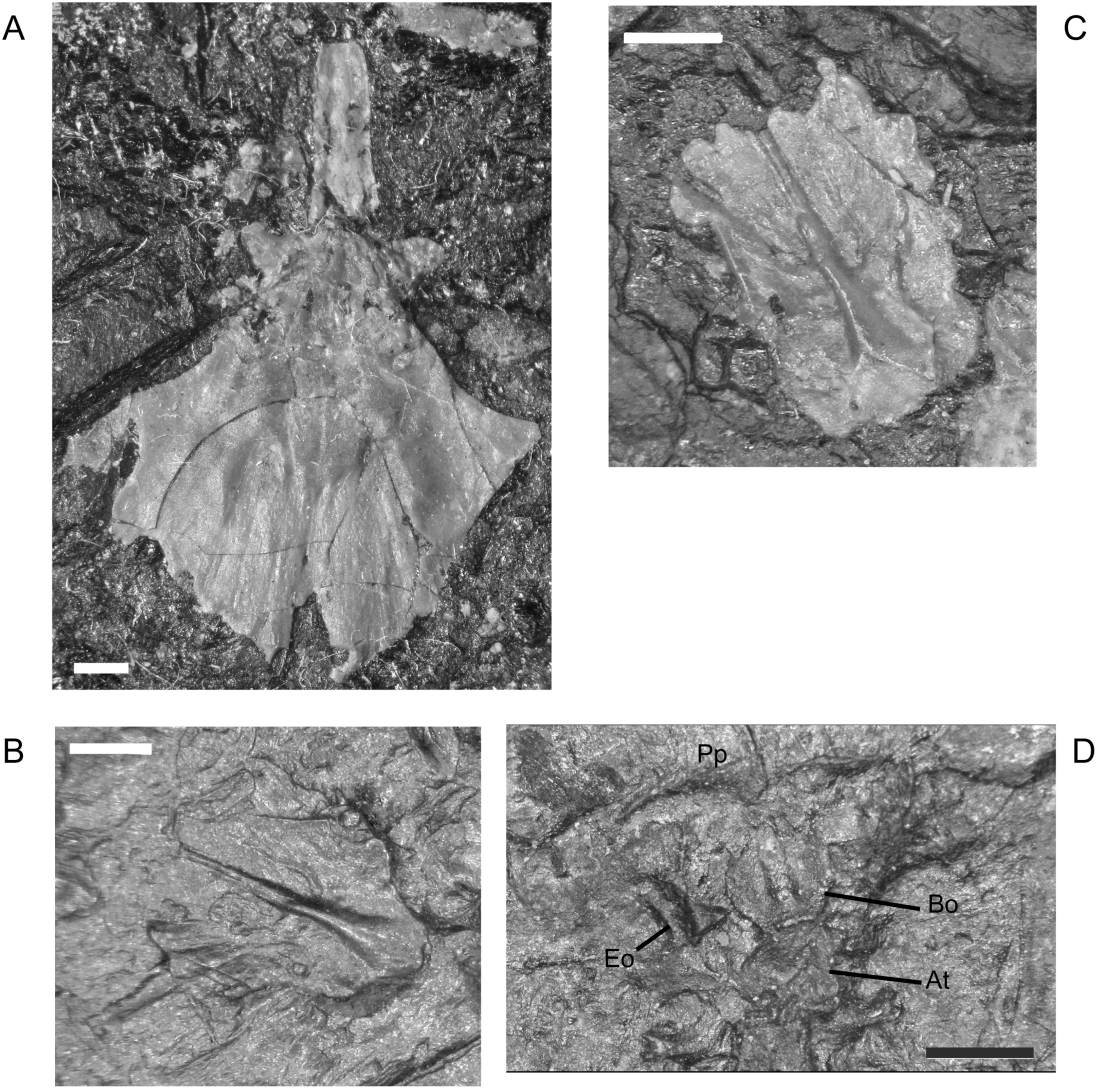
Palate and braincase elements, *M*. *pelikani*. A. CGH253, parasphenoid; ventral view, anterior up. Note paired depressions on parasphenoid plate. Bone. B. M1689, isolated basioccipital; dorsal view, anterior to the left. Metal cast. C. CGH256, isolated basioccipital; dorsal view, anterior up. Bone. D. NHMW1983_32_66, ventral view of occipital-atlantal articulation; anterior up. Impression. At, atlas; Bo, basioccipital; Eo, exoccipital; Pp, postparietal. Scale bars = 1mm.

When the parasphenoid is preserved in dorsal view, a pair of facets, open posteriorly, are present flanking either side of the midline (Fig 12A). Those facets likely facilitated articulation with the basioccipital. In a prior description of the parasphenoid, it was noted that in larger individuals, the element exhibits a bi-lobed posterior margin and a small posterolateral process [9]. I observed the bi-lobed morphology in all specimens in which the parasphenoid is preserved as bone and in most specimens in which only an impression remains. There is no clear association with size, except for a slight increase in the notch between lobes in larger individuals. Additionally, the short ‘posterolateral process’ described by Vallin and Laurin [9] appears to be an artifact of the broken lateral wing of the parasphenoid plate, which is a roughly triangular flange in all specimens that I examined.

The basioccipital was described [1], but never figured outside of articulation with the parasphenoid and exoccipitals. Based on the description provided by Carroll and Gaskill [1], I identified two isolated basioccipitals (Fig 12B and 12C). The smallest specimen that I examined exhibits a basioccipital in articulation with the exoccipitals and atlas (Fig 12D), demonstrating that the braincase achieves an unexpectedly high degree of ossification early in ontogeny. The basioccipital is roughly U-shaped with the curved edge inferred to be the posterior margin. The element is marked by three parallel ridges, one along each lateral edge and a third, more robust ridge along the midline. According to Carroll and Gaskill [1] those ridges would have articulated with the underlying parasphenoid and thus denote the ventral surface of the basioccipital. It is possible that the midline ridge, which is heaviest posteriorly, fits into the notch between the lobes at the posterior margin of the parasphenoid. That interpretation agrees with a prior observation that the midline ridge “protruded through the base of the parasphenoid” ([1] p.120). The lateral ridges of the basioccipital likely articulated with the facets present on either side of the midline notch of the parasphenoid (see above).

**Fig 13:**
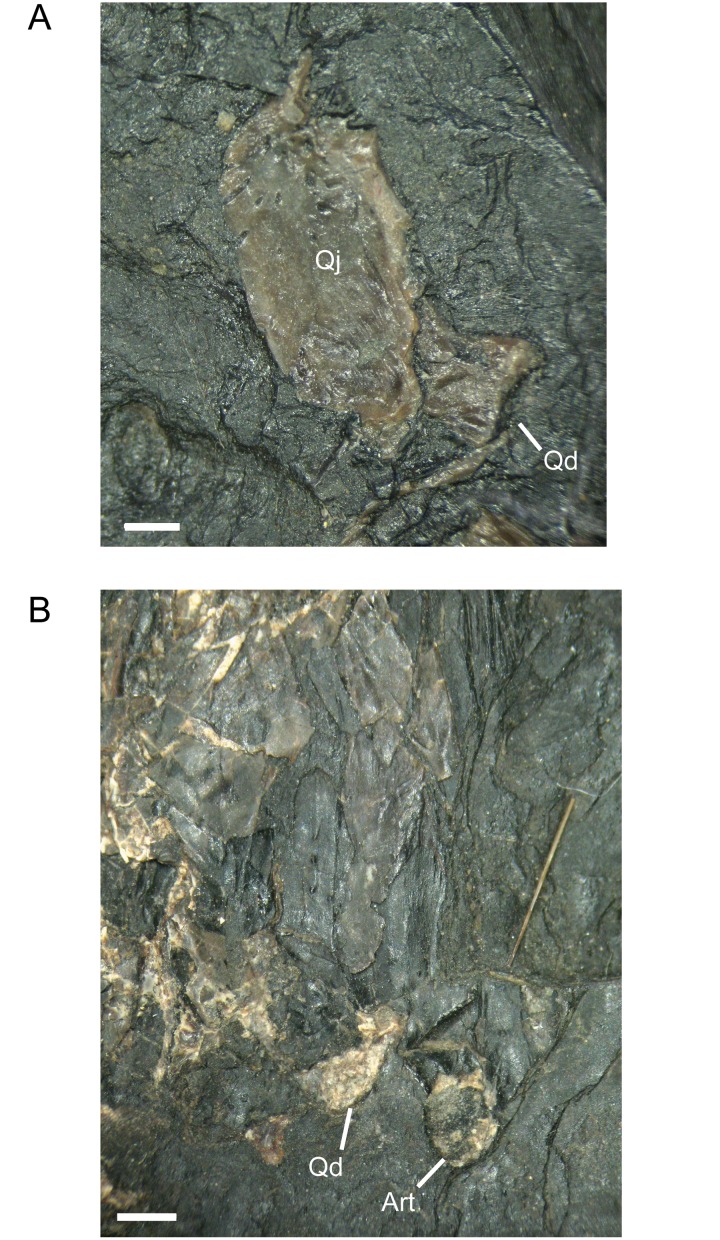
Jaw joint, *M*. *pelikani*. A. St.198, quadratojugal and quadrate, anterior up. B. CGH251, quadrate and articular, anterior up. Art, articular; Qd, quadrate; Qj, quadratojudal. Scale bars = 1mm.

In one specimen, MB.Am.821, the quadrate is in association with both the articular and the quadrate ramus of the pterygoid, although it is twisted out of position (Fig 10). As previously figured for a different specimen (MB.Am.838.2; [9] reference figure 4), the quadrate is robust and columnar in shape, not as squat and disproportionately large as reconstructed by Caroll and Gaskill ([1] reference figure 77D). The mandibular condyle is well-developed and appears convex or saddle-shaped for articulation with the lower jaw. On what I infer to be the anteromedial surface of the quadrate there is a shallow, oval depression which may be for contact with the stapes. In another specimen, St.198 (Narodini Museum, (now National Museum Prague), Prague, Czech Republic), the articular surfaces of both the quadrate and articular are visible, although the former is damaged (Fig 13B). It also appears as if that quadrate is articulated with the quadratojugal anteriorly, and the quadrate ramus of the pterygoid laterally. In CGH251, where the cheek elements are completely disarticulated, the quadratojugal covers much of the quadrate, although the columnar shape of the latter and its convex articulation surface for the articular are clearly visible (Fig 13A).

#### Lower Jaw

**Fig 14:**
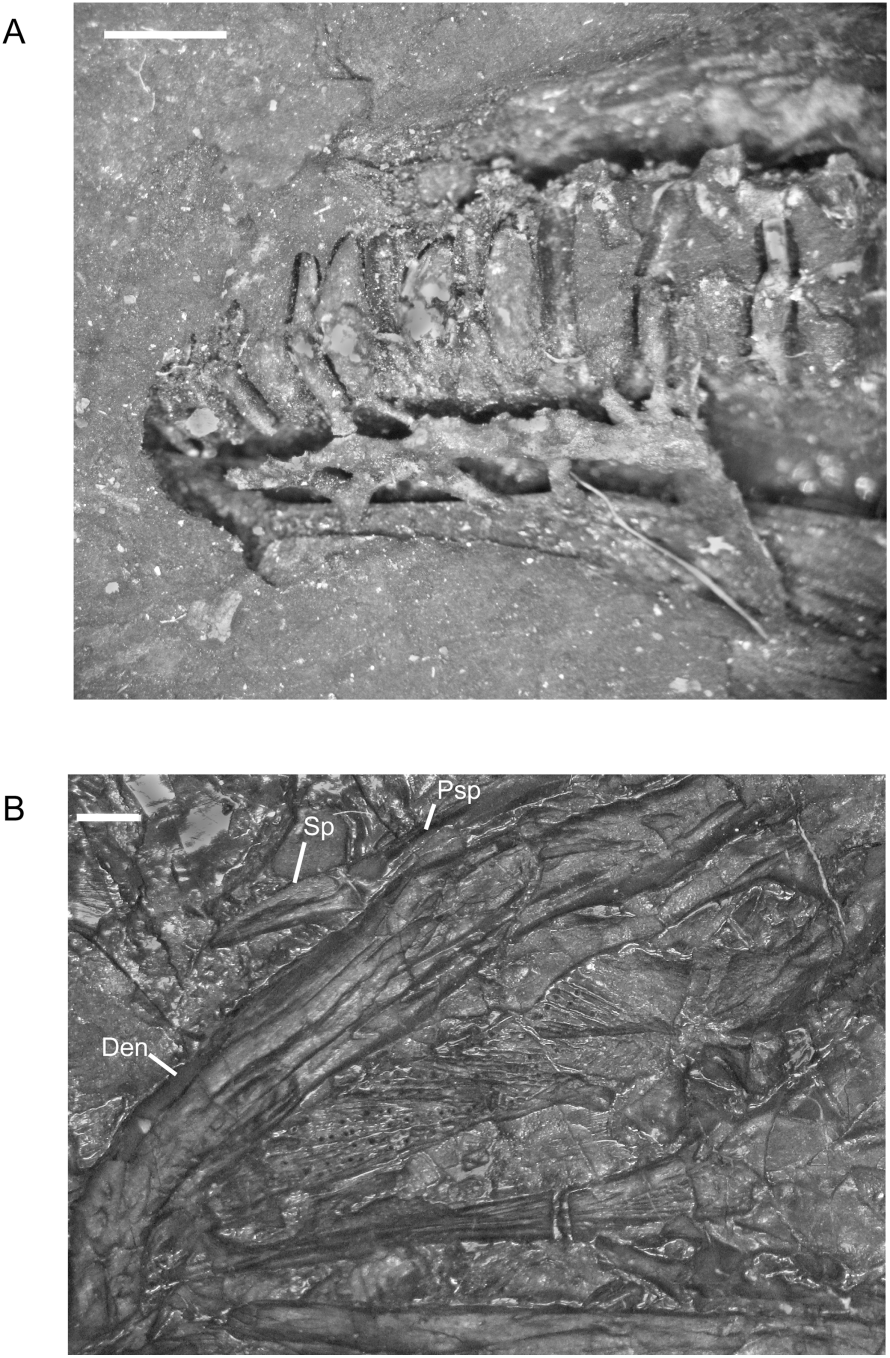
Lower jaw, *M*. *pelikani*. A. Sagittal section of mandible displaying natural cast of mandibular canal; NHMW1983_32_67; anterior to the left, dorsal up. B. Ventral view of palate and lower jaw; CGH727; anterior to the left, lateral up. Specimen is impression, but angle of light creates 3D effect. Den, dentary; Psp, postsplenial; Sp, splenial. Scale bars = 1 mm.

There are no significant ontogenetic changes in the morphology of the lower jaw. One specimen, NHMW1983_32_67, suffered damage to the lower jaw in such a manner as to reveal a natural cast of the mandibular canal and tooth roots (Fig 14A). In the endocast of the mandible, narrow passages connect the mandibular canal space not only to the tooth roots, but also to lateral openings in the dentary, which were probably innervated and possibly part of the lateral line system. The range in the maximum number of dentary teeth that I recorded for *M. pelikani* is 22–31, an increase from the maximum of 29 reported by Carroll and Gaskill [1]. Similar to the pattern observed in the maxilla, the middle teeth of the dentary, usually teeth 13–21, are largest.

Although the splenial was described previously, specimen CGH727 provides a more complete view of this element. The mandible is crushed under the skull and the splenial is partially disarticulated from the dentary and postsplenial (Fig 14B). Despite a relatively narrow lateral exposure when in articulation, the isolated splenial is triangular, exhibiting a wide base at the contact with the postsplenial and tapering to a point anteriorly. The splenial also is rotated slightly so that the dorsolateral surface is in view, revealing a narrow fossa that leads into the space within the bone.

#### Ribs and Vertebrae

**Fig 15:**
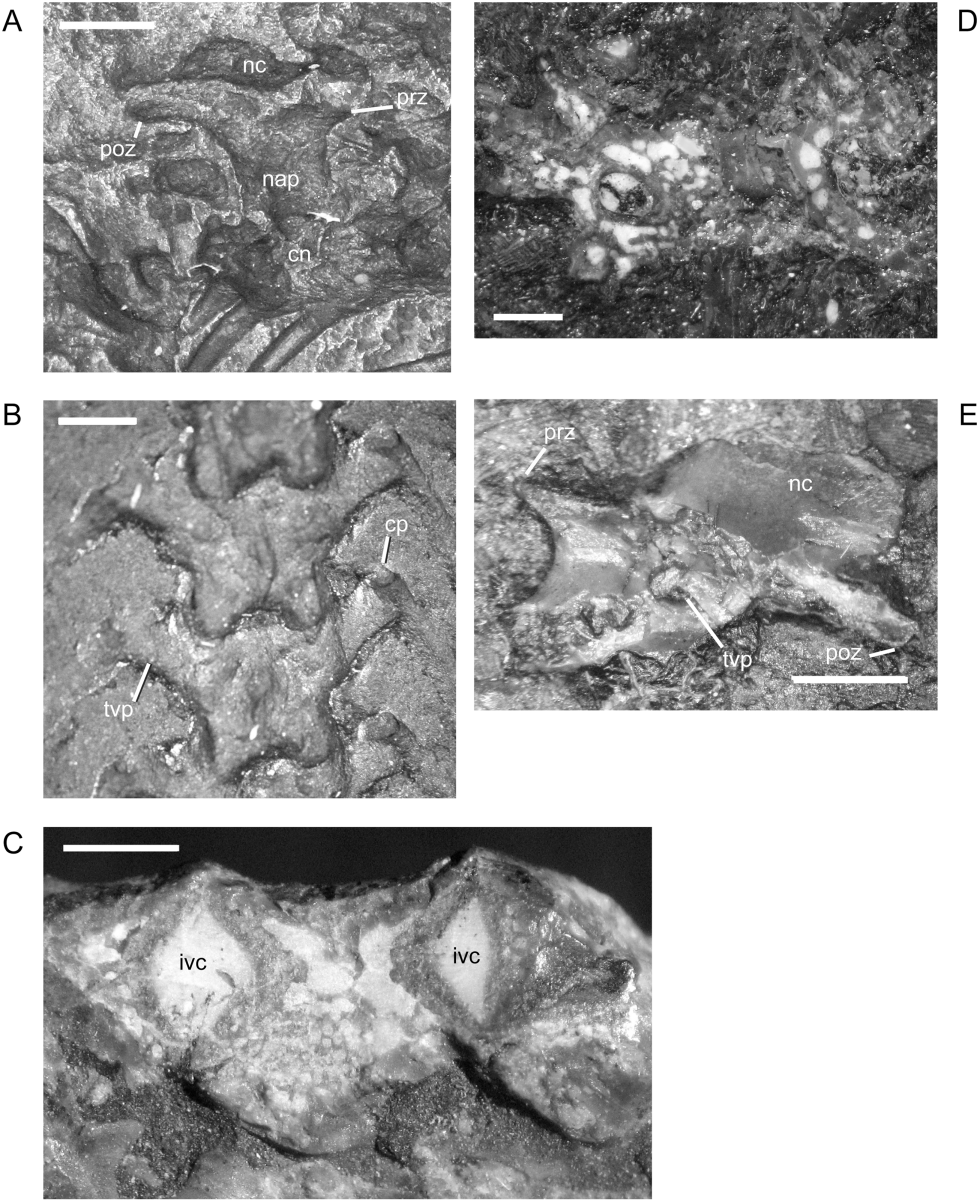
Vertebral elements, *M*. *pelikani*. A. St.193, vertebra showing tall neural arch pedicel; anterior to the right, dorsal up. B. AMNH2557 (American Museum of Natural History, New York, New York), dorsal view of neural arch showing anterolaterally directed transverse processes; anterior up. C. M3322, sagittal section through centrum; anterior to the left. D. St.207, anterior section through vertebrae. E. CGH256, medial view of neural arch half; anterior to left, dorsal up. Cn, centrum; Cp, capitulum of rib; Ivc, intervertebral cartilage region; Nap, neural arch pedicel; Nc, neural crest; Poz, postzygopophysis; Prz, prezygapophysis; Tvp, transverse process. Scale bars = 1mm.

No uncinate processes were present on the ribs of any *M. pelikani*, contra reports by Carroll and Gaskill [1]; prior descriptions are otherwise accurate. Changes over ontogeny were not observed, but in most cases the heads of the ribs were obscured either by matrix or their articulation with the vertebrae. Nearly all complete specimens of *M. pelikani* that I examined possess 38 presacral vertebrae. Only four specimens show variation by possession of 39 presacral vertebrae, although counts were difficult in those individuals. The large number of vertebrae is diagnostic for *M. pelikani* relative to other ‘microsaurian’ lepospondyls present in Nýřany, such as *H. longicostatum* (see below), *Sparodus*, *Ricnodon*, and *Crinodon*. The angle of the incline of the neural arch is usually between 5 and 15 degrees, although it may reach 20 degrees in some individuals. In smaller specimens, the arches are relatively high, with a narrow neural arch pedicel (Fig 15A). The base is relatively wider in larger specimens, but the pedicels do not exceed the anterior two-thirds of the centrum in length. The location of the contact was mistakenly described as on the posterior portion of the centrum, but was figured accurately by Carroll and Gaskill ([1] reference figure 81A,D). The transverse processes of *M. pelikani* are deflected anterolaterally (Fig 15B), as in most other ‘microsaurian’ lepospondyls [1], but unlike the condition in many other tetrapods.

As moderately supported by traditional morphometrics [17], the proportions of the centrum change during ontogeny. In small, hypothetically younger individuals, the centra are more angular, resembling a bow-tie rather than the smoothly curved, spool-shaped elements present in larger specimens. Visually it appears that relative centrum width decreases during growth, but that observation is supported only when compared to skull length, not centrum length [17]. Additionally, the amount of constriction at the middle of the centrum increases with growth.

In a large number of specimens the centra were sheared in half longitudinally to reveal the internal composition (Fig 15C). The outer walls of the centrum are composed of a spongy, dark bone that is suggestive of endochondral origins. The space between cotyles may have been occupied by unossified intervertebral cartilage; it is preserved as a much lighter, smoother material that lacks the spongy appearance associated with calcified cartilage. The center of the centrum is not hollow. Instead, both cotyles and the notochordal restriction were filled with a combination of cartilage and endochondral bone (or calcified cartilage). Vertebrae sheared in anterior or posterior view display large pockets of what may have been unossified cartilage within the endochondral bone of the neural arch, centrum, and transverse processes (Fig 15D).

One aspect of vertebral growth that received much attention in *M*. *pelikani* is the nature of the arch-centrum suture. The arch-centrum suture can be assessed only *in situ* in specimens that preserve vertebrae in lateral view. Although this is not an uncommon situation, it does reduce the available sample size. An unambiguous suture is present between associated neural arches and centra in three specimens with skull lengths ranging from 17–20mm. Not surprisingly, the majority of the *M*. *pelikani* specimens are preserved with some level of disarticulation and it is possible to infer the presence of an arch-centrum suture based on the shape of the disarticulated arches and centra. There is a consistent and repeated pattern to the shape of the ventral margin of the neural arches and the dorsal margin of the centra that includes the area around the transverse process, which always remains with the neural arch (Fig 15E). The pattern suggests that the arches are easily separated from the centra and were not forcibly broken off during deposition. In only four specimens do the arches appear to have actually been broken from the centra, resulting in a jagged line of separation that may leave the transverse process on the centrum fragment in some cases, and the arch fragment in others. All four of those specimens have skull lengths larger than 20mm, which is at the upper end of the size distribution.

The atlas neural arch was described as paired by Carroll and Gaskill [1] and unpaired by Vallin and Laurin [9], but the arches of the remainder of trunk vertebrae were reported to be unpaired [1]. However, Carroll and Gaskill [1] were inconsistent regarding the latter remark because in the same study they reported that “there is a tendency for the two halves of the neural arches in the trunk region to separate easily” (p. 123), implying that the arches are paired. Although data from the majority of specimens are inconclusive, I agree that in many individuals the arch halves readily disarticulate from one another. That observation supports the interpretation that all neural arches are paired and the arch halves are united by a suture rather than fusion, at least in small and medium individuals. One large specimen with a skull length of 26mm, shows evidence of fusion, suggesting that later in ontogeny the neural arch halves are unpaired.

Haemal arches are preserved in only 36% of the individuals that I examined, but presence is uncorrelated with size [16]. That pattern suggests that the haemal arches are easily lost either as a result of weak ossification or because they are not fused to the centra.

**Fig 16:**
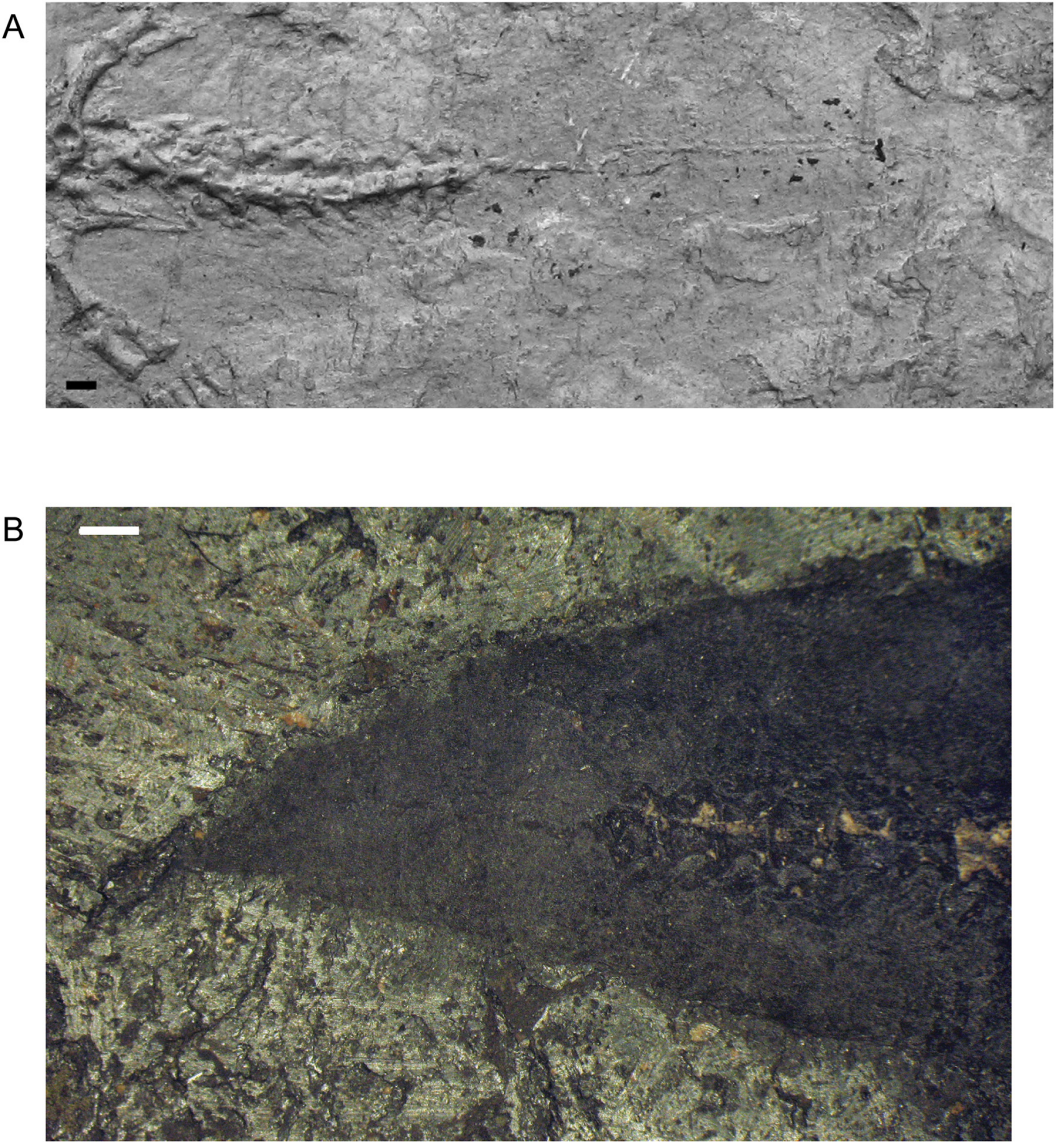
Tail regeneration in *M*. *pelikani*. A. MB.Am.815, showing regenerated tail segment; anterior to the left; plaster cast. B. NHMW1894-2332, showing posterior tip of tail in bone and outlined as skin impression; anterior to the right. Note relatively large size of terminal vertebrae. Scale bars = 1mm.

An even less common feature of the caudal vertebrae in *M*. *pelikani* is evidence of tail regeneration. In one specimen, the normal, fully developed caudal vertebrae are abruptly interrupted at about the 11^th^ caudal to give way posteriorly to much smaller, amorphous blocks of bone (Fig 16A). The transition in morphology is not gradual, but instead step-like, and occurs relatively close to the sacral region, considering that specimens of *M*. *pelikani* possess as many as 45 caudal vertebrae ([15]; pers.obs.). Immediately posterior to the abrupt transition, the anterior neomorphic vertebrae have a single row of elements that probably represent the centra. The regenerated vertebrae located further posteriorly, however, exhibit two rows of elements, likely the neural arches and centra. Carroll and Gaskill ([1] reference figure 81F) illustrated the same pattern for an additional specimen of *M*. *pelikani* that I did not examine. In non-regenerated tails, the caudal vertebrae remain relatively large posteriorly, even as they approach the soft-tissue tip of the tail (Fig 16B).

#### Pectoral Girdle and Forelimb

**Fig 17:**
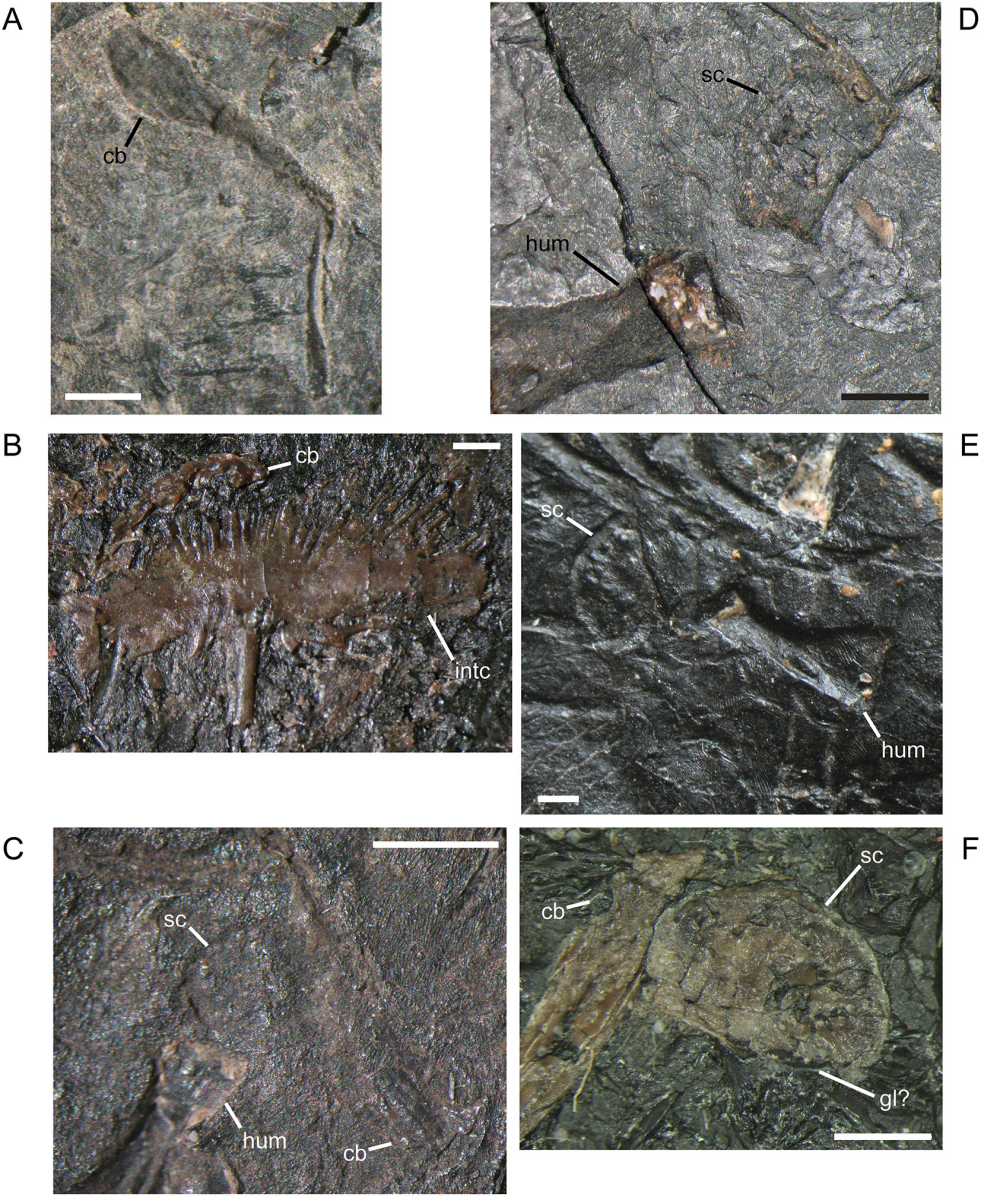
Pectoral girdle of *M*. *pelikani*. A. M1681, clavicle; ventral view, bone/impression. B. CGH142, interclavicle; ventral view, anterior up, bone. C. St.198, scapula; ventral view, anterior up, medial left, bone/impression. D. NHMW1983_32_49a, scapula; ventral view, anterior up, bone/impression. E. MB.Am.17, scapula; ventral view, anterior to the left, medial up, impression. F. CGH256, scapula; bone. Cb, clavicle blade; Gl?, glenoid fossa?; Hum, humerus; Intc, interclavicle; Sc, scapula. Scale bars = 1mm.

The dermal elements of the pectoral girdle, cleithrum, clavicle, and interclavicle, ossify early [16] and are relatively unchanged during growth. In contrast to a previous description, but in agreement with an illustration from the same study ([1] reference figure 120G), the clavicle has a relatively broad, paddle-like blade with a lightly sculptured or roughened ventral surface (Fig 17A). The width of blade that is visible depends on the orientation of the clavicle as preserved. The interclavicle of *M. pelikani* has distinct fimbriations anteriorly, although that morphology is also present in *H. longicostatum* and the lepospondyl *Sparodus validens*, also from Nýřany ([1]; pers.obs.). In *M. pelikani*, the fimbriations are elongate and gracile (Fig 17B), and are present in the smallest individual possessing an interclavicle, which is not surprising considering that the bone is not pre-formed in cartilage. In all specimens examined, the length of the interclavicle stem is approximately one-third the width of the interclavicle plate. A cluster of small, round foramina or pits commonly is located dorsal to the interclavicle stem. In larger individuals, the posterior processes flanking the interclavicle stem may elaborate into long curved or bifurcate structures (Fig 17B).

In *M*. *pelikani*, the scapula ossifies late relative to the rest of the postcranial elements [16]. The three smallest specimens that clearly possess an ossified scapula all have a skull length of 17mm. In those individuals, the element is relatively small and has an irregular ‘D’ or subcircular shape (Fig 17C). In larger specimens, the D-shape becomes even more pronounced and the scapula exhibits a robust, thickened ridge around its curved edge (Fig 17D). The glenoid, however, is still not well developed, although its location is visible. One of the largest individuals, MB.Am.17 (same specimen as MB.Am.822), was figured by Carroll and Gaskill ([1] reference figure 82A, as MB 1898.67), although only part of the endochondral girdle was actually illustrated. There is a flaw in the cast, but observation of the original specimen shows the true size and shape of what is either one large scapula with a crack at the center, or a scapula plus coracoid (Fig 17E). There is continuous ornamentation or pitting located along the curved edges of the two halves and together the pieces contribute to the glenoid, surrounding the proximal humerus, which demonstrates that the pieces form one functional unit, whether scapula or scapula-coracoid. Another of the largest individuals (CGH256; based on 3mm centrum length) may possess only the portion of the coracoid that immediately contributes to the glenoid, although the bone in that area is poorly ossified, leading to some doubt (Fig 17F). The ‘scapula’ and ‘cleithrum’ figured and described by Steen ([46] reference figure 19E) do not conform with the morphology observed in other specimens and were probably misidentified.

**Fig 18:**
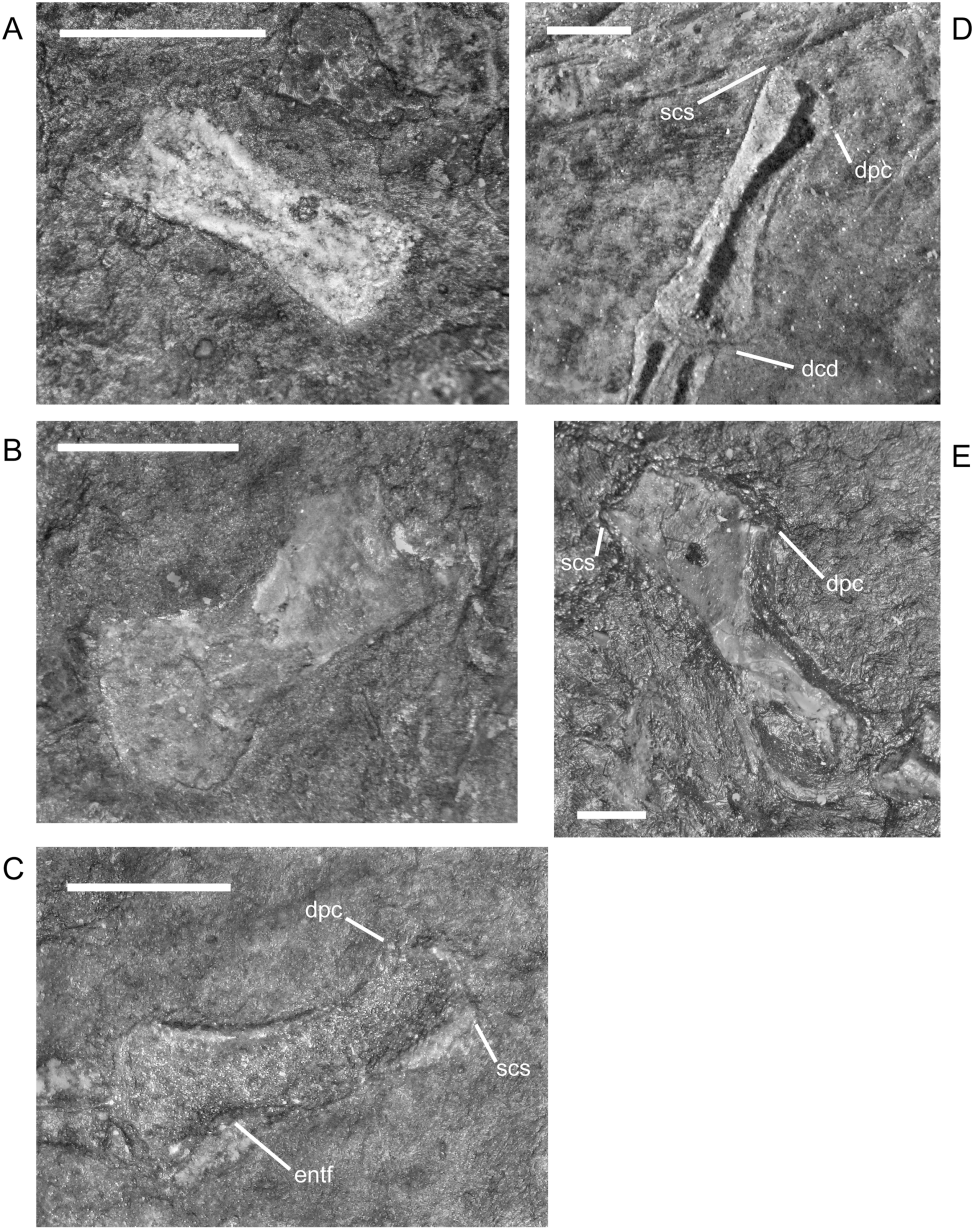
Ontogenetic changes in the humerus of *M*. *pelikani*. A. Stage 1, NHMW1894-2400; proximal toward top left. B. Stage 2. NHMW1983_32_52; proximal toward top right. C. Stage 3, NHMW1898_X_33; proximal toward top right, ventral view. D. Stage 4, MB.Am.810; proximal toward top right, dorsal view. D. Stage 5, NHMW1894_2399; proximal toward top left, dorsal view; note 90° torsion. Dcd, distal condyles; Dpc, deltopectoral crest; Entf, entepicondylar foramen; Scs, subcoracoscapularis attachment point. Scale bars = 1mm.

The entepicondylar foramen of the humerus, which is a prominent, large, oval slit in *M*. *pelikani*, is visible only in ventral (flexor) view. It is present in individuals of all sizes when the humerus is preserved in the appropriate orientation (Table 1). In the smallest specimens, the humerus is a simple, straight column of bone with an unwaisted shaft, unfinished ends with flat surfaces, and no processes (Fig 18A). At this stage of development, there also is no torsion. In the next stage of morphogenesis, the shaft of the humerus becomes distinct from the proximal and distal ends, which are more rounded than in the less developed humeri (Fig 18B). Later, the subcoracoscapularis attachment point begins to form slightly ahead of the deltopectoral crest, and a low degree of torsion (~30–45°) is present between the distal and proximal ends. During early stages of development of the deltopectoral crest, the structure is small, rounded, and located close to the proximal head (Fig 18C). Next, the humerus exhibits preliminary differentiation of the distal condyles (Fig 18D). In smaller individuals, differentiation of the condyles begins as an asymmetric shape-change of the distal shaft, which extends further on the ectepicondylar (radial) side. Torsion may reach 90° in some specimens at this point of development, although others show less twisting. During the next stage of morphogenesis, the deltopectoral crest becomes more prominent, assuming an angular appearance, and is shifted distally, whereas the distal condyles become even more distinct, often via development of a shallow fossa in between the condyles (Fig 18E). Once the condyles and processes are fully developed, torsion is almost always 90°. Throughout the growth of the humerus, the proximal head becomes progressively more round (convex). Sequence variation exists and many specimens have not yet developed structures that are clearly visible in smaller specimens. In other words, those larger individuals are at an earlier stage of morphogenetic development [16]. Additionally, one specimen shows evidence of left-right asymmetry in the degree of development of the humerus, although some of the disparity is likely caused by deformation and a thick covering of scales.

**Table 1:** Features of the humerus during ontogeny in *M*. *pelikani*.

specimen	Sl	Tl	view	Dpc	Scs	Dcd	torsion (deg)	Entf	shaft waisted
NHMW1983_32_66	7	.	dor	N	N	N	0		Y-small
MB.Am.809	.	45	ven	N	N	N	0	Y	N
CGH267	8.2	.	post	N	S	N	30		Y
St.190	9.4	46	dor	N	N	N	0		Y
NHMW1894_2400	.	46	dor?	N	N	N	0		N-small
NHMW1983_32_59	.	47+		S,P	S	N	0		Y
NHMW1898_x_29	10	54	dor?	N	N	N	0		Y
St.193	11	52	dor	N	B	pDiff?	0–30		Y
NHMW1983_32_50&52	13	58?	dor or ven?	N	N	N	0		Y
NHMW1898_X_33	13	68?	ven and dor	S,P	S	N	0	Y	Y
St.204	13.2	53	?	?	S?	N	?		Y
MB.Am.821.1–2	14	65	ven	S,P	S	N	0	Y	Y
M1381	14	65	?	B	B	N	60		Y
NHMW1899_III_8	14.5	.	ant or post	S,P	S	?	?		Y
MB.Am.827	14+	.	ant	S,P	S	?	70–90		Y
St.199	15	67	ven	N?	N?	N?	0	Y	Y
St.208	15	.	dor	N?	S	pDiff	0–30		Y
NHMW1898_X_30	15	70?	ven and ant	S,P	S	pDiff	30	Y	Y
M1700	15.5	71	post or ant	S,P	S	pDiff	30		Y
NHMW1983_32_64&74	15.7	.	ven and ant	S,P	S	pDiff	30	Y	Y
CGH139	15+	71	ant	S,P	L	?	?		Y
M1681	15+	80	post	L,D	L	fDiff	70–90		Y
MB.Am.811	16	76+	vent to post	S,P	S	pDiff	70–90	Y	Y
M1686	16.5	.	ant or post	S,P	S	?	70–90		Y
NHMW1983_32_72	17	.	ven or dor	B	B	pDiff?	90		Y
MB.Am.825.1–3	17	.	dor to ant	L,P	L	fDiff	90		Y
M4883	17	74	ven to post	S,D	S	pDiff	60	Y	Y
MB.Am.823	17	71+	ven	B	?	pDiff	80	Y?	Y
CGH727	17	.	ant	S,D	L	B	90		Y
MB.Am.810.1–2	17	80	ven	S,P	S	pDiff	45	Y	Y
Amnh2557	17.5	69	ant to ven	S,D	S	fDiff	70–80		Y
M1694	17+	79	ant or post	L,D	L	?	?		Y
MB.Am.814	18	.	post	S,P	L	pDiff	60		Y
M 4884	19	.	post	S,D	S	?	90		Y
NHMW1894_2332	19	77.5	ventral	?	?	pDiff	90		Y
NHMW1983_32_49ab	19?	79.5	ven	B	?	N	90	Y	Y
St.198	19	.	ven and post	L, D	L	fDiff?	90		Y
CGH34	19.5	.	ant or post	L, D	L	B	90		Y
CGH254	20?	73	ant	L,D	L	fDiff	80–90		Y
M3321-3322	20.1	.	ven	B,D	B	fDiff?	90	Y	Y
CGH2098	22	82	post to ven	L,P	L	fDiff	60–70		Y
NHMW1894-2364	22	.	post to ven	L,B	?	pDiff	60–70	Y	Y
M1688	25	.	ant or post	L,D	L	fDiff	90		Y
CGH251	26	.	ven to post	B,D	L	fDiff	90	Y	Y
M4886	26	115	post	L,D	L	fDiff	90		Y
NHMW1983_32_67	27.5	.	ant or post	L, P?	L	B	90		Y
MB.Am.822.1 = MB.Am.17	28	.	ven	B	B	fDiff	90	Y	Y
MB.Am.838.1–3	29	.	vent	L,D	B	fDiff	90	Y	Y
NHMW1894-2399	.	.	post	L, D	L	fDiff?	90		Y
CGH256	.	.	ven	B	B	fDiff?	90	Y	Y
M1689	.	.	ven and post	L,D	L	fDiff	90	Y	Y
CGH142	.	.	ven	L, D	L	fDiff	B	Y	Y

Abbreviations: B, broken but present; D, distal, away from head; Dcd, distal condyles; deg, degrees; Dor, dorsal; Dpc, deltopectoral crest; Entf, entepicondylar foramen; fDiff, fully differentiated; L, large and prominent; N, not present; P, proximal, near head; pDiff, partially differentiated; S, small, not prominent; Scs, subcoraco-scapularis; Sl, skull length; Tl, trunk length; Ven, ventral; Y, present.

**Fig 19:**
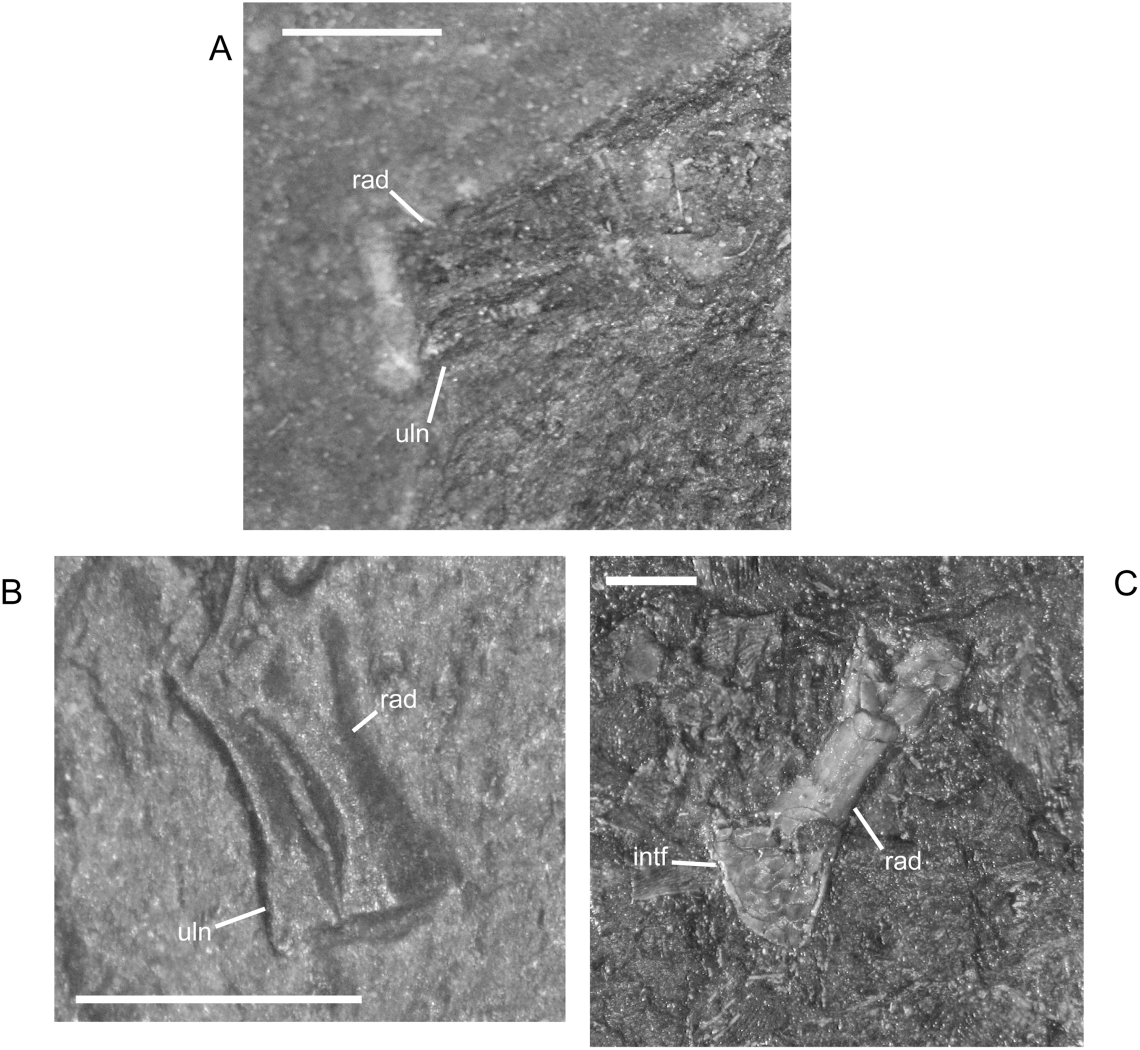
Ontogenetic changes in the radius of *M*. *pelikani*. A. Stage 1, NHMW1894-2504; proximal toward upper right. B. Stage 2, CGH267; proximal toward upper left. C. Stage 3, St.207; proximal toward upper right. Intf, intermedial facet; Rad, radius; Uln, ulna. Scale bars = 1 mm.

The ossified radius of *M*. *pelikani* first appears as a featureless, rectangular bone with no processes and flat, indistinct proximal and distal ends (Fig 19A). The first morphogenetic change to occur is the development of a distinctly waisted shaft with expanded proximal and distal ends (Fig 19B). Throughout ontogeny, the distal end of the radius is equal to or broader than the proximal end. The ends of the radius also become rounded (convex). During the next stage of morphogenesis, the medial surface of the distal end slants inward to form the intermedial facet (Fig 19C). The radiale facet is not well developed in any specimens, though St.207 and NHMW1894_2399 may show preliminary differentiation of that region.

**Fig 20:**
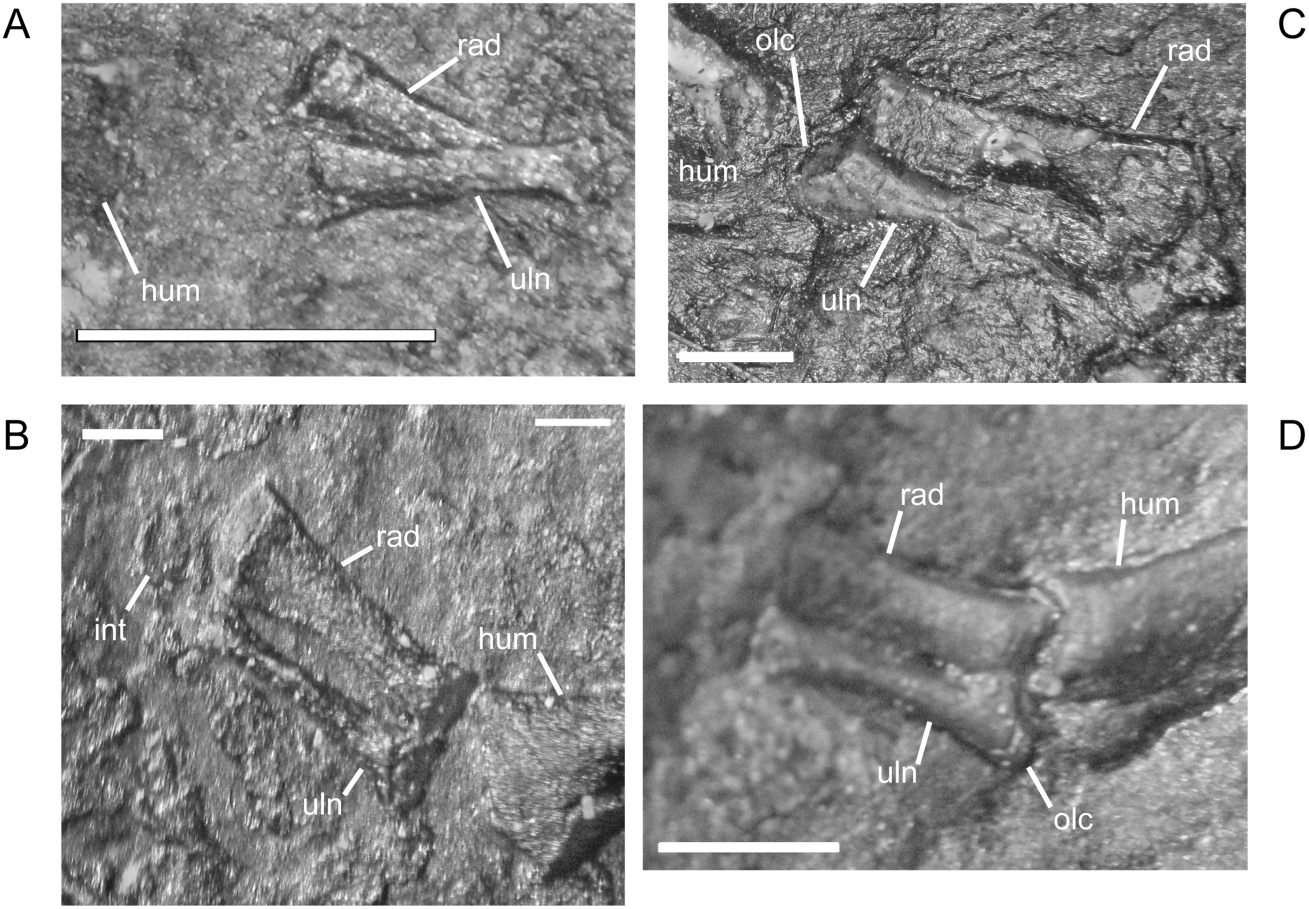
Ontogenetic changes in the ulna of *M*. *pelikani*. A. Stage 1, NHMW1983_32_66; proximal toward left. B. Stage 2, R.2814; proximal toward bottom right. C. Stage 3, NHMW1894-2399; proximal toward top left. D. Stage 4, AMNH2557; proximal toward bottom right. Hum, humerus; Int, intermedium; Olc, olecranon process; Rad, radius; Uln, ulna. Scale bars = 1mm.

Early in ontogeny, the ulna of *M*. *pelikani* also starts out undifferentiated, although the proximal end of the element is always at least slightly broader than the distal end (Fig 20A). As growth increases, the proximal end becomes much broader than the distal end (Fig 20B). Despite a contrary report [1], the olecranon process becomes ossified during ontogeny. Preliminary development of that process is manifest as a medial slant in the proximal surface of the ulna (Fig 20C). As the olecranon process becomes more developed, the rounded, high portion that articulates with the lateral surface of the humerus becomes distinct from the level surface of the proximal end of the ulna (Fig 20D). A well-developed intermedial facet does not appear to form in *M*. *pelikani*, but an ulnare facet may be partially developed in NHMW1894_2399, which also exhibits a partially developed olecranon process.

#### Pelvic Girdle and Hindlimb

**Fig 21:**
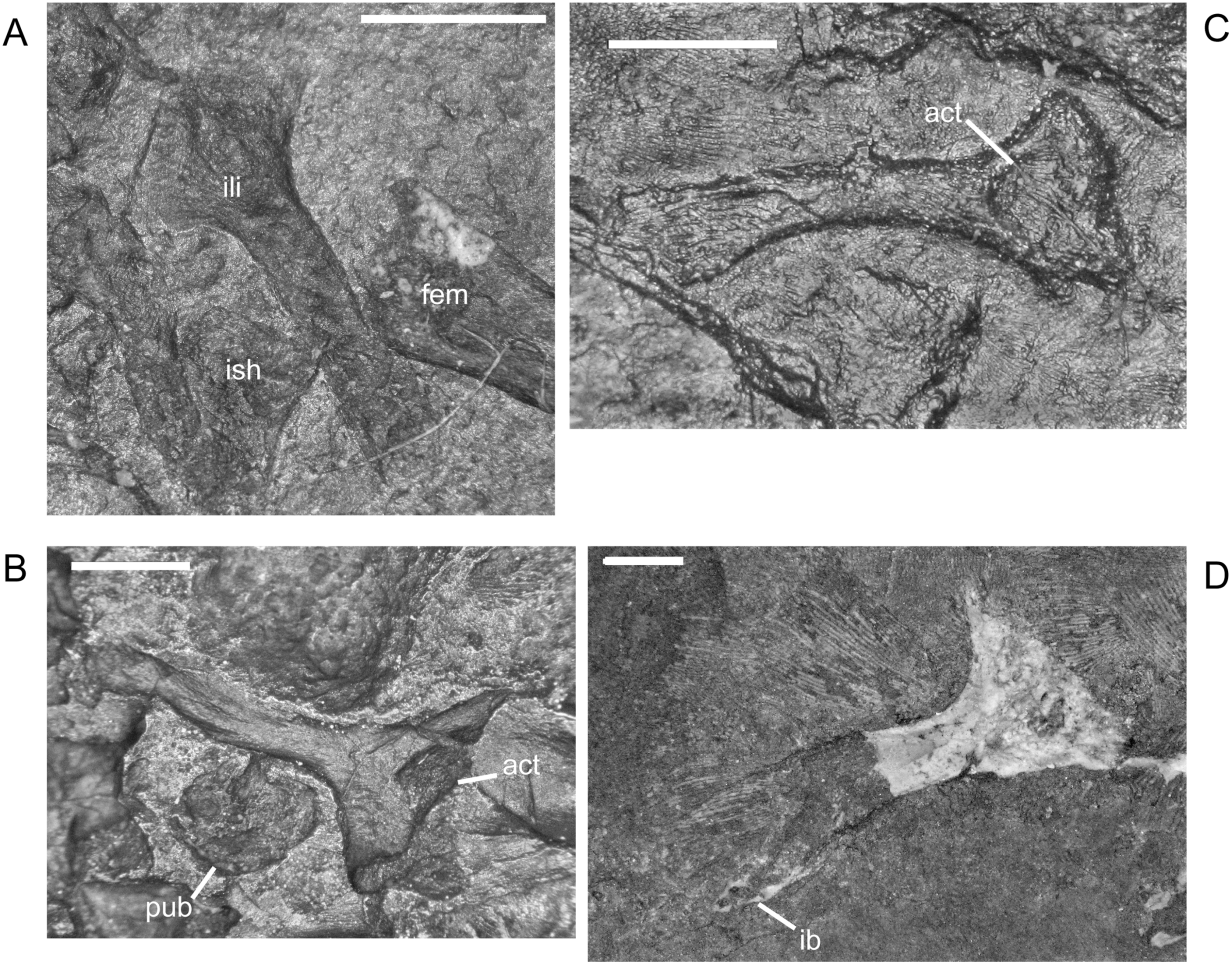
Ontogenetic changes in the ilium of *M*. *pelikani*. A. Stage 1, NHMW1894-2400; proximal toward top left. B. Stage 2, St.193; proximal toward bottom right. C. Stage 3, NHMW1983_32_3; proximal toward right. D. Stage 4, NHMW1894-2364; proximal toward top right. Act, acetabulum; Fem, femur; Ib, bifurcation; Ili, ilium; Ish, ishium; Pub, pubis. Scale bars = 1mm.

The ilium of *M. pelikani* forms relatively early in skeletal development [16]. At first, the ilium has a straight and relatively rectangular dorsal process (Fig 21A). The head is incompletely ossified and the acetabulum has not yet formed. The first major morphogenetic change is the development of the dorsal margin of the acetabulum along the ventrolateral margin of the iliac head (Fig 21B). In the next stage of development, the dorsal process of the ilium curves to assume a posterodorsal orientation at about the same time the acetabulum expands dorsally and becomes well-defined (Fig 21C). The posterior tip of the dorsal process is also less squared. Finally, during the latest stage of ilium development, the dorsal process bifurcates into two distinct, pointed processes and the head is greatly expanded for articulation with the rest of the pelvis (Fig 21D). However, even in the largest individuals, the ilium is not fused to the pubis, though it may be more firmly attached to the ischium.

**Fig 22:**
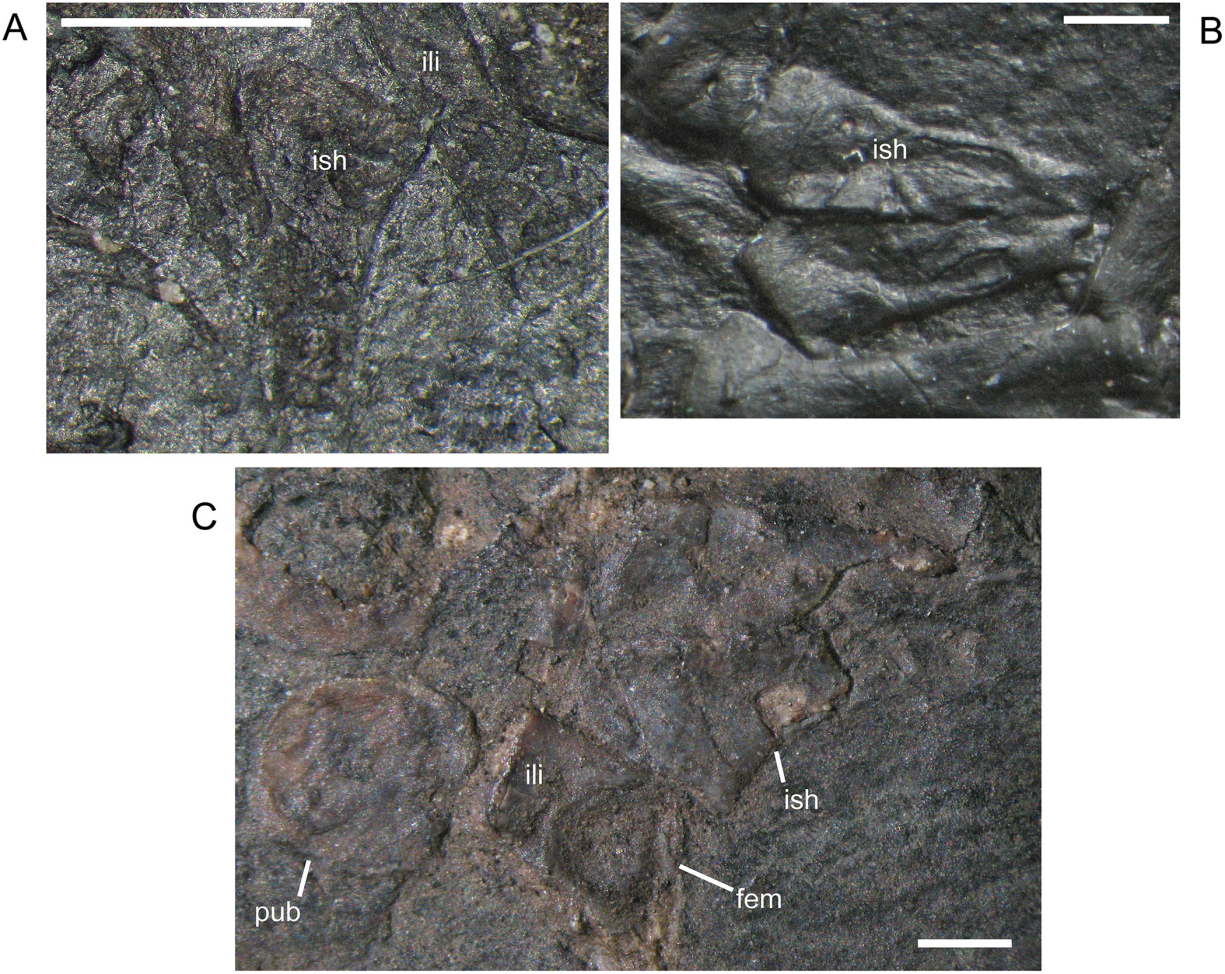
Ontogenetic changes in the ischium of *M*. *pelikani*. A. Stage 1, NHMW1894-2400; anterior is up, medial to the left. B. Stage 2, MB.Am.40; anterior is to the left; both sides present. Specimen is impression, but angle of light causes 3D effect. C. Stage 3, CGH2098, fully articulated pelvic girdle and femur; anterior to the left. Fem, femur; Ili, ilium; Ish, ishium; Pub, pubis. Scale bars = 1mm.

In *M*. *pelikani* the ischium ossifies after the ilium and before the pubis [16]. In the smallest specimens, the ischium resembles an isosceles triangle, with a bluntly squared posterior process (Fig 22A). With increased growth, the overall shape of the ischium becomes more asymmetric as the lateral edge broadens, but the medial margin becomes convex (Fig 22B). During the same stage of morphogenesis, the anterior margin of the ischium slants laterally and a longitudinal groove may be visible on the ventral surface of the element. As the asymmetry develops further, an angular process forms at the anteromedial corner of the ischium (Fig 22C). There is either an anteromedial foramen or a small, round depression in the ischium of a few specimens of *M*. *pelikani*, but it is unclear if the structure appears at a certain point in ontogeny or if it is only visible in certain views.

**Fig 23:**
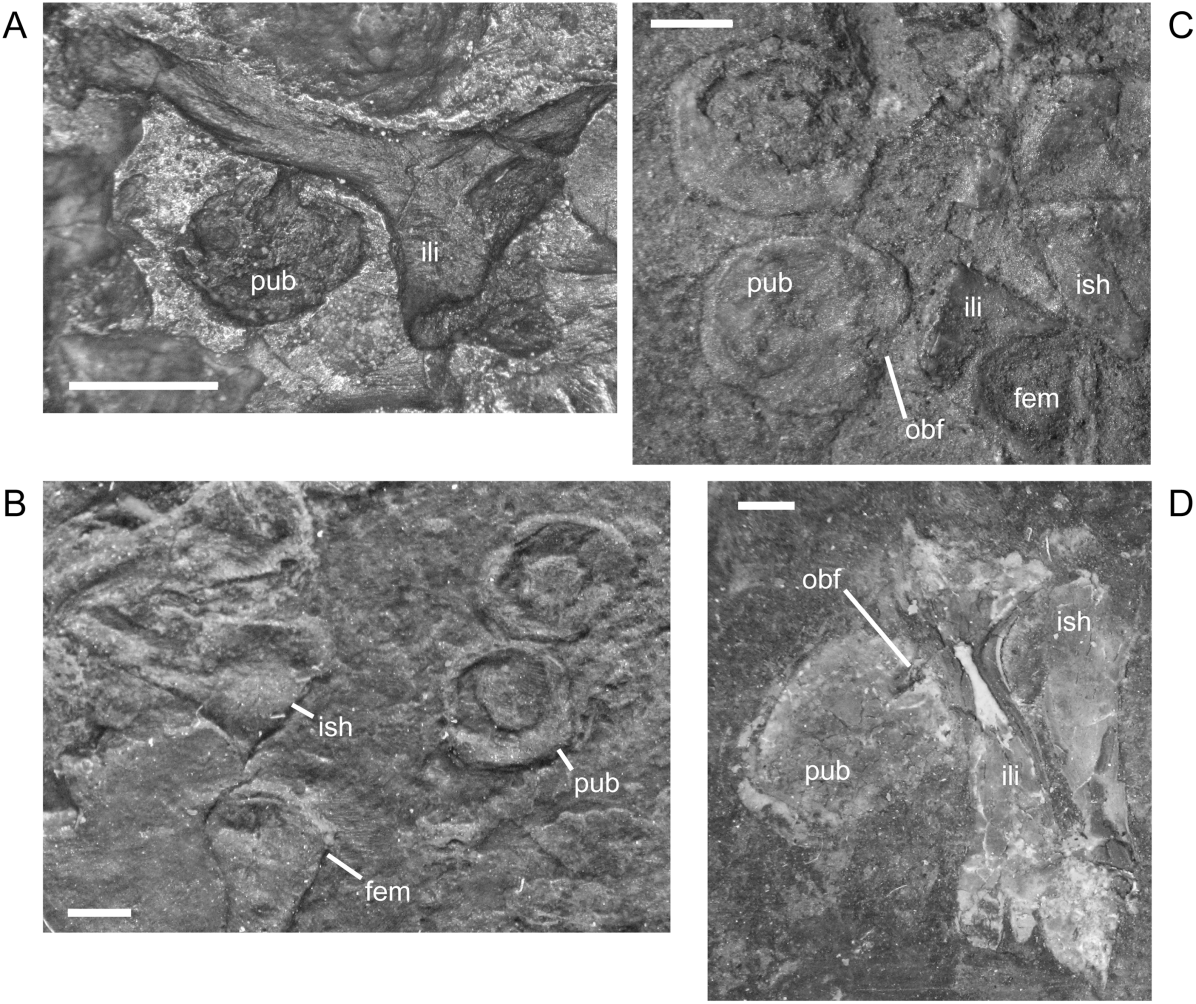
Ontogenetic changes in the pubis of *M*. *pelikani*. A. Stage 1, St.193; anterior up, medial to the right. Specimen is impression, but angle of lighting creates 3D effect. B. Stage 2, MB.Am.810; anterior to the right. Element from both sides present. C. Stage 3, CGH2098; anterior to the left. Element from both sides present. D. Stage 4, NHMW1983_32_67; isolated pelvis. Obturator foramen nearly closed. Fem, femur; Ili, ilium; Ish, ishium; Obf, obturator foramen; Pub, pubis. Scale bars = 1mm.

The pubis is one of the final elements to ossify during postcranial skeletal development of *M*. *pelikani* [16]. It first appears as an irregular, flat, subcircular bone (Fig 23A). Later in ontogeny a wide ring forms around the outer edge of the pubis, causing the center to appear depressed in small individuals (MB.Am.812-9). The thickened ring becomes proportionately narrower as the pubis increases in size and the center may instead be raised (Fig 23B). In more developmentally advanced specimens, the obturator foramen is first visible as a notch in the posteromedial margin of the pubis (Fig 23C). In one of the largest individuals, the pubis has nearly enclosed the obturator foramen (Fig 23D).

**Fig 24:**
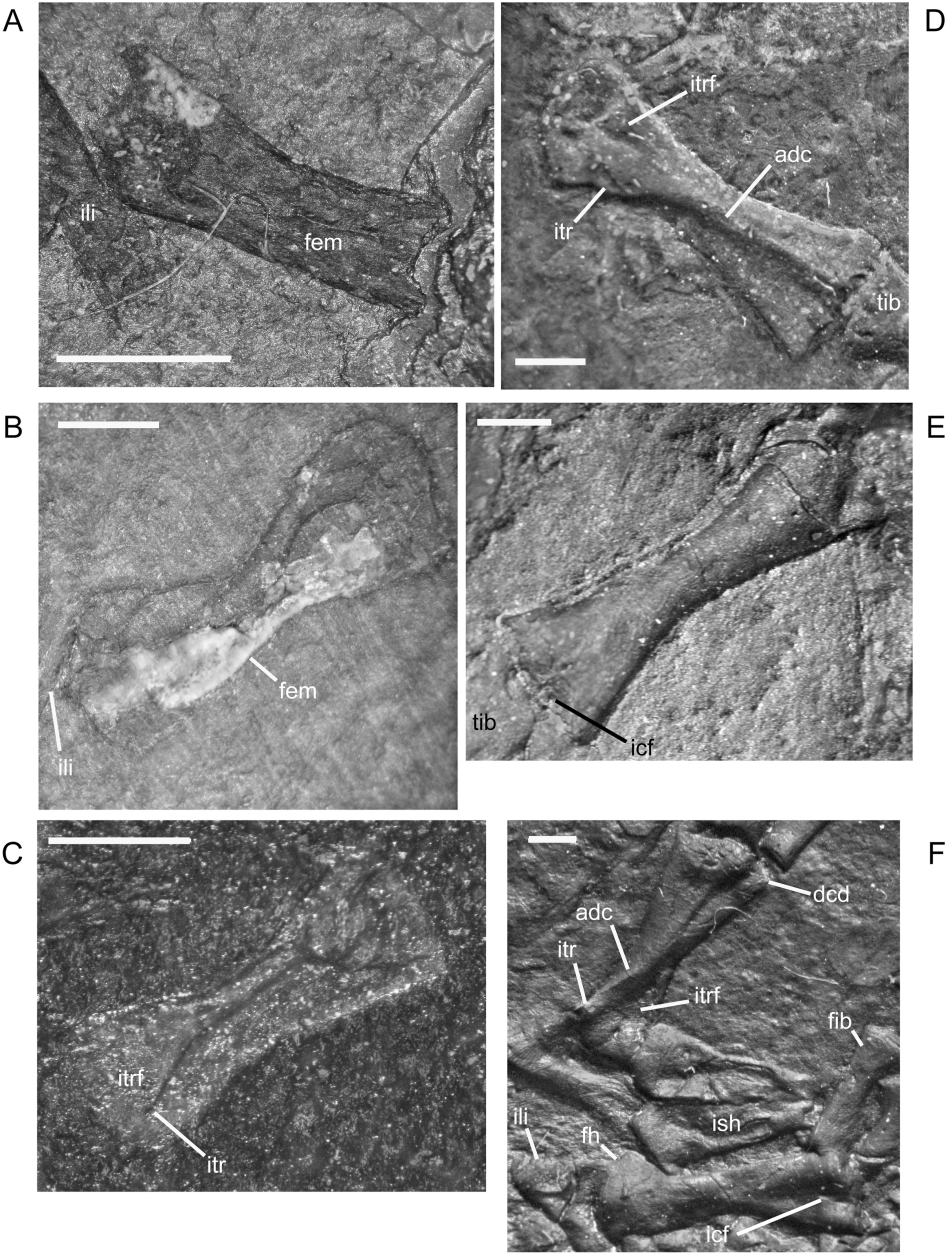
Ontogenetic changes in the femur of *M*. *pelikani*. A. Stage 1, NHMW1894-2400; proximal toward left. Note unfinished ends. B. Stage 2, NHMW1983_32_50; proximal toward bottom left. Note waisting of shaft. C. Stage 3, St.190; proximal toward bottom left. Impression of crushed element. D. Stage 4, MB.Am.810; proximal toward upper left. Internal trochanter is broken. E. Stage 5, AMNH2557; proximal toward upper right. F. Stage 6, MB.Am.840; Elements from both sides present, anterior is to the left. Femur toward top is ventral view, femur toward bottom is dorsal view. Adc, adductor crest; Dcd, distal condyles; Fem, femur; Fib, fibula; Fh, femoral head; Icf, intercondylar fossa; Ili, ilium; Ish, ischium; Itr, internal trochanter; Itrf, intertrochanteric fossa. Scale bars = 1mm.

The femur of the most immature specimens of *M*. *pelikani* is a simple rectangle of bone, with little waisting to distinguish the shaft, and incompletely ossified proximal and distal ends (Table 2). The intertrochanteric fossa may be developing in some specimens at that stage of morphogenesis, but in general, features are poorly developed (Fig 24A). During the next stage of development, the intertrochanteric fossa is identifiable and the shaft becomes distinct from the ends of the femur (Fig 24B). With increased growth, the ends of the femur become rounded, and a small internal trochanter and adductor crest appear (Fig 24C). The shaft of the femur is still narrowing and the distal condyles are not differentiated. In specimens preserved in dorsal view, presence of the internal trochanter is evidenced by medial slanting of the femoral head and a small, angular inflection at the proximal-medial corner of the femur. The next set of morphogenetic changes include notable strengthening of the adductor crest and preliminary differentiation of the distal condyles, often signified by the presence of a small fossa between the tibial and fibular facets (Fig 24D). At about the same time, or shortly after, specimens preserved in dorsal view exhibit a small intercondylar fossa at the distal end of the femur (Fig 24E). Additionally, the distal condyles are rounded, have separated from one another, and frequently exhibit distinct patches of bone for articular facets (Fig 24F).

**Table 2:** Features of the femur during ontogeny in *M*. *pelikani*.

	Sl	Tl	view	Itrf	Itr	Adc	Dcd	Icf	shaft waisted
NHMW1983_32_66	7	.	dor?	?	N	?	N	N	Y-little
MB.Am.809	.	45	Ven	?	B	S	N	?	Y
St.190	9.4	46	ven	Y	S	S	N	?	Y—little
NHMW1894_2400	.	46	ven?	N?	N	N	N	?	N
NHMW1898_x_29	10	54	?	?	N	?	?	?	N
St.193	11	52	ven	?	B	S	N	?	Y
NHMW1983_32_50&52	13	58?	ven	Y	N	N	N	?	Y
NHMW1898_X_33	13	68?	ven	Y	S	S	pDiff	?	Y
St.204	13.2	53	dor?	?	?	?	N	N	Y
M1381	14	65	ven	Y	B	S	N	?	Y
St.199	15	67	dor	?	B	?	N?	N	Y
MB.Am.833	15	64	ven	Y	S	L	N	?	Y
MB.Am.815.1–6	15	88	dor	?	S?	?	N	N	Y
CGH69	.	.	dor	?	S	?	N	N	Y
M639	16	92	ven	Y	S	L	pDiff	?	Y
MB.Am.810.1–2	17	80	ven	Y	S	L	pDiff	?	Y
M4883	17	74	dor	?	?	?	pDiff	S	Y
Amnh2557	17.5	69	dor	?	N?	?	pDiff	S	Y
St.198	19	.	ven	Y	B	N	fDiff	?	Y
NHMW1894_2332	19	77.5	ven	Y	L	S	fDiff	?	Y
NHMW1983_32_49ab	19?	79.5	veb	Y	B	L	fDiff?	?	Y
CGH254	20?	73	dor	?	?	?	N?	N	Y
MB.Am.812.1–2	20?	75?	ven	Y	S	L	fDiff?	?	Y
St.116	20+	120	dor	?	Y	?	fDiff?	?	Y
CGH2098	22	82	ven	Y	B	L	fDiff?	?	Y
CGH251	26	.	dor	?	Y	?	fDiff	fDiff	Y
M4886	26	115	dor	?	?	?	fDiff?	fDiff	Y
NHMW1983_32_67	27.5	.	dor	?	?	?	fDiff	?	Y
MB.Am.840.1–2	.	.	dor and ven	Y	L	L	fDiff	fDiff	Y

Adc, adductor crest; B, broken but present; D, distal, away from head; Dcd, distal condyles; Dor, dorsal; fDiff, fully differentiated; Icf, intercondylar fossa; Itr, internal trochanter; Itrf, intertrochanteric fossa; L, large and prominent; N, not present; P, proximal, near head; pDiff, partially differentiated; S, small, not prominent; Sl, skull length; Tl, trunk length; Ven, ventral; Y, present.

**Fig 25:**
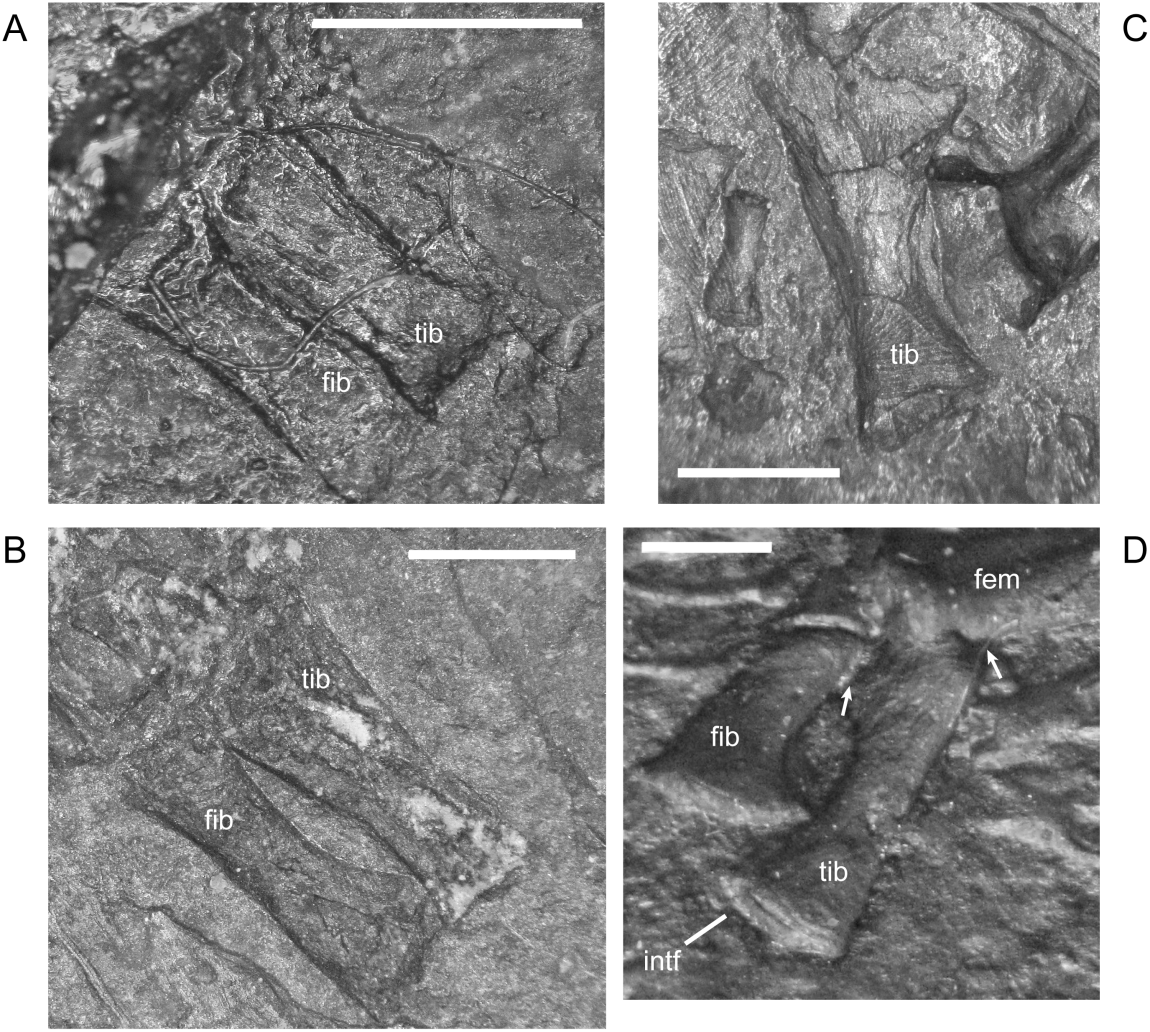
Ontogenetic changes in the tibia of *M*. *pelikani*. A. Stage 1, NHMW1898-2400; proximal toward upper left. B. Stage 2, NHMW1898_X_33; proximal toward upper left. C. Stage 3, St.193, proximal toward top. D. Stage 4, MB.Am.840; proximal toward top right. Proximal end of tibia partially crushed under femur; arrows denote base of proximal end. Intf, intermedial facet; Fem, femur; Fib, fibula; Tib, tibia. Scale bars = 1mm.

When initially ossified, the tibia of *M*. *pelikani* is a featureless column of bone (Fig 25A). Neither end of the tibia is greatly expanded at this stage, but the proximal end is barely wider than the distal end. During the next stage of morphogenesis, the proximal end is expanded much more than the distal end, leading to a size disparity that is maintained throughout ontogeny (Fig 25B). In contrast to a prior report by Carroll and Gaskill [1], the distal end is expanded relative to the tibial shaft, although the proximal end begins expansion before the distal end. Later in ontogeny, as a result of asymmetric narrowing of the tibial shaft, the tibia curves medially (Fig 25C). At about the same time, the head of the tibia slants medially. Finally, the distal end of the tibia slants medially to form the intermedial facet (Fig 25D). However, in many specimens with an ossified intermedium, the distal end of the tibia remains flat, although in a few individuals in which the tarsals are not developed yet, the facet is already present. Two specimens exhibit a tibial crest, but developmentally more advanced individuals seem to lack that structure. The ends of the tibia never become rounded and a distinct tibiale facet was not observed in any individual.

**Fig 26:**
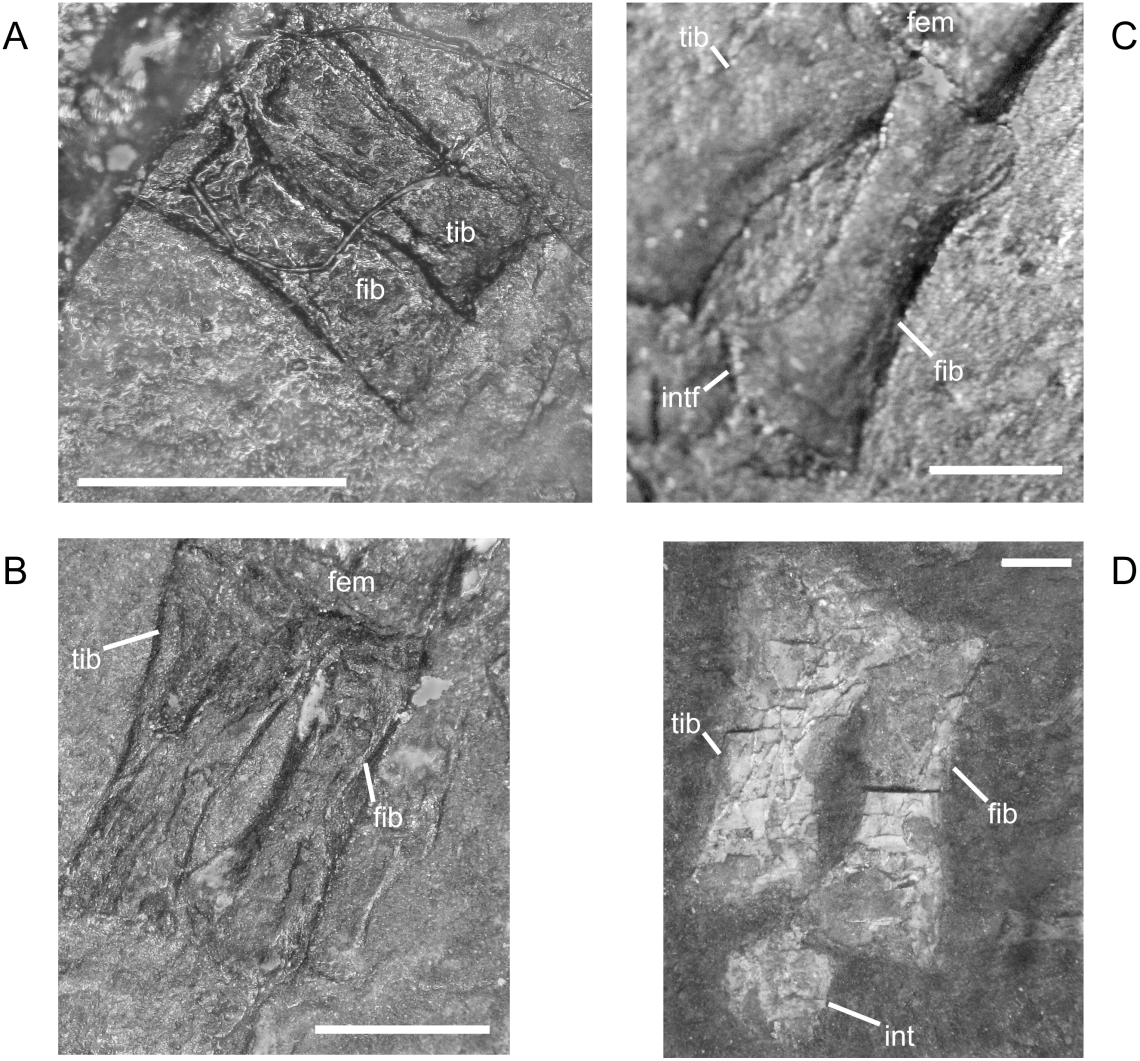
Ontogenetic changes in the fibula of *M*. *pelikani*. A. Stage 1, NHMW1898-2400; proximal toward upper left. B. Stage 2, NHMW1898_X_33; proximal toward upper right. C. Stage 3, AMNH2557; D. Stage 4, NHMW1983_32_67; proximal toward top. Fem, femur; Fib, fibula; Int, intermedium; Intf, intermedial facet; Tib, tibia. Scale bars = 1mm.

After initial ossification, the fibula and tibia nearly are indistinguishable. At that early point in ontogeny the ends of the fibula are approximately equal in breadth (Fig 26A). The first ontogenetic change is a marked expansion of the distal end of the fibula (Fig 26B). The expansion is asymmetric so that later in ontogeny the medial surface projects farther than the lateral surface. Shortly after expansion of the distal end, the fibula starts to appear curved, although only weakly. The intermedial facet, manifested as a medial slant in the distal end of the fibula, is the next feature to appear (Fig 26C). The facet is developed before ossification of the intermedium. In the most mature specimens, the fibula exhibits strong curvature, and both the proximal and distal ends are slanted (Fig 26D). I did not observe a distinct facet for the fibulare, although one specimen (CGH2098) may exhibit preliminary differentiation.

#### Epipodials, Metapodials, and Phalanges

**Fig 27:**
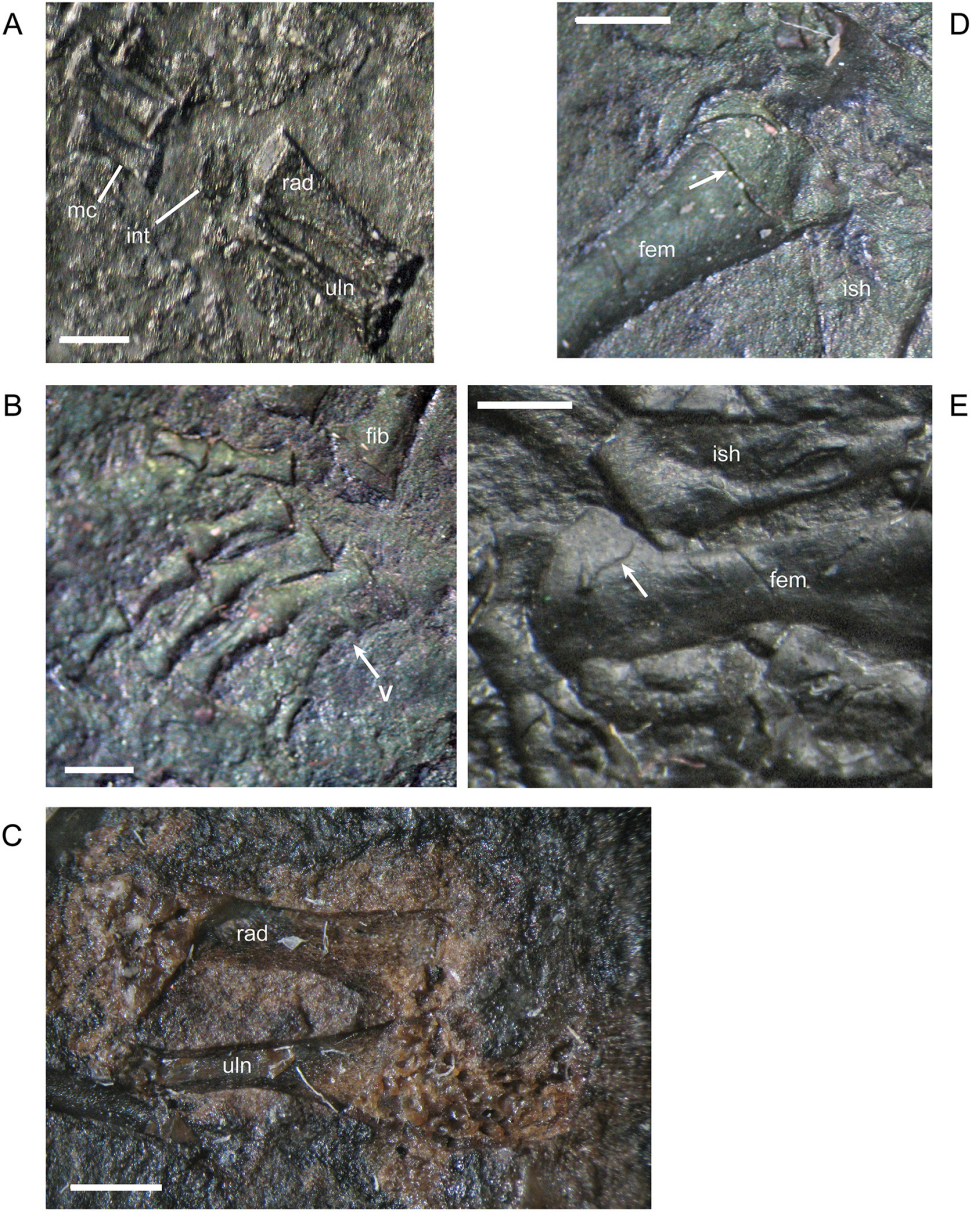
Carpals, digits, and potential epiphyses in *M*. *pelikani*. A. Ossified intermedium in the manus of R.2814; proximal toward bottom right. B. Pes of AMNH2557; proximal toward top right. Arrow points to poorly ossified digit five. C. Crushed radius and ulna of CGH142. Note profusion of cell spaces that may indicate epiphysis. D. Femur of AMNH2557; proximal toward top right. Arrow points to line between shaft and potential epiphysis. D. Femur of MB.Am.840; proximal toward the left. Arrow points to line between shaft and potential epiphysis of well-developed femoral head. Fem, femur; Fib, fibula; Int, intermedium; Ish, ischium; Mc, metacarpals; Rad, radius; Uln, ulna; V, digit five. Scale bars = 1mm.

In *M. pelikani*, a maximum of five ossified carpals was reported by Carroll and Gaskill [1]. The intermedium is the first carpal to ossify (Fig 27A). Based on one specimen (MB.Am.17) that possesses two carpals, the ulnare may be the second to ossify. However, precise identification is impossible because the wrist and partial digits are offset from the long bones. Specimen CGH3018, which exhibits five carpals, was reported to preserve the ulnare, intermedium, radiale, and two distal carpals [1]. In what appears to be ventral view, the carpals in *M. pelikani* have a small depression at the center of ossification.

Previously, only a single tarsal, the intermedium, was reported for *M*. *pelikani*. However, I observed three tarsals in specimen St116, although with the exception of the intermedium, the tarsals are poorly ossified. The two additional tarsals appear to be the fibulare and a distal tarsal. Unfortunately, the foot has rotated from the tibia and fibula making exact determination difficult. There may also be a few other, smaller, distal tarsals on the medial side of the foot, but poor preservation precludes firm identification of those structures as epipodials. There appears to be a depression at the center of the better preserved tarsals. Many other specimens exhibit only an ossified intermedium, suggesting that it is the first tarsal to ossify in *M*. *pelikani*, followed perhaps by the fibulare.

Digits are ossified in specimens of all sizes. Mirroring the long bones, in the least mature specimens of *M*. *pelikani*, the individual metapodials and phalanges are little columns of bone with no distinct shape. However, the distal-most phalanges are fully-formed as triangular, slightly curved, sharply pointed elements. That morphology is also exhibited by specimens of *H*. *longicostatum* (see below). During the next stage of morphogenesis, all phalanges elongate and the proximal and distal articular surfaces expand. Later, the ends of the metapodials and phalanges are more rounded. In the articulated foot of many specimens, digit 5 is often less well-developed than the rest of the digits, suggesting that it forms last (Fig 27B).

#### Epiphyses

The long bones of *M. pelikani* were not reported previously to have epiphyses. However, during preservation, the proximal end of the ulna in CGH142 was crushed, revealing a large pocket of potential cartilage- or spongy bone- cell spaces located proximal to a well-marked, robust edge of bone (Fig 27C). Additionally, although the morphology is unclear, a few specimens show weak evidence that epiphyses may have been present on the ends of the humerus and femur (Fig 27D and 27E).

### *Hyloplesion longicostatum*

#### Dermal Ossifications, Sculpture, and Lateral Lines

**Fig 28:**
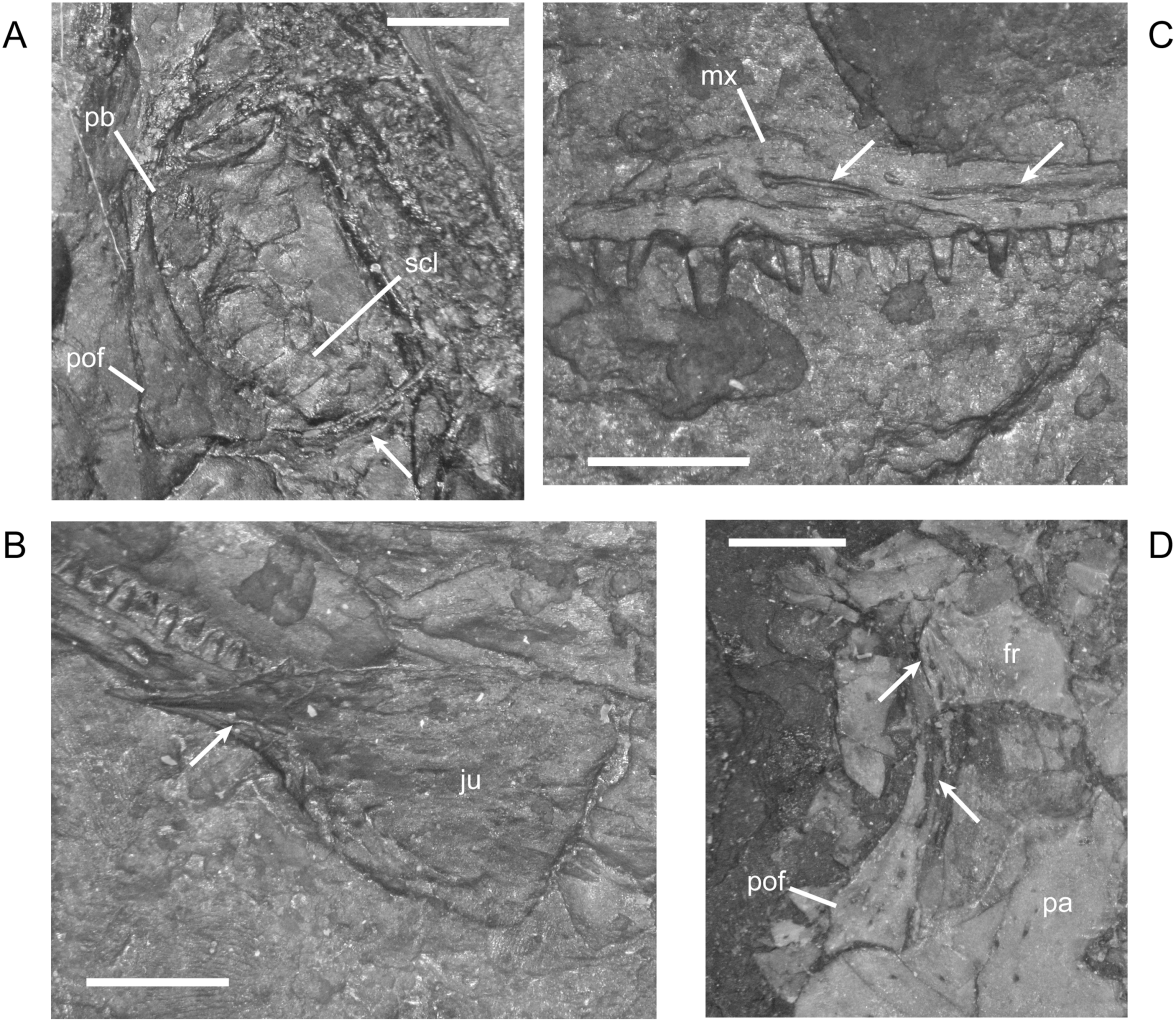
Palpebral bone and lateral lines in *H*. *longicostatum*. Arrows point to lateral line grooves and pits. A. Dorsal view of orbit of NHMW1898_X_23, showing palpebral bone and scleral ossicle impressions; anterior up, lateral to the right. Note lateral line groove on anterior edge of postorbital and crescentic shape of postfrontal. B. Dorsal view of jugal of CGH2028; anterior to the left, lateral up. C. Lateral view of maxilla of CGH3028; anterior to the left, dorsal up. D. Partial left orbital region of CGH16; dorsal view, anterior up, lateral to the left. Fr, frontal; Ju, jugal; Mx, maxilla; Pa, parietal; Pb, palpebral bone; Pof, postfrontal; Scl, scleral ossicles. Scale bars = 1mm.

The same two scale types described for *M. pelikani* also are found in *H. longicostatum*. However, the heavy ridge is not as strongly developed in the latter. No specimens of *H. longicostatum* have branchial plates, although they were reported in a specimen (now lost) collected from Třemošomá, Czech Republic [55]. Additionally, except for sporadic pitting and fine striae, *H. longicostatum* lacks sculpture on the dermal roofing elements. Sclerotic plates are present, but a palpebral cup was not identified previously in *H. longicostatum*. One specimen that I examined, NHMW1898_X_23, has an oval structure preserved within the orbit, but because of poor preservation it is unclear if the structure is a palpebral or a palatal fragment. However, two other specimens have impressions within the orbit that strongly resemble the palpebral impressions preserved in *M. pelikani* (Fig 28A). *Hyloplesion longicostatum* previously was reported to lack lateral lines [1], but I observed lateral line canal grooves on the maxillary (anterior) process of the jugal, the prefrontal (anterior) process of the postfrontal, the anterior edge of the postorbital, the lateral margin of the frontal, and the lateral surface of the maxilla (Fig 28A–28D). Moreover, as in *M. pelikani*, there are distinct pores located on the premaxilla, maxilla, and dentary, which also may connect to the lateral line system.

#### Snout and Dorsal Roof Elements

**Fig 29:**
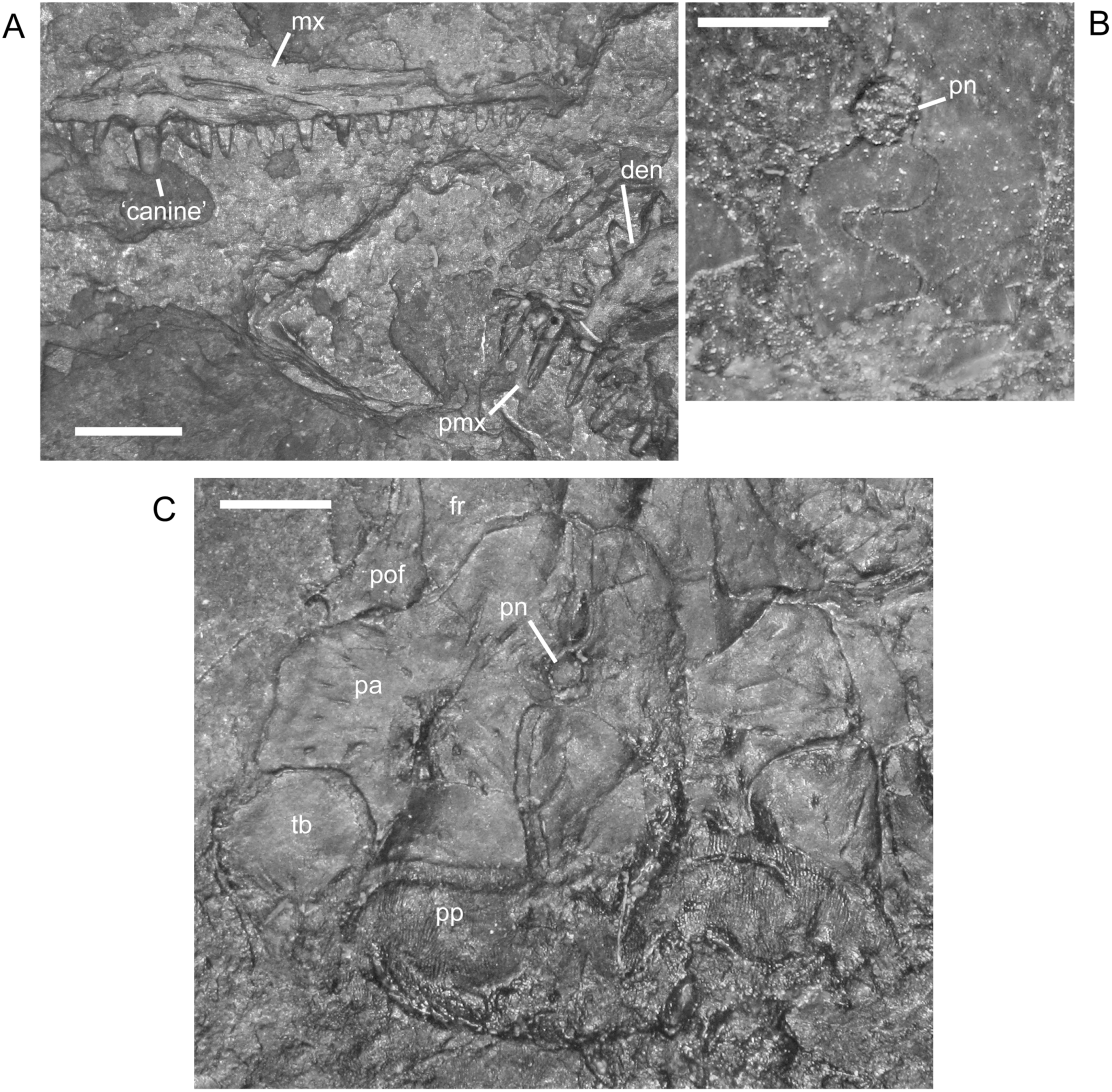
Teeth and dorsal skull of *H*. *longicostatum*. A. Maxilla, premaxilla, and dentary of CGH3028; anterior to the left. Note elongate teeth of premaxilla and enlarged ‘canine’ of maxilla. B. Midline suture between contralateral parietals of St.195; dorsal view, anterior up. C. Parietal, tabular, and postparietal of NHMW1898_X_23, showing crushed occipital surface; dorsal view, anterior up. Den, dentary; Fr, frontal; Mx, maxilla; Pa, parietal; Pmx, premaxilla; Pn, Pineal foramen; Pofr, postfrontal; Pp, occipital exposure of the postparietal; Tb, tabular. Scale bars = 1mm.

In contrast to the reconstruction provided by Carroll and Gaskill ([1] reference figure 89B), the premaxilla has a relatively long and narrow nasal process, resembling that of *M. pelikani*. The smallest specimen, CGH3 (skull length 3.9mm), has positions for four teeth in the premaxilla. The number of premaxillary teeth increases during ontogeny to reach a total of six or seven in larger specimens. Additionally, the spacing between the individual premaxillary teeth is wider in smaller specimens. The premaxillary teeth are relatively more elongate and narrow than the maxillary teeth (Fig 29A). Similar to the condition in *M. pelikani* both the premaxillary and maxillary teeth are slightly recurved at the distal tip. In contrast to the description by Carroll and Gaskill [1], the maxillary teeth in *H. longicostatum* are more triangular than those of *M. pelikani*, which are cylindrical pegs, although the smallest specimen of *H. longicostatum* has more slender and highly pointed teeth than other specimens of both taxa. The single, enlarged caniniform tooth of the maxilla [1] is present even in that smallest individual.

The sutures among elements of the skull table are relatively smooth and simple. However, in larger specimens, the contralateral parietals are united by an interdigitating suture of a distinctive morphology in which the ‘fingers’ are broad, rounded, and relatively sparse over the length of the contact (Fig 29B). Unlike the articulations in *M*. *pelikani*, the dermal roofing elements in *H*. *longicostatum* have extensively overlapping contacts. The nasal overlaps a squared, anterior shelf of the frontal, whereas the latter overlies a broad shelf of the parietal that extends from the midline laterally to the lateral lappet of the parietal (Fig 28D). Additionally, as figured by Carroll and Gaskill ([1] reference figure 89A,B), the parietal exhibits deep, rounded facets that underlie the anterior half of the posteriorly tapered, slightly curved tabular bones. The postparietals are not well-preserved in any specimen. However, in NHMW1898_X_23, the occipital flange of the postparietal is clearly exposed (Fig 29C).

#### Cheek and Circumorbital Elements

The dorsal exposure of the prefrontal in *H. longicostatum* closely resembles that of *M. pelikani*. However, in partially disarticulated *H. longicostatum* specimens the prefrontal has a moderately expanded medial edge that would underlie the nasal and the anterior end of the frontal. The postfrontal of *H. longicostatum* (Fig 28A and 28D) is much more slender and elongate than that of *M. pelikani*, in contrast to a previous depiction by Carroll and Gaskill [1]. The lacrimal participates in the external naris, and as in *M. pelikani*, the nasal and lacrimal are in contact dorsal to the prefrontal, excluding the latter from the naris. Although the squamosal does possess a narrow, medial shelf that underlies the tabular, it is unclear why prior descriptions [1] presumed that the squamosal extends far medially to contact the postparietal. Preservation of the posterolateral corner of the skull frequently is poor and no specimens exhibit clear evidence of such a contact. Additionally, in the reconstruction provided by Carroll and Gaskill ([1] reference figure 89B) the tabular is depicted too small relative to the rest of the skull (contrast with more realistic size in their figure 88, same specimen).

#### Palate

**Fig 30:**
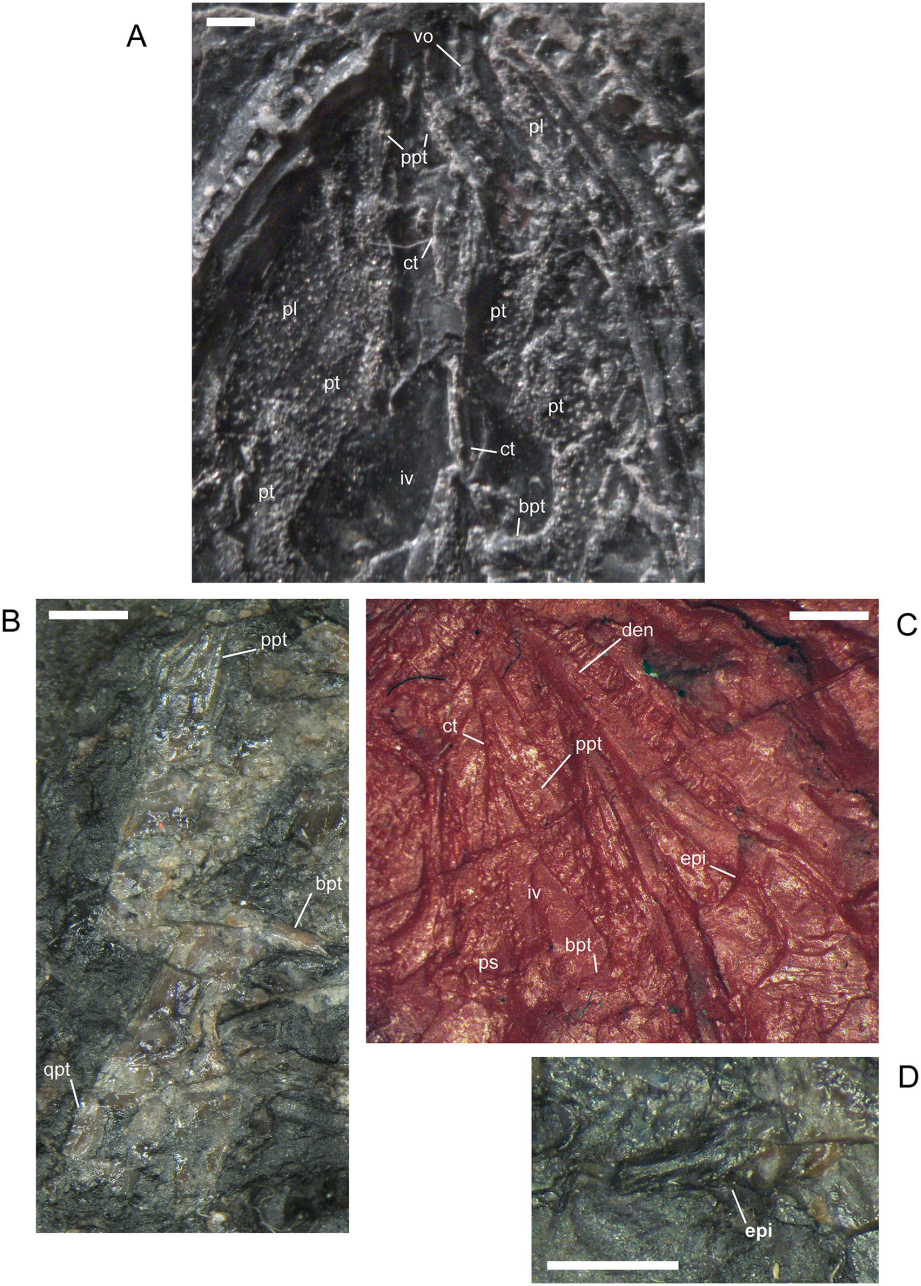
Palate of *H*. *longicostatum*. A. Anterior palate of RSM.1899.32.3; anterior up, ventral view, impression. B. Isolated pterygoid of M4885; Anterior up, lateral to the left, dorsal view, bone. C. Palate of NHMW1899_IX_8; anterior up, ventral view, latex cast. D. Isolated epipterygoid of M4885; bone. Bpt, basipterygoid process of pterygoid; Ct, cultriform process of the parasphenoid; Den, dentary; Epi, epipterygoid; Iv, interpterygoid vacuity; Ppt, palatine process of pterygoid; Pl, palatine; Ps, parasphenoid; Pt, pterygoid; Qpt, quadrate ramus of the pterygoid; Vo, vomer. Scale bars = 1mm.

My interpretation of the anterior palate does not agree with previously published descriptions [1]. The vomers do not have an extensive posterior, midline extension as was reconstructed ([1] reference figure 89). Much of that prior reconstruction was based on a single specimen, RSM.1899.32.3 (Royal Scottish Museum, Edinburgh, United Kingdom). The smooth structure interpreted as the complete vomer actually is a space in the palate, partially formed by separation of the vomers (Fig 30A). The matrix-filled space is continuous with the interpterygoid vacuities, which also were filled with fine sediment.

Secondly, the cultriform process of the parasphenoid extends much farther anteriorly than previously suggested. The roughened patch illustrated by Carroll and Gaskill ([1] reference figure 89E), located off-center from the anterior midline in RSM.1899.32.3, is actually the broken anterior tip of the cultriform process. Additionally, the short cultriform process previously figured for CGH3028 ([1] reference figure 89D) was based on the anterior termination of the denticle patch, which tapers and ends well posterior to the actual tip of the parasphenoid. Furthermore, I found no evidence for the ontogenetic changes of the parasphenoid previously reported, with the exception of the increase in denticle size and number [1]. The posterior plate of the parasphenoid is badly preserved in most specimens and there is no evidence for the missing posterior margin and lateral wings to be reconstructed in the manner illustrated previously ([1] reference figure 89). However, in cases in which the lateral wing is preserved, it appears broader and more rounded than in *M*. *pelikani*, with less lateral extension. The basipterygoid process of *H*. *longicostatum* is also relatively more elongate than that of *M*. *pelikani*.

As in *M*. *pelikani*, the pterygoid of *H*. *longicostatum* also extends far anteriorly, terminating in a triangularly pointed tip (Fig 30B). The pterygoid is located posteromedial to the vomer, although the vomers likely met along the midline anterior to the contact with the pterygoids. In RSM.1899.32.3, only a sliver of the left vomer is exposed under the lower jaw; the one on the right is completely covered. However, the anteromedial surface of the exposed vomer is oriented vertically, and a thin line of matrix marks where the contralateral vomers separated along that vertical contact. Other evidence for that arrangement of the palate is found in NHMW1899_IX_8 (Fig 30C), which also exhibits a broken epipterygoid, an element previously unknown in *H*. *longicostatum*. The epipterygoid is preserved most clearly in M4885, where it resembles those of *M*. *pelikani* and other ‘microsaurian’ lepospondyls (Fig 30D). Additionally, the palatine terminates further anteriorly than was depicted by Carroll and Gaskill [1] in their figure 89H, and thus is shorter than the maxilla in anteroposterior length.

There is no new information on the braincase, quadrate, or stapes, which are generally not well-preserved in *H*. *longicostatum*.

#### Lower Jaw

No ontogenetic changes or new information could be discerned about the mandible because too few are preserved for *H. longicostatum*.

#### Vertebrae

**Fig 31:**
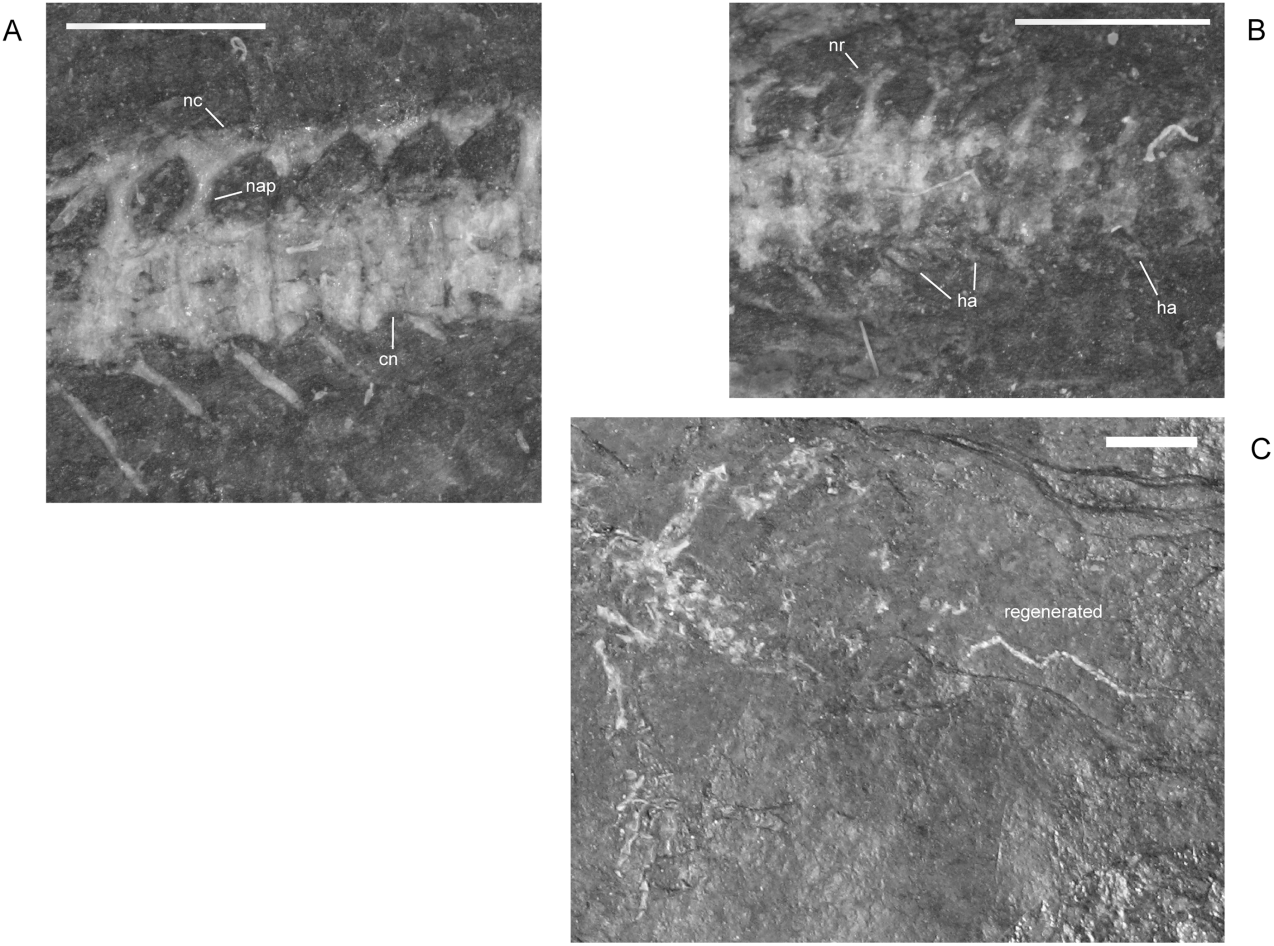
Vertebrae of *H*. *longicostatum*. A. Posteriormost presacral vertebrae of CGH3; anterior to the left, dorsal up; scale bar = 1mm. B. Caudal vertebrae of CGH3; anterior to the left, dorsal up; scale bar = 1mm. C. Posterior body of NHMW1898_X_23; Anterior toward top left; scale bar = 5 mm. Cn, centrum; Ha, haemal arch; Nap, neural arch pedicel; Nc, neural crest; Nr, neural arch.

Unlike *M. pelikani*, which is relatively conservative in the number of presacral vertebrae present (see above), *H. longicostatum* is more variable, with about half the specimens studied possessing 30 and the other half possessing 31. One specimen (M1377) may have as many as 32 presacral vertebrae, but preservation is relatively poor and a definite count is not possible. Even more so than in *M. pelikani*, the vertebrae in the smallest individuals appear boxy and angular, with a centrum height nearly equal to centrum length (Fig 31A). Morphometric data for *H. longicostatum* support the visual observation that the relative height of the centrum decreases with growth [17]. The angle of the incline of the neural arch is usually 5–10 degrees, but in one individual it measures between 10–20 degrees. The transverse processes are directed anterolaterally, as in *M. pelikani*.

For the very few individuals of *H*. *longicostatum* presenting a lateral view of the vertebrae, including the largest individual (RSM.1899.32.3), a suture is visible between the neural arches and centra. In at least some specimens, the two halves of the neural arches have separated, suggesting the elements were paired and not fused along the midline. However, in the two largest individuals, the neural arch halves are firmly associated with one another, suggesting that fusion occurs later in ontogeny ([1]; pers. obs.). In the smallest specimens the neural arch pedicels are dorsoventrally elongate but anteroposteriorly short, relative to the both the dorsal lamina of the arch and the centrum (Fig 31A). However, even in large individuals in which the pedicels are relatively longer, the structures are restricted to the anterior two-thirds of the centra, though the total length of the arches (prezygapophysis to spine tips) can be longer than the centra.

One specimen of *H*. *longicostatum* (RSM.1899.32.3) previously was reported to show tail regeneration and I agree with that suggestion ([1] reference figure 87B; pers. obs.). I also observed evidence for tail regeneration in an additional specimen, St.209 (Fig 31B). Mirroring the regenerated vertebrae of *M*. *pelikani*, the anterior neomorphic vertebrae in *H*. *longicostatum* are composed of a single element, whereas the posterior neomorphic vertebrae have dorsal and ventral structures.

Although haemal arches were reported to be absent [1], I observed clear haemal arches in the tails of three individuals of *H*. *longicostatum*, including the smallest specimen (Fig 31C). Presence is rare and uncorrelated with size, suggesting that haemal arches are subject to poor preservation [16]. As in *M*. *pelikani*, the haemal arches in *H*. *longicostatum* may be loosely sutured to the caudal vertebrae or may be weakly ossified.

#### Pectoral Girdle and Forelimb

Prior to my study, the interclavicle and scapula were known from only a single specimen, RSM.1899.32.3 ([1]; pers. obs.). Unfortunately, since that specimen was figured, more damage occurred to the area containing the pectoral girdle, resulting in a chunk of missing matrix that extends posteriorly to the mid-shaft of the right humerus. Much of the girdle is now missing or damaged. However, I observed an incomplete interclavicle in M1377 as well as evidence of anterior fimbriation in NHMW1898_X_23. Both of those specimens are smaller individuals than RSM.1899.32.3. The morphology of the scapula cannot be clearly observed in any other specimens. The clavicle and cleithrum of *H. longicostatum* are shaped like those of *M. pelikani*.

**Fig 32:**
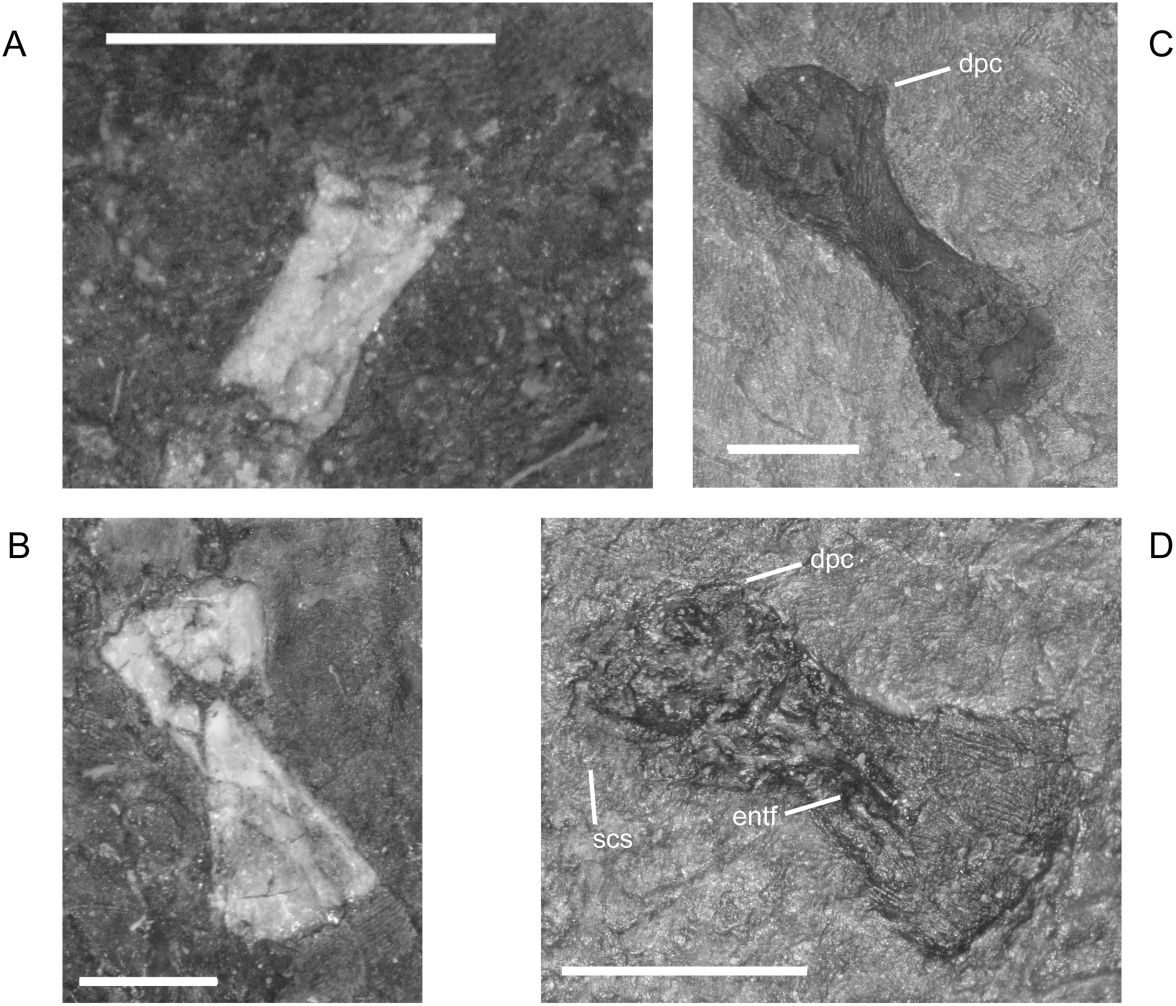
Ontogenetic changes in the humerus of *H*. *longicostatum*. Stage 5 not pictured. A. Stage 1, CGH3; proximal toward top right. B. Stage 2. CGH247; proximal toward top left. C. Stage 3, CGH3028; proximal toward top left. D. Stage 4, NHMW1898_X_23; proximal toward top left. Dpc, deltopectoral crest; Entf, entepicondylar foramen; Scs, subcoracoscapularis attachment point. Scale bars = 1mm.

In the smallest-known specimen of *H*. *longicostatum*, the humerus is a featureless column of bone with flat proximal and distal surfaces (Fig 32A). During the next stage of morphogenesis, the ends of the humerus become convex and can be distinguished from the shaft (Fig 32B). With further growth the proximal and distal ends of the humerus become highly domed relative to the condition in *M*. *pelikani* (Fig 32C). At the same level of morphogenesis a small, distally located deltopectoral crest is present. Next, a small attachment point for the subcoracoscapularis develops (Fig 32D). Finally, the distal condyles are differentiated, including the formation of a shallow fossa or groove between them. I did not observe any clear torsion in the humeri of the specimens that I examined, but many are broken or poorly preserved. One specimen (NHMW1898_X_23; Fig 32D) has an entepicondylar foramen, which is oval and elongate, as in *M*. *pelikani*. Presence of that foramen was unreported in prior studies of *H*. *longicostatum*.

**Fig 33:**
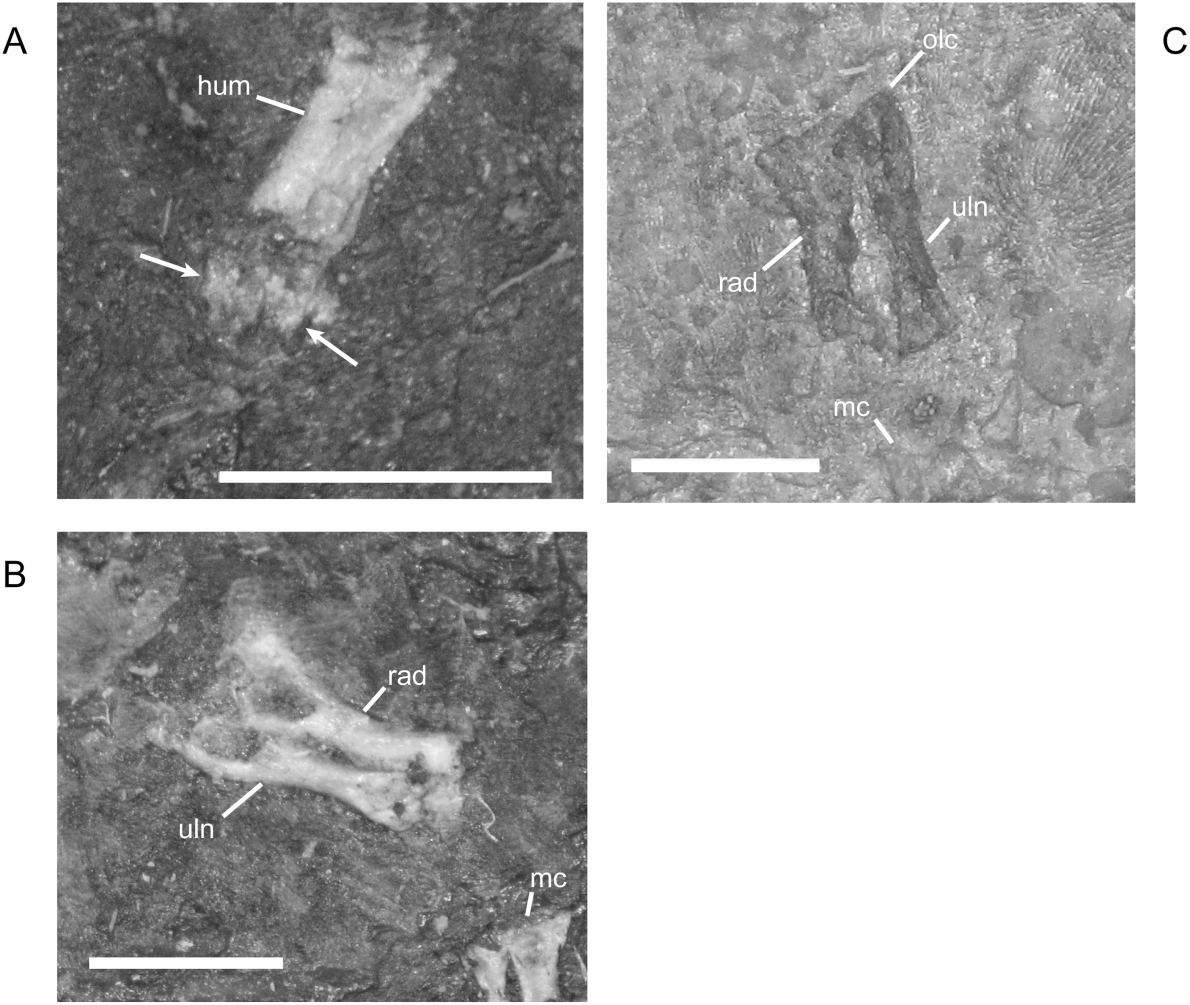
Ontogenetic changes in the radius and ulna of *H*. *longicostatum*. A. Stage 1, CGH3; proximal toward upper right. Arrows point to radius and ulna. The two elements are poorly ossified and cannot be distinguished from one another. B. Stage 2, CGH247; proximal toward upper left. C. Stage 3, CGH2038; proximal toward top. Beyond this stage, the olecranon becomes a rounded, distinct process. Hum, humerus; Mc, metacarpals; Olc, olecranon process; Rad, radius; Uln, ulna. Scale bars = 1mm.

The radius and ulna of the smallest specimen are incompletely ossified (Fig 33A). Morphogenetic changes in both elements begin with the differentiation of the shaft from the proximal and distal ends (Fig 33B). The ends of the radius and ulna are highly domed. As in *M*. *pelikani*, the proximal end of the ulna always is broader than the distal end of the element, and the two ends of the radius are approximately equal in size. During the next stage of growth the olecranon process of the ulna begins to form, starting off as a lateral-medial slant of the proximal surface of the bone (Fig 33C). At the same level of development, the medial surface of the distal end of the radius is slightly modified for the intermedial facet. Further growth of the ulna leads to a more prominent and rounded olecranon process.

#### Pelvic Girdle and Hindlimb

**Fig 34:**
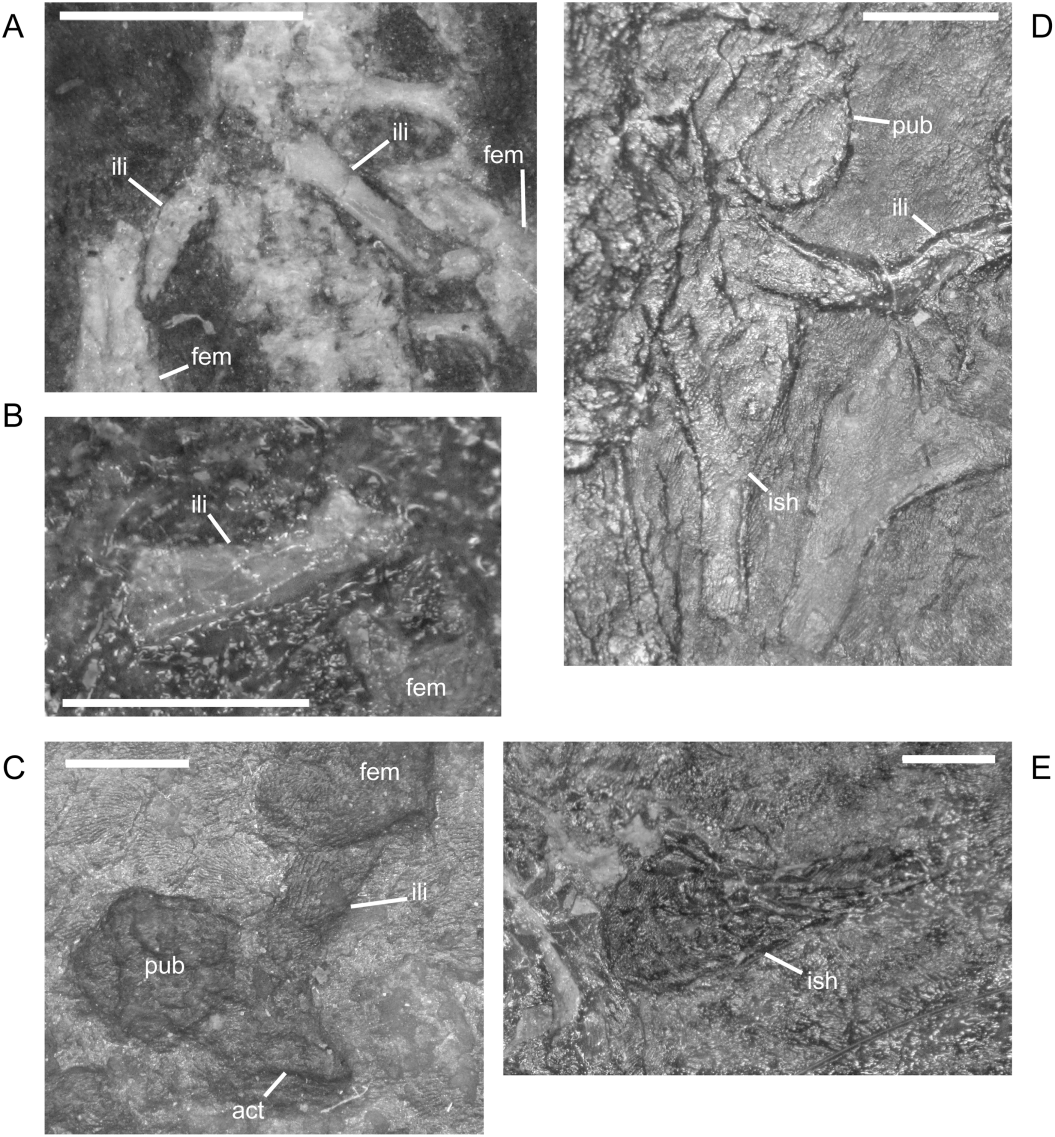
Ontogenetic changes in the pelvic girdle of *H*. *longicostatum*. A. Stage 1 of ilium, CGH3; Anterior up, elements from both sides present. B. Stage 2 of ilium, CGH45; Anterior up, proximal to the left. C. Stage 3 of ilium, CGH3028; proximal ilium points downward. Note that femur is disarticulated from ilium. D. Ischium and pubis of NHMW1898_X_23; anterior up, proximal toward left. E. Ischium of St.195. Act, acetabulum; Fem, femur; Ili, ilium; Ish, ishium; Pub, pubis. Scale bars = 1mm.

Similar to *M. pelikani*, the ilium of *H. longicostatum* ossifies relatively early in ontogeny. In one of the smallest individuals, the ilium is a featureless, narrow, bone (Fig 34A). The base is only partially ossified. During the next stage of growth, the base is rounded and distinct, but not expanded. The dorsal process, however, appears bifurcated in one of the specimens at that level of development, though the appearance may be the result of crushing (Fig 34B). In developmentally more advanced specimens, the acetabulum is differentiated, the base is expanded, and the dorsal process is curved posterodorsally (Fig 34C).

As in *M*. *pelikani*, the ischium of *H*. *longicostatum* starts out as a triangular bone with no distinct features (Fig 34D). In specimens of all sizes, the posterior process of the ilium in *H*. *longicostatum* is relatively narrower and more elongate than in *M*. *pelikani*. With further growth, the medial edge of the element becomes progressively convex, resulting in an angular anteromedial process (Fig 34E).

The pubis is present in only two specimens of *H*. *longicostatum*. It is better preserved in NHMW1898_x_23, which shows that the bone is subcircular, relatively flat, and ringed by a thin ridge around the external edge of the element (Fig 34D).

**Fig 35:**
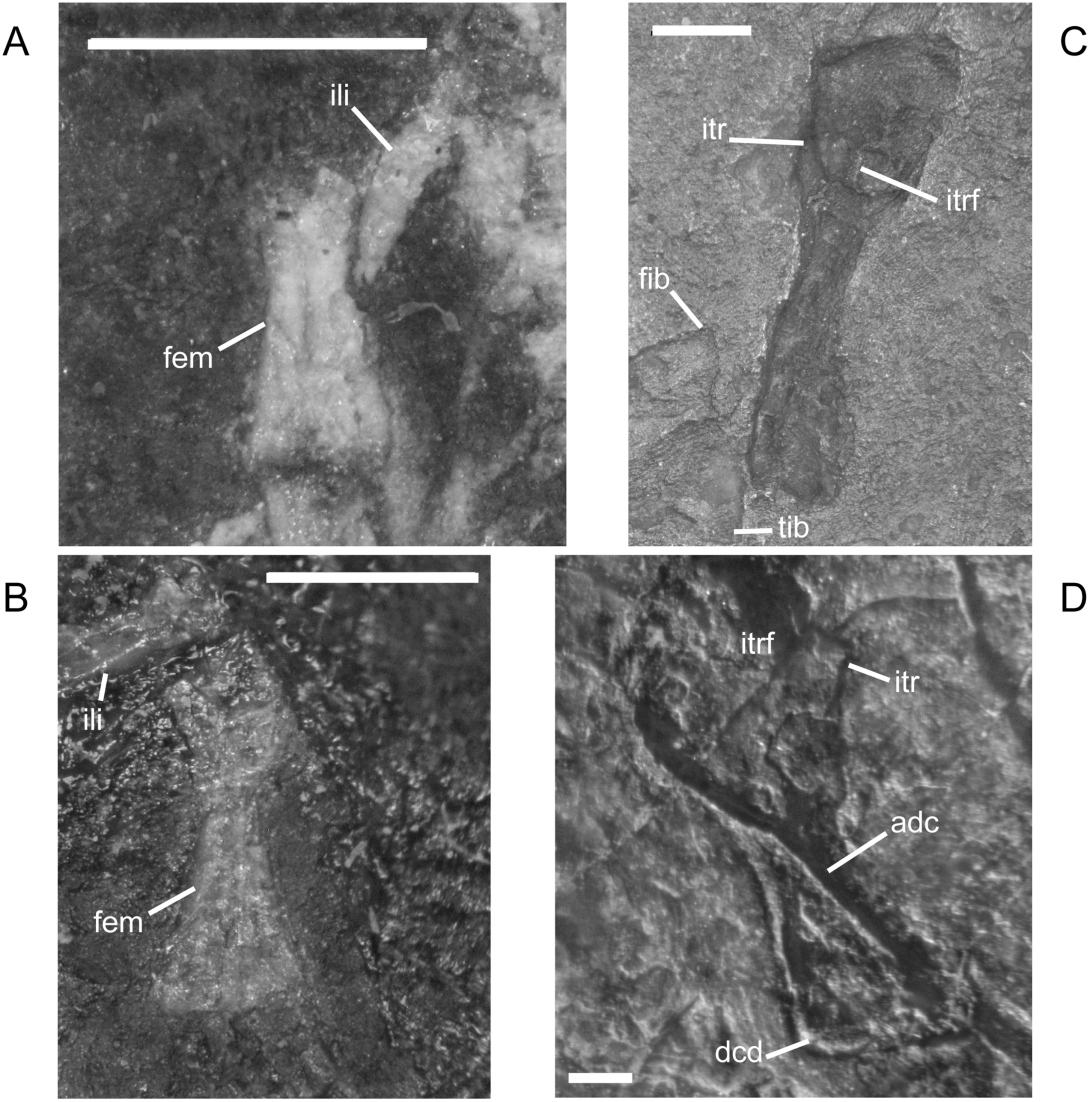
Ontogenetic changes in the femur of *H*. *longicostatum*. A. Stage 1, CGH3; proximal toward top. B. Stage 2, CGH45; proximal toward top. C. Stage 3, CGH3028; proximal toward top. D. Stage 4, RSM.1899.32.3 (Royal Scottish Museum, Edinburgh, United Kingdom); proximal toward upper left. Adc, adductor crest; Dcd, distal condyles; Fem, femur; Fib, fibula; Ili, ilium; Itr, internal trochanter; Itrf, intertrochanteric fossa; Tib, tibia. Scale bars = 1mm.

After initial ossification, the femur of *H*. *longicostatum* is a simple column of bone with a gradual distal expansion, but no distinct features (Fig 35A). The ends of the femur are incompletely ossified at that stage of development. With increased growth, the shaft becomes distinct from the proximal and distal ends of the femur (Fig 35B). Later in ontogeny, the ends of the femur become highly rounded, much more so than in *M*. *pelikani*, and the intertrochanteric fossa is prominent (Fig 35C). Concomitantly, the internal trochanter appears as a small, but distinct, process. The adductor crest forms later in ontogeny, along with partial differentiation of the distal condyles (Fig 35D).

**Fig 36:**
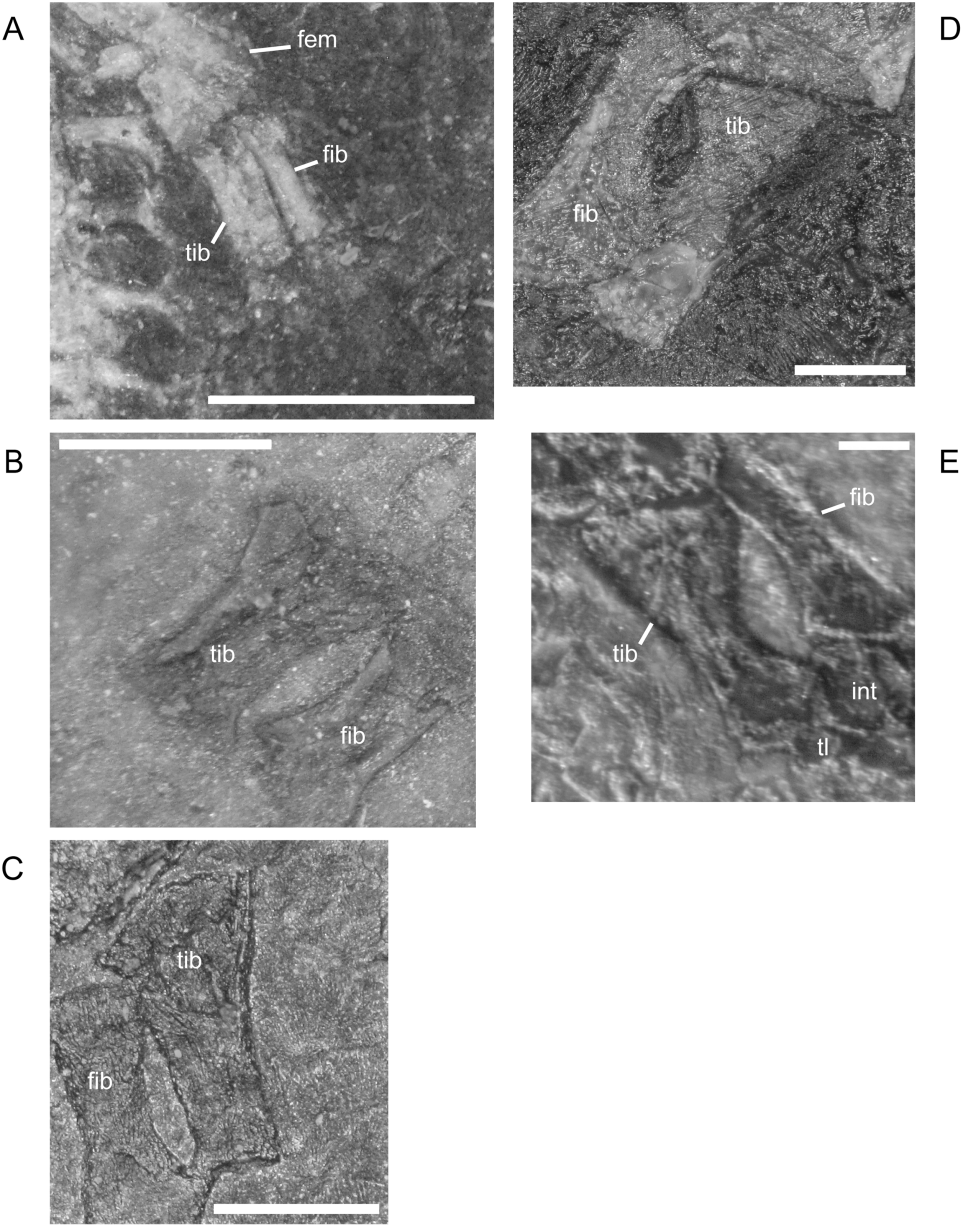
Ontogenetic changes in the tibia of *H*. *longicostatum*. A. Stage 1, CGH3; proximal toward top. B. Stage 2, St.152; proximal toward upper right. C. Stage 3, NHMW1898_X_23; proximal toward top. D. Stage 4, St.195; proximal toward upper right. E. Stage 5, RSM.1899.23.3; proximal toward upper left. Fem, femur; Fib, fibula; Int, intermedium; Tib, tibia; Tl, tibiale. Scale bars = 1mm.

In the smallest known individual of *H*. *longicostatum*, the tibia is a block of bone with straight sides, unossified ends, and no identifiable features (Fig 36A). During the next stage of development, the proximal end of the tibia is greatly expanded (Fig 36B). Following that change, the intermedial facet of the tibia is developed as a medial slant at the distal end of the element (Fig 36C). Additionally, the head of tibia begins to slant medially. At the same stage of growth or soon after, the medial margin of the tibia becomes more concave, resulting in overall curvature (Fig 36D). In one large individual the facet for the tibiale is apparent as a lateral slant in the distal end of the tibia (Fig 36E).

**Fig 37:**
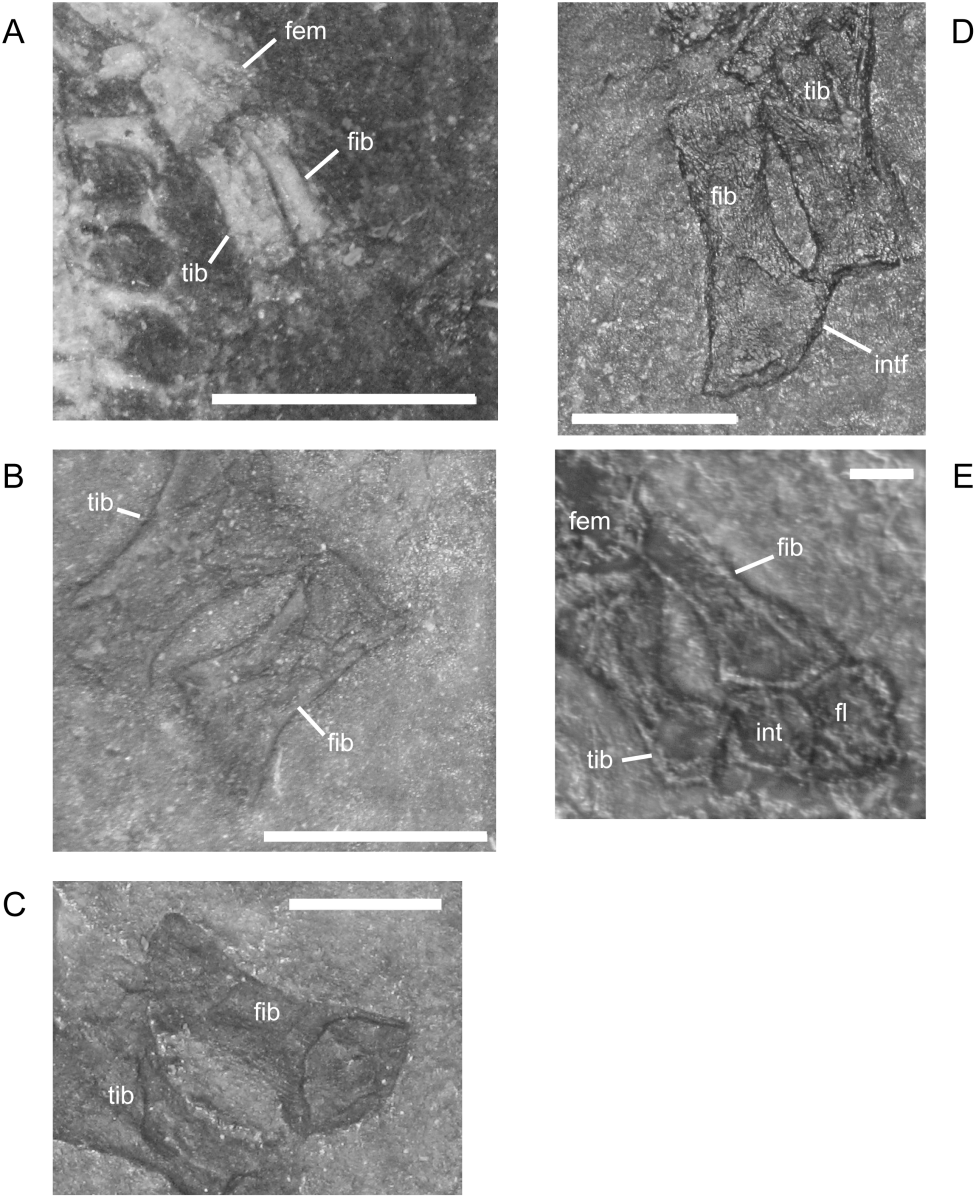
Ontogenetic changes in the fibula of *H*. *longicostatum*. A. Stage 1, CGH3; proximal toward top. B. Stage 2, St.152; proximal toward upper right. C. Stage 3, CGH3028; proximal toward upper left. D. Stage 4, NHMW1898_X_23; proximal toward top. E. Stage 5, RSM.1899.32.3; proximal toward upper left. Fem, femur; Fib, fibula; Fl, fibulare; Int, intermedium; Intf, intermedial facet; Tib, tibia. Scale bars = 1mm.

The least well-developed fibula that I observed is a narrow column of bone with flat, unfinished ends (Fig 37A). The first morphogenetic change in the fibula is the expansion of the distal end of the element (Fig 37B). As in *M*. *pelikani*, expansion is asymmetric, so that the medial surface of the distal end projects further. Shortly thereafter, the fibula exhibits moderate curvature caused mainly by the medial margin of the shaft becoming concave (Fig 37C). In the next stage of development the distal end of the fibula slants medially to produce the intermedial facet (Fig 37D). Late in ontogeny a distinct facet for the fibulare is visible and the bone is more strongly curved overall (Fig 37E).

#### Epipodials, Metapodials, and Phalanges

Multiple carpals were reported in a single individual of *H. longicostatum* by Caroll and Gaskill [1]. The poor preservation of the forelimb in that specimen made confirmation difficult. At most there may be 2–3 carpals present, but even those structures are not clearly visible. No other specimen of *H. longicostatum* that I examined has ossified carpals.

A higher number of tarsals is preserved in specimens of *H*. *longicostatum* than *M*. *pelikani*. The maximum number reported was eight, although more appear to be figured by Carroll and Gaskill ([1] reference figure 90I). I examined the specimen in question (RSM.1898.23.3) and can confirm the presence of at least seven tarsals. Based on specimens with only a single tarsal present, the intermedium ossifies first. The fibulare is much larger than the tibiale and probably ossifies next, although an additional specimen that may have two tarsals appears to have an intermedium and weakly ossified tibiale or centrale. The tarsal labeled as the ‘fibulare’ by Carroll and Gaskill ([1] reference figure 90I) may be a fusion of two elements.

In the least mature specimen of *H*. *longicostatum* (CGH3), the metapodials and phalanges are shaped like rectangles, with no differentiation of articular surfaces. However, the distal-most phalanges already exhibit the distinct, sharply pointed morphology seen in larger specimens. As growth continues, the proximal and distal ends expand and the phalanges elongate. As expansion continues, the metapodials and phalanges of moderately mature specimens appear relatively angular in shape because the ends remain flat. In more mature specimens, however, the ends are convex and the margins of the metapodials and phalanges are more smoothly curved. Additionally, the metapodials of many moderately to largely mature specimens have a roughly triangular groove or depression located near both their proximal and distal ends. The overall pattern observed in a few individuals with articulated autopods (e.g., NHMW1898_X_23) suggests that the metapodials are generally better ossified than the phalanges, and the posterior (ulnar/fibular) digits undergo morphogenetic changes before the anterior digits, with the exception of digit 5,which often is developed poorly. Metatarsal 5 is especially weakly ossified in some individuals (e.g., NHMW1898_X_23; St. 195), and the phalanges of digit 5 also are more slender than those of the other digits in the foot. As noted earlier, however, all of the distal-most phalanges do attain their mature morphology at the earliest stages of growth.

#### Phylogenetic Analyses

Scoring modifications for *M*. *pelikani* and *H*. *longicostatum* are summarized in S3 and S4 Tables, and discussed below. Complete matrices are provided as S1 and S2 Files. I successfully reproduced the strict consensus tree and tree statistics published by Ruta and Coates ([6] reference figure 5), although the number of Most Parsimonious Trees (MPTs) that I obtained, 13, were far fewer than the 324 from the original publication. Subsequent modification of the scores for *Microbrachis* and *Hyloplesion* resulted in 14 MPTs and a tree length of 1579, five steps shorter than in the original analysis published by Ruta and Coates [6]. Similar to the results obtained by Ruta and Coates [6], the strict consensus tree from my analysis is highly resolved (S1 Fig), but with less resolution among basal stem amniotes. *Microbrachis* and *Hyloplesion*, although still sister taxa, are basal to all remaining lepospondyls in my tree. As a result of that basal displacement, the ‘Microsauria’ clade of Ruta and Coates [6], which included *Microbrachis* and *Hyloplesion*, now has a basal polytomy consisting of *Saxonerpeton*, a *Batropetes*-*Ophiderpeton* clade, and a *Hapsidopareion*-Recumbirostra (*sensu* [3]) clade. All remaining relationships are the same as those reported by Ruta and Coates [6].

I could not reproduce exactly the original strict consensus topology presented by Huttenlocker et al. [5]. The tree length was shorter (1125), although my analysis returned the same number of MPTs (6). The only major differences in my strict consensus of Huttenlocker et al.’s [5] unmodified data were the positions of *Utaherpeton* and *Microbrachis*. In Huttenlocker et al. [5] *Utaherpeton* is nested within a lepospondyl clade containing aistopods, lysorophoids, and nectrideans whereas *Microbrachis* is the basal-most taxon of a second lepospondyl clade containing the remaining traditional ‘microsaurs’. In my strict consensus, as in that of Anderson et al. [4], *Utaherpeton* is the sister taxon to all other lepospondyls and *Microbrachis* is the basal-most taxon of the lepospondyl clade containing aistopods, lysorophoids, and nectrideans (S2 Fig). Additionally, in my strict consensus, nectrideans are paraphyletic with respect to aistopods and lysorophoids, also matching the topology of Anderson et al. [4] rather than Huttenlocker et al. [5].

Modification of the scores for *Microbrachis* and the addition of *Hyloplesion* resulted in unstable relationships within Lepospondyli, reflected as a large polytomy among basal lepospondyls in the strict consensus (tree length = 1180; 48 MPTs; S3 Fig). One of the sub-clades within the burrowing lepospondyl group ‘Recumbirostra’ (see [5]) is maintained, but relationships within that group also are less stable, except for the more derived taxa. Relationships involving aistopods, nectrideans, and lysorophoids were less stable, as well, although detailed relationships among nectridean taxa were retained. *Hyloplesion* and *Microbrachis* were recovered as sister taxa; Utaherpeton remained the sister taxon to all other lepospondyls.

## Discussion

### Implications of Character Coding and Scoring

A number of the new osteological and ontogenetic observations are relevant for understanding and gauging the importance of existing morphological characters for interpreting lepospondyls and early tetrapod relationships. My objective was to draw attention to scoring modifications for *Hyloplesion* and *Microbrachis*, and thus I evaluated characters only from phylogenetic analyses that included both as ingroup taxa (i.e., [3,6,47]). Previously *Hyloplesion* could not be scored for many characters described by Anderson [3] and Ruta and Coates [6], and Anderson et al. [4] excluded that taxon because of incomplete data. The revisions presented here, however, allowed for the addition of *Hyloplesion* to the matrix of Huttenlocker et al. [5] (equals that of [4]), which required new character scoring in addition to modifications of scores provided by Anderson [3] and Ruta and Coates [6]. Recall that I chose to use the original matrices of Huttenlocker et al. [5] and Ruta and Coates [6] for consistent comparison of lepospondyl relationships, but that Sigurdson and Green [8] published extensive revisions to the matrices of Anderson et al. [4] and Ruta and Coates [6]. Future analyses focused on comprehensive reconstructions of tetrapod relationships should include those revisions, as well as the additional taxa and corrections made by Maddin et al. [56] and Huttenlocker at al. [5], and the modifications for *Hyloplesion* and *Microbrachis* presented here.

#### Skull

One of the most important revisions is the presence of lateral lines in *H. longicostatum*, which requires scoring changes for character 46 of Anderson ([3]; [5], character 64) and characters 120 and 121 of Ruta and Coates [6]. Also, although *M. pelikani* appears to have ossified branchial plates, I did not find evidence for an ossified hyoid ([3], character 111; [5], character 141].

Reinterpretation of the region around the external naris in *M*. *pelikani* affects character 10 of Anderson ([3]; [5], character 14), which describes whether or not the lacrimal participates in the narial margin. In both *M*. *pelikani* and *H*. *longicostatum*, the lacrimal should participate in the narial margin. Carroll and Gaskill [1] suggested that a large septomaxilla intervenes in *M*. *pelikani*, but that would be highly unusual among basal lepospondyls and there is no evidence to support that reconstruction. It is possible that the septomaxilla is unossified (or absent) in *M*. *pelikani* and *H*. *longicostatum* because the element was never identified in either taxon. Previously, *H*. *longicostatum* was scored as ‘ossified’ by Anderson ([3] character 20; [5], character 28).

Additionally, Anderson ([3], character 17) scored the participation of the frontal in the orbital margin as ‘present’ in both *M*. *pelikani* and *H*. *longicostatum*. That scoring is in error because the contact between the prefrontal and postfrontal excludes the frontal from the orbit. The codings for *M*. *pelikani* and *H*. *longicostatum* were corrected by Huttenlocker et al. ([5], character 22). Character 9 of Huttenlocker et al. [5], describes the shape of the postfrontal as either falciform or quadrangular. The postfrontal of *H*. *longicostatum* is falciform (previously not included, now state 1). The original coding for *M*. *pelikani*, quadrangular (state 0), was retained, even though the character description is not entirely accurate. The postfrontal in that taxon is slender and curved (falciform) anteriorly, but ends in a quadrangular flange or process posteriorly. Similarly, I scored *H*. *longicostatum* as possessing an interdigitating lacrimal-prefrontal suture (state 1, Character 12, [5]), even though the contact appears to be more of an abutment. Most of the ‘microsaurian’ lepospondyls, including *M*. *pelikani*, were scored as ‘interdigitating’ in the original analysis. *Hyloplesion longicostatum* has the same morphology as *M*. *pelikani* and many of the other taxa, in which there also is not strong evidence for an interdigitating suture, and so *H*. *longicostatum* was scored in the same way for consistency. In the future, the character merits re-description.

In contrast to prior reports [9] and illustrations [1], there is not a contact between the parietal and squamosal in *M*. *pelikani* ([3], character 35; [5], character 51); the postorbital and tabular prevent contact between those elements. Additionally, there is not strong support for a prior inference [1] of a contact between the squamosal and postparietal ([3], character 40; corrected in [5], character 56). There also is no evidence for the positive identification of a supraocciptal in *H*. *longicostatum* ([3], character 65; [5], character 83) nor *M*. *pelikani* ([47], character 19); both should be coded as ‘?’. The structure in *H*. *longicostatum* that was described as a supraoccipital [1] is poorly preserved and could alternatively be identified as the ventral surface of the postparietal. Similarly, the new braincase characters of Huttenlocker et al. ([5], characters 221–223) cannot be scored for either *H*. *longicostatum* or *M*. *pelikani* because the necessary views are not well-preserved in any individuals; scores for these characters should be ‘?’ for both taxa.

Previous reconstructions of the anterior palate in *H*. *longicostatum* are incorrect [1]. First, in that taxon the maxilla actually is longer than the palatine, so for character 70 of Huttenlocker et al. [5], the state should be ‘0.’ There is also evidence in *M*. *pelikani* and *H*. *longicostatum* that both the contralateral pterygoids and contralateral vomers met anteriorly. Previously, it was suggested that the vomers are relatively large and prevent the pterygoids from meeting [1]. However, the vomers and their association with the pterygoids resemble the condition in *M*. *pelikani*. My reinterpretation changes the scoring of *H*. *longicostatum* for characters 123 and 125 of Ruta and Coates [6], character 10 of Zanon ([47]; see also character 12), character 90 of Anderson [3], and character 116 of Huttenlocker et al. [5]. In the latter two, *M*. *pelikani* and *H*. *longicostatum* were listed as lacking anterior contact between the pterygoids, but in both taxa the pterygoids meet at the midline. Similar changes in scoring for both taxa should be made for character 150 of Ruta and Coates [6]. There is not enough of the palatine exposed in *H*. *longicostatum* to decide whether the anterior margin of that element is ‘short’ or ‘long,’ and so that taxon should be scored as ‘?’ (unknown) for character 70 of Huttenlocker et al. [5]. In addition, the vomer of *H*. *longicostatum* lacks fangs and should be scored the same as *M*. *pelikani* for character 123 of Ruta and Coates [6]. The ectopterygoid of both microsaurs also lacks pit fangs ([5], character 121), and therefore the original score of ‘present’ for *M*. *pelikani* should be corrected to ‘absent.’ Finally, Ruta and Coates [6] scored character 176, presence/absence of strut-like cultriform process, differently for *M*. *pelikani* and *H*. *longicostatum*. The state should be the same in both taxa because the cultriform process is present, although it is not as strut-like as in many temnospondyls. The articulation of the stapes and parasphenoid is not preserved well-enough in any specimen of *H*. *longicostatum* to score character 104 of Huttenlocker et al. ([5]; [3], character 81] as any state other than ‘?’ (unknown).

#### Vertebrae

New ontogenetic data about the vertebrae of *M. pelikani* and *H. longicostatum* suggest that the neural arch retains a suture with the centrum until relatively late in development, when the two elements fuse ([3], character 117; [5], character 149; [6], characters 303, 314). Related to that change, the neural arches of the trunk in both of the microsaurs are initially paired in less mature individuals, but fuse into a single structure later in ontogeny ([3], character 129; [5], character 163). Additionally, neither taxon possesses centra with striated ornamentation ([6], character 320).

Previously, haemal arches were unreported in *H*. *longicostatum*, but I observed them in a number of specimens, which has consequences for character 123 of Anderson [3] and character 156 of Huttenlocker et al. [5]. In neither *M*. *pelikani* nor *H*. *longicostatum* are the haemal arches fused to the centra ([3], character 124; [5], character 157; [6], character 308). The neural and haemal arches are not aligned dorsoventrally in either taxon ([6], character 307), nor are there extra articulations or proximal emarginations on the haemal spines ([6], characters 309 and 319). The haemal arches are equal to or longer than the neural arches in both *M*. *pelikani* and *H*. *longicostatum* ([3], character 125; [5], character 158). Also, based on the presence of haemal arches in the tail, the centrum is considered to be the pleurocentrum ([13]; [6], characters 310–312). As in *M*. *pelikani*, trunk intercentra are absent in *H*. *longicostatum* ([6], character 316). The tail of *M*. *pelikani* is foreshortened relative to trunk length; however in *H*. *longicostatum* the tail is too incompletely known to score character 162 of Huttenlocker et al. [5] as anything other than ‘?’, even though, based on partial tails, the region probably is foreshortened as well.

#### Pectoral and Pelvic Girdles

The present study provides new information on the interclavicle of *H. longicostatum*, which was not described fully in the past. In the analysis by Anderson [3], character 150 (see also [5], character 186) was not scored for that taxon because it was unknown if the interclavicle was diamond- or T-shaped. Although I did not observe a complete stem in *H. longicostatum*, the overall shape of the interclavicle is the same as that of *M. pelikani*, which implies that the element is T-shaped when complete. However, character 240 of Ruta and Coates [6] should remain scored as “?” for *H. longicostatum* because the exact nature of the stem is unknown. As in *M. pelikani*, the interclavicle is wider than long ([6], character 241). Also, the first record of a partially ossified coracoid in *M. pelikani* affects previous scoring for character 159 of Anderson [3] and character 198 of Huttenlocker et al. [5], which coded the element as ‘unossified.’ The number of coracoid foramina is unknown in both *M. pelikani* and *H. longicostatum*; both taxa should be scored as ‘?’ for character 158 of Anderson [3] and character 197 of Huttenlocker et al. [5]. Finally, in the pelvic girdle of a large individual of *H. longicostatum*, the ilium is slightly bifurcated at the dorsal tip rather than terminating as a single blade ([3], character 170; [5], character 211; [6], character 272). The state of that character is dependent upon the maturity of the specimen because less-developed individuals do not exhibit bifurcation.

#### Limbs

Previously, the entepicondylar foramen was thought to be absent in *H. longicostatum* ([3], character 160; [5], character 199; [6], character 253; [47], character 7). However, I observed the foramen in a single individual. Other specimens of *H. longicostatum* are not preserved in an orientation in which the foramen would be visible, although ontogenetic variation cannot be excluded. The amount of torsion present in the humerus is affected by the degree of morphogenesis in both *H. longicostatum* and *M. pelikani* ([3], Character 161; [5], character 200). In developmentally younger individuals, the torsion may be as low as 0–30°. However, in mature specimens the torsion approaches 90°. The prominence of the deltopectoral crest ([3], character 162; [5], character 201) also is subject to ontogenetic variation. In both taxa, the crest is absent in the least mature specimens. The structure becomes distinct in large individuals, although it never reaches the relative size or degree of prominence found in other lepospondyls like *Pantylus*, and many large temnospondyls. A distinct ectepicondylar ridge is not developed in either *M. pelikani* or *H. longicostatum* ([6], character 255, previously unscored for *M. pelikani*). Presence/absence of a waisted humeral shaft, character 258 of Ruta and Coates [6], was incorrectly scored for *M. pelikani*. In both taxa the shaft is waisted except during the earliest stage of development. Additionally, for *H. longicostatum*, which was unscored by Ruta and Coates [6] for character 259, the radial condyle is located ventrally as in *M. pelikani*.

The short olecranon process of *M*. *pelikani* is ossified late in development, despite its absence or weak development in less mature specimens ([3], character 166; [5], character 205; [6], character 271). The average ratio between the humerus and radius in *H*. *longicostatum* was previously thought to be less than in *M*. *pelikani*. However, the ratios are similar and the average in *H*. *longicostatum* is 0.55 (range 0.47–0.70), rather than below 0.5, as was reported in prior studies ([3], character 165; [5], character 204). Also, in both taxa the ulna is approximately the same length as the radius ([6], character 269).

In both *M*. *pelikani* and *H*. *longicostatum*, when the femur initially ossifies, the intertrochanteric fossa is not yet developed. However, with increased growth the fossa becomes distinct, in contrast to the score of ‘absent’ in Anderson [3] for character 172 (see also modified character 212, [5]). Additionally, the femora of *M*. *pelikani* and *H*. *longicostatum* were scored differently for character 174 of the same study, which describes whether the femur is ‘short’ or ‘long’ (see also [5], character 214). In actuality, the ratio between femur length and skull length is nearly the same in *M*. *pelikani* (average 0.31, range 0.23–0.38) and *H*. *longicostatum* (average 0.36, range 0.25–0.47).

The shape of the tibia also changes during ontogeny. Although the tibia was correctly scored ([3], character 175) as expanded distally for *H*. *longicostatum*, that of *M*. *pelikani* was scored as ‘absent.’ As in *H*. *longicostatum*, however, the distal end of the tibia in *M*. *pelikani* expands with growth. For character 291, number of proximal tarsals, Ruta and Coates [6] did not score *M*. *pelikani*. One specimen that I observed has two proximal tarsals, although none of the tarsal ossifications are L-shaped ([6], character 292).

#### Consequences of Phylogenetic Analyses

Results from my re-evaluation of prior analyses using revised character scores agree with previous findings that ‘microsaurs’ are paraphyletic and part of the amniote stem, with a sister relationship between *Microbrachis* and *Hyloplesion* [4–6]. However, resolution among stem amniotes was decreased by the integration of new ontogenetic and anatomical data for *H. longicostatum* and *M. pelikani*, indicating that sufficient ontogenetic and morphological data, as well as taxon sampling, are lacking for early amniotes and their Paleozoic relatives, including lepospondyls.

### Comparison of Postcranial Morphogenesis and Implications for Paedomorphosis

The development of skeletal morphology in early tetrapods is relatively conservative. As demonstrated by a large survey on postcranial morphogenesis in temnospondyls, differences between related groups frequently can be attributed to shortening (paedomorphosis) or lengthening (peramorphosis) of ontogenetic trajectories [36], which may be caused by changes in the onset or offset of developmental events [29]. Paedomorphosis of limbs and girdles is manifested as less-well developed elements in adults. The prominent processes and rugosities present on elements of adults of taxa unaffected by paedomorphosis are smaller, ill-defined, or absent in their relatives that do exhibit paedomorphic features [36].

The completion of postcranial development in early tetrapods such as *Greererpeton*, *Eryops*, and basal synapsids leads to relatively strongly developed limbs and girdles [36,51,57]. A mature humerus, for example, has a strongly developed deltopectoral crest, supinator processes, and capitellum (swollen, radial condyles). Those features are also evident in the well-developed humerus of *Pantylus*, one of the largest lepospondyls [1]. In the most mature *H*. *longicostatum* and *M*. *pelikani*, however, the supinator process is absent and the deltopectoral crest and radial condyle are developed only moderately. It may be the case that even more mature specimens of these two taxa have not yet been discovered. However, based on current data about the morphology of the humerus and other appendicular elements, and the relatively small size of adults, it is likely that the limbs of *H*. *longicostatum* and *M*. *pelikani* are paedomorphic relative to those of many basal tetrapods, stem amniotes, large temnospondyls, and large lepospondyls. Importantly, paedomorphosis may not affect all features of an element equally. In *Greererpeton*, for example, even though crests and tuberosities are strongly developed, adults do not exhibit humeral torsion greater than about 30° [36]. Torsion often increases with growth in temnospondyls and other early tetrapods, and in *H*. *longicostatum* and *M*. *pelikani*, in which adult torsion approaches 90°, levels around 30° are observed only in the least mature specimens.

A standard feature of vertebral development in *Greererpeton*, temnospondyls, and stem amniotes, is the development of the centrum from multipartite elements and the appearance of the neural arches well before the centrum [36,51,58]. Contrary to that pattern, the early and synchronous appearance of the arches and centra in *M*. *pelikani* and *H*. *longicostatum*, as well as the unipartite centrum, is characteristic of lepospondyls [58]. In earlier growth stages the neural arch halves may initially be present as sutured, paired elements, but the centra always are a single entity. Although previously *H*. *longicostatum* was reported to exhibit ossification of the centra prior to the arches [59], no evidence for that pattern exists because even in the smallest individuals known, arches and centra are already ossified [16].

Despite the discovery of extremely small individuals of some lepospondyls taxa (e.g., CGH3), only *Utaherpeton* provides any evidence of incomplete vertebrae in basal lepospondyls [13]. In that taxon, an immature specimen has trunk centra that may not have been completely ossified dorsally, as well as potentially paired pleurocentra in the posterior tail. Additionally, although that juvenile has a bifurcated ilium, which is observed only in relatively mature *M*. *pelikani* and *H*. *longicostatum* individuals, the pubis is unossified. Carroll and Chorn [13] suggested a morphogenetic sequence of a fimbriated to smooth interclavicle in *Utaherpeton*, which differs from the situation in *M*. *pelikani* in which even the largest individuals display fimbriation. That change in *Utaherpeton* also is unanticipated considering that the interclavicle is dermal in origin and dermal bone does not usually show major morphogenetic changes ([36]; this study).

### Inconsistent Morphogenesis

Results from earlier work on temnospondyls indicated that the level of postcranial morphogenesis is consistent across the skeleton [36]. In other words, in a given individual the stage of morphogenesis expressed by one element should match that of the other postcranial elements. If that statement is true, then the degree of osteological morphogenesis can be used as a size-independent criterion for inferring relative maturity in early tetrapods. In general, information on the skeletal morphogenesis of *H*. *longicostatum* and *M*. *pelikani* support that hypothesis. However, there were a few notable cases in *M*. *pelikani* in which variation for the level of morphogenesis was present.

One example is MB.Am.17; despite the clear ossification of a relatively large scapula, the radius and ulna of this individual exhibit a much earlier stage of development, whereby these bones are relatively featureless blocks. In another specimen (St.193), a relatively late-ossifying element, the pubis [16], is present, but the humerus displays minimal torsion and barely differentiated distal condyles. Inconsistency across the skeleton of a single specimen was reported for the long bones of other Paleozoic taxa as well, including basal synapsids [57].

Moreover, there is at least one case in *M*. *pelikani* in which the inconsistency is asymmetric, affecting the same element on different sides of the body. In that specimen (NHMW1983_32_66), the humerus on the right is robust, squat, and displays little waisting of the shaft. The humerus on the left, however, is more slender and elongate, and the proximal and distal ends of the element are distinct from the shaft. An example of this sort of asymmetry from outside my study comes from the analysis of *Utaherpeton* [13], in which the tibia and fibula on one side of the body are much less-developed than those from the other side of the body. Study of a juvenile of the Triassic thalattasaur *Anshunsaurus* also uncovered asymmetric levels of ossification in the contralateral ilia and scapulae [60].

### Postaxial and Preaxial Dominance in Tetrapod Limb Formation

The pattern of limb formation in extinct tetrapods is important for placing limb morphogenesis in extant tetrapods into proper phylogenetic context. Among living clades, frogs and amniotes share a pattern of limb chondrification in which the digits and distal epipodials form in a posterior to anterior direction (4–(5 or 3)–2–1), although digit 5 usually appears out of sequence (postaxial dominance; [61,62]). The posterior zeugopodium (ulna/fibula) forms slightly earlier than, or at the same time as, the anterior element. In addition, the metapodials and phalanges undergo chondrification and ossification in a generally proximal to distal order (note that the sequence of chondrification and ossification are not tightly coupled in all tetrapods [62]), with the exception of the distal-most phalanges, which may ossify prior to the proximal phalanges [63]. As in amniotes and frogs, the distal-most phalanges of salamanders also frequently ossify before the proximal phalanges [63]. However, salamanders are unique in possessing preaxial dominance; digits form in an anterior to posterior direction ((2–1)-3-4-5) and the radius/tibia precedes the ulna/fibula [61].

Only recently has evidence for preaxial dominance been found in Paleozoic tetrapods. Branchiosaurids ossify the digits in the order 2-3-1-4-5 and the anterior zeugopodium forms before the posterior [63]. Additionally, the amphibamid *Gerobatrachus* exhibits a distal tarsal ossification sequence that begins with a fused distal 1+2 (= basale commune, characteristic of salamanders) followed by distal 3 [4], although that pattern alone is not conclusive evidence of preaxial dominance.

The pattern observed in *H*. *longicostatum* and *M*. *pelikani* is different from those of branchiosaurids and *Gerobatrachus*. The first carpal or tarsal to ossify is always the intermedium. The distal epipodials form only after at least two out of the three proximal epipodials have ossified. In specimens that have an intermedium plus an additional ossified proximal epipodial in the foot, the second tarsal is the fibulare, which suggests a limb formation pattern similar to frogs and amniotes. That observation, coupled with morphogenetic changes in the posterior (ulnar/fibular) digits before the anterior digits, suggests postaxial dominance in *M*. *pelikani* and *H*. *longicostatum*. However, digit 5 is the final digit to ossify in the foot, and is substantially delayed relative to the other digits. Although digit 5 may also ossify last in salamanders and branchiosaurids, the large developmental delay between the ossification of metatarsal 5 in particular, and all other metatarsals and the phalanges of digits 1–4 strongly mirrors the pattern observed in extinct and extant non-synapsid amniotes [62]. As in many living frogs, amniotes, and salamanders, as well as the extinct branchiosaurid *Apateon* [62], the distal-most phalanges in *M*. *pelikani* and *H*. *longicostatum* display a mature morphology before the other phalanges and metapodials are completely ossified. It seems that *M*. *pelikani* and *H*. *longicostatum* had a generally plesiomorphic pattern of limb development, although the late (or weak) ossification of digit 5 suggests the presence of a shared developmental feature supporting the relationship between lepospondyls and amniotes, as well as more variation than what has been described previously for digit ossification in early tetrapods.

### Tail Growth and Regeneration

An investigation by Milner [15] conflicted with prior evidence for tail regeneration in *M*. *pelikani* that was presented and illustrated by Carroll and Gaskill ([1] reference figure 81F). The interpretation of Milner [15] was based on the fact that the sharp change in vertebral size represented in the figure was not observable in the specimen (NHMW1898-1043), and that in *M*. *pelikani* the tail lengthened during growth primarily by terminal addition of caudal vertebrae, which might be confused for regeneration. The specimen in question was unavailable to me, but I observed a similar pattern of anomalously small, neomorphic vertebrae in other specimens of *M*. *pelikani*. In the specimens that I examined, the transition from large, fully developed vertebrae to small, amorphous blocks is abrupt, suggesting that tail regeneration did occur. I also observed the same pattern in a number of specimens of *H*. *longicostatum*. However, the particular individual figured by Carroll and Gaskill ([1], reference figure 87B), in which there is no abrupt transition, does appear to support the comments made by Milner [15] regarding a gradual, prolonged terminal addition of caudal vertebrae. In living salamanders the two processes are not mutually exclusive [64] and thus it is plausible that both terminal addition and regeneration were present in some Paleozoic taxa.

Vertebral regeneration in *M*. *pelikani* and *H*. *longicostatum* resembles that of living salamanders rather than squamtes. In squamtes, the tail is regenerated as a solid cartilaginous rod. In salamanders, however, the tail reforms first as a cartilaginous rod which then segments into individual neomorphic neural arches and centra [64]. In regenerated salamander vertebrae, the centra form before the neural arches. That condition is similar to the pattern observed in the potentially regenerated tails of *M*. *pelikani* and *H*. *longicostatum* in which the posterior part of the regenerated tail exhibits both neomorphic arches and centra, but the anterior portion only has re-ossified centra. In arguing against the potential for tail regeneration in *M*. *pelikani*, Milner [15] stated that the relatively longer tails observed in some individuals are the result of gradual, continuous addition of tail vertebrae, and not of regeneration. That argument is partially supported by evidence from living salamanders in which regenerated tails are shorter than those that develop normally [64].

### Skeletal Growth and Developmental Mode

Relative to temnospondyls and stem tetrapods, ossification and growth in *M*. *pelikani* and *H*. *longicostatum* is relatively rapid and complete at small size ([16]; this study). Even the smallest individuals of *M*. *pelikani* and *H*. *longicostatum* have ossified metapodials, phalanges, long bones, vertebrae, and skulls. The timing and degree of ossification of the vertebrae particularly stand out when compared to the slowly ossifying and less well-developed vertebrae of temnospondyls. However, there was no condensation of development events, such as that described for branchiosaurid temnospondyls and extant amphibians, indicating a lack of metamorphosis in *M*. *pelikani* and *H*. *longicostatum*. Additional evidence for the absence of metamorphosis comes from the primarily isometric growth of the skeleton [17], which indicates lack of large-scale skeletal remodeling. The tempo and morphogenetic pattern of skeletal development in *M*. *pelikani* and *H*. *longicostatum* are more like those of early amniotes, which are considered to be direct developers that lacked a free-living larval stage during ontogeny [40].

However, there are a few features present in both *M*. *pelikani* and *H*. *longicostatum* that also do not cleanly fit the hypothesis of direct development. First, lateral lines are present in both taxa. In at least *M*. *pelikani*, the lateral lines are present even in the largest individuals, although they are reduced to only the frontal and jugal pits later in ontogeny. Secondly, branchial plates are found in both small and large individuals of *M*. *pelikani*, indicating that gill clefts at least were retained throughout ontogeny [1,3]. Although it is true that loss of the lateral lines is a hallmark for the transition to land in many temnospondyls and seymouriamorphs, and is included in the metamorphosis of branchiosaurids and extant amphibians, other aquatic temnospondyls and seymouriamorphs retain lateral lines as adults [26, 27,65,66,67]. In other words, lateral lines are not necessarily associated with metamorphosis or neoteny and are not a barrier to inferring a more amniote-like ontogeny. The branchial plates, however, indicate that individuals of *M*. *pelikani* possessed gill clefts for a large part of their lives. Associated external gills may have been present, but this cannot be stated conclusively; branchial plates also are retained in extant salamanders such as *Cryptobranchus* and *Amphiuma* that lose their external gills as adults, but retain gill clefts for suction feeding [68].

As noted earlier, the branchial plates are poorly preserved and are found only in a small number of individuals. The relationship between presence and absence is not related to size because even though only a small percentage of individuals exhibit evidence of the plates, the structures are observed in specimens at both ends of the known size range. However, potential evidence for loss of branchial structures in mature specimens may come from correlation with the level of skeletal morphogenesis. None of the specimens that display ossified carpals and tarsals also display branchial plates. In salamanders, temnospondyls, and seymouriamorphs, ossification of epipodials [36] and loss of gill structures [27,34,65,66] are regarded as a sign of skeletal maturity and a transition to a terrestrial lifestyle. Unfortunately, in the specimens of *M*. *pelikani* epipodials also are poorly preserved and are present only in a small number of relatively large individuals, which begs the question of whether that pattern is the result of chance. On the other hand, it is somewhat paradoxical that *M*. *pelikani*, a taxon historically considered to be neotenic, developed ossified carpals and tarsals, moderately well-ossified limbs and girdles with prominent processes and articulation surfaces, and a sutural, but otherwise well-ossified pelvic girdle including a distinctly ossified pubis and robust ilium. Those features are characteristic of mature, non-larval tetrapods and are not found in neotenic branchiosaurids or salamanders that retain gills and lateral lines [27,34].

Recent reviews of early tetrapod development that place the diversity of described patterns (particularly within Temnospondyli) in a comparative, phylogenetic context offer an alternative viewpoint on metamorphosis, neoteny, and the plesiomorphic developmental mode of tetrapods [26,28], and may facilitate the interpretation of the mosaic of developmental features in *M*. *pelikani* and *H*. *longicostatum*. Under new hypotheses, drastic, physical metamorphosis in the sense discussed by Alberch [21] is a derived feature of modern amphibians and specific clades of temnospondyls (e.g., dissorophoids), and therefore is not a primitive condition inherited from the most recent common ancestor of tetrapods [26,28,66]. However, the presence of a larva that gradually transforms into a juvenile and then into an adult (terrestrial or aquatic), in contrast to metamorphosing, is plesiomorphic for early tetrapods. That hypothesis is consistent with the details of the ontogeny of *M*. *pelikani* and *H*. *longicostatum*, and can explain the superficially conflicting evidence of lateral lines, branchial plates, and a direct-development-like, complete ossification at small size in *M*. *pelikani*.

Additionally, a new distinction was made between ‘perennebranchiate’ and neotenic lifestyles by Schoch [26], who redefined ‘perennebranchiate’ as a more general term describing the retention of external gills into adulthood. That definition is not extended to other features of anatomy, which independently could be paedomorphic, peramorphic, or unmodified (i.e., different parts of the organism could be subject to different processes). Neoteny, however, is specifically regarded as an alternative to a metamorphic mode of development whereby sexual maturity in a larval state is brought on specifically by suppressing metamorphosis in an otherwise metamorphic clade [26,27]. Suppression of metamorphosis leads to a delay in somatic development, and thus paedomorphic features.

Those views do not change many historical inferences about the environments that early tetrapods occupied, nor the morphogenetic steps that occurred during growth. What does change is the historical perspective that metamorphosis is an ancestral and anticipated mode of development. Instead, metamorphosis, a condensed period of whole-body reorganization, is derived, and neoteny specifically refers to the suppression of metamorphosis. However, even though the abandonment of metamorphosis is likely tied to underlying changes in the rate or onset/offset of development (e.g., deceleration, post-displacement), neoteny may not be as simple as pure ontogenetic truncation (*sensu* [29]). For example, in branchiosaurids considered to be neotenic, the largest specimens extend the larval developmental trajectory, resulting in the elaboration and hyper-development of features like larval tooth morphology and larval skull ornamentation [27,38]. Branchiosaurids that metamorphose, in contrast, transition from larval to adult temnospondyl morphologies, and those drastic skeletal changes occur in a condensed period of time relative to the ancestral temnospondyl pattern [27].

**Fig 38:**
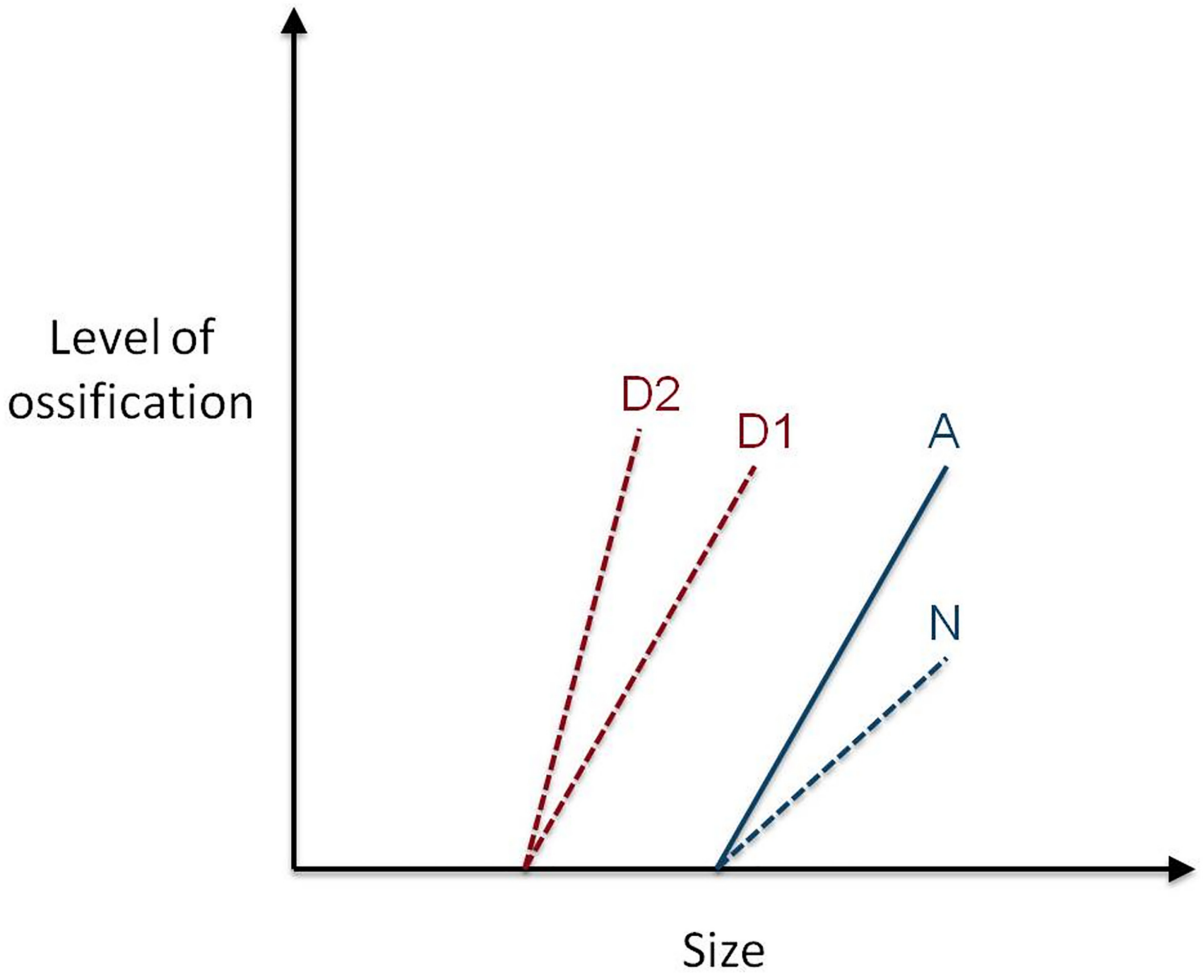
Heterochrony related to body size and level of skeletal ossification. Line leading to A represents ancestral condition. Line leading to N represents neoteny in the sense of a deceleration of rate of ossification, which produces a derived condition with a lesser degree of skeletal ossification. Line leading to D1 represents an earlier onset of ossification, producing a derived condition in which smaller body size is coupled with the same degree of ossification as ancestral condition. Line leading to D2 represents an acceleration in the rate of ossification combined with earlier onset, which produces a derived condition of smaller body size coupled with a higher degree of skeletal ossification.

In contrast to the pattern in branchiosaurids, the mosaic of developmental features in *M*. *pelikani* and *H*. *longicostatum* may actually point to peramorphosis of skeletal growth in the latter two taxa. If the ancestral tetrapod condition was to grow large while either remaining in the water or gradually completing a transition to land, then neoteny in the sense of a deceleration of somatic development [29,32] would lead to a derived condition with a lesser degree of skeletal ossification than the ancestral condition (Fig 38). In order to produce a derived condition in which smaller body size is coupled with a degree of skeletal ossification similar to the ancestral condition, at a minimum there must be an earlier onset of ossification (i.e., pre-displacement). An additional acceleration in the rate of ossification is necessary to produce a derived condition exhibiting both smaller body size and a higher degree of skeletal ossification, as in lepospondyls (Fig 38).

The difference between the ontogeny of amniotes and that of stem-tetrapods is a derived shift in the rate of skeletal growth and degree of ossification, or perhaps the timing of hatching or birth, whereby the juvenile amniote first contacts the environment at a later stage of development (i.e., more completely ossified) than the free-living larvae of stem-tetrapods. That extended development, in terms of the level of skeletal development at hatching or birth, and not necessarily the absolute time required for development, could have evolved along the amniote stem, prior to the evolution of the amniotic egg. In the seymouriamorph *Discosauriscus*, although the larval period is gradual and relatively long (plesiomorphic for tetrapods; [66]), histological analysis of long bone growth pointed to a more rapid rate of ossification than is present in salamanders [69].

The developmental patterns observed in *M*. *pelikani* and *H*. *longicostatum* are consistent with their stable placement as stem amniotes (this study; [4,6]), but at a position likely prior to the innovation of the amniotic egg. The rapid skeletal development and high degree of ossification at small size in *M*. *pelikani* and *H*. *longicostatum* matches that of basal amniotes and is probably a shared, derived condition. However, for many stem amniotes, such as *M*. *pelikani* and seymouriamorphs [66], development likely was still tied to water, implying that they did not have an amniotic egg. Because amniotes evolved from an anamniotic ancestor, it is inferred that stem lineages would still require water (or moisture) for reproduction [70]. However, other features of development, such as the rapid, complete ossification of the skeleton and the notable developmental delay of digit 5 of the foot, may have evolved earlier in the history of the amniote stem, leading to a mosaic of plesiomorphic and derived ontogenetic features in more distant amniote relatives, like lepospondyls.

In primarily aquatic lepospondyls, perhaps individuals hatched or were born with branchial structures initially but some taxa (i.e., *H*. *longicostatum*) lost them early in ontogeny, like living caecilians, whereas others remained in the water and retained aquatic features (i.e., *M*. *pelikani*). More terrestrial taxa may have lacked branchial structures and lateral lines entirely. Alternatively, *M*. *pelikani* reached a larger adult size than *H*. *longicostatum*, which means that it may have required a longer time to grow, which translates into more time in the water. Later in ontogeny, individuals of *M*. *pelikani* did develop ossified epipodials and pubes, tended to reduce the lateral line system, and potentially could have lost the branchial structures. It is possible that gradually, *M*. *pelikani* and *H*. *longicostatum* eventually transitioned to land or a more amphibious lifestyle. Comparatively few individuals of more terrestrial lepospondyl taxa are found in the Nýřany deposit; *Ricnodon*, *Crinodon*, and *Sparodus* together are known from fewer than ten specimens out of the hundreds or thousands of tetrapods collected from the locality. However, based on the paedomorphic condition of major long-bone processes (a common feature of aquatic early tetrapods; [36,71]), and previous evidence that both *M*. *pelikani* and *H*. *longicostatum* lacked major functional shifts during ontogeny [17], that hypothesis is unlikely. The limitations of the data cannot provide a clear answer at this point, but the evidence is compatible with the hypothesis that lepospondyls exhibited a mosaic pattern of development. That pattern included a plesiomorphic lack of metamorphosis, the absence of neoteny in the sense found in branchiosaurids and some extant salamanders, but also the presence of derived developmental features shared between lepospondyls and amniotes, such as complete ossification at small size. Moreover, it is possible that early tetrapods possessed a wider variety of, and perhaps more flexibility in, developmental modes than was appreciated previously. The variety and flexibility of developmental patterns present in extant amphibians alone [34], suggests that extrapolating a standard biphasic, metamorphic pattern to the development of all early tetrapods is erroneous and limits the questions and hypotheses that can be explored as new fossils and taxa are discovered in the future.

## Conclusions

Re-analysis of early tetrapod relationships using revised character scores are congruent with phylogenetic hypotheses that ‘microsaurs’ are paraphyletic with respect to other lepospondyls and stem amniotes. Considering the relatively stable phylogenetic position of *M*. *pelikani* and *H*. *longicostatum*, information about the ontogeny of these taxa has broader implications for the interpretation of ancestral and derived developmental features of early tetrapods. Both *M*. *pelikani* and *H*. *longicostatum* have lateral lines and the former also has ossified branchial elements, pointing to an aquatic lifestyle, and perhaps a reproductive mode still tied to water. That ancestral pattern also is seen in other stem amniotes, such as seymouriamorphs [66]. However, rapid and complete skeletal ossification at relatively small size in *M*. *pelikani* and *H*. *longicostatum* is shared only with amniotes. The combination of plesiomorphic and derived features observed in the two lepospondyls is congruent with the hypothesis that metamorphosis is a derived conditions found only in some modern amphibians (and possibly their close relatives), and that ancestrally tetrapods had an aquatic larva that gradually matured over an extended period of time [26]. However, an increase in the tempo of skeletal growth and the degree of ossification at hatching or birth appears to be a derived condition in amniotes and their relatives, which potentially evolved prior to the amniotic egg.

## Supporting Information

S1 Dataset (TNT)

S2 Dataset (TNT)

S1 Fig Strict consensus tree resulting from the modification of character scores for *Microbrachis* and *Hyloplesion* in the matrix of Ruta and Coates [6]. (TIF)

S2 Fig Strict consensus tree resulting from the original matrix of Huttenlocker et al. [5]. (TIF)

S3 Fig Strict consensus tree resulting from the modification of character scores for *Microbrachis* and the addition of *Hyloplesion* to the matrix of Huttenlocker et al. [5]. (TIF)

S1 File Modified matrix of Ruta and Coates (2007). Formatted for TNT.
(ZIP)

S2 File Modified matrix of Huttenlocker et al. [5]. Formatted for TNT.
(ZIP)

S1 Table All sampled specimens of *M*. *pelikani*. Arranged by maturity of skeleton, based on number of ossified elements (see [16]). Abbreviations: sl, skull length; tl, trunk length.
(DOCX)

S2 Table All sampled specimens of *H*. *longicostatum*. Arranged by maturity of skeleton, based on number of ossified elements (see [16]). Abbreviations: sl, skull length; tl, trunk length.
(DOCX)

S3 Table Summary of character score modifications to the matrix of Ruta and Coates [6]. Result from anatomical re-description of *M*. *pelikani* and *H*. *longicostatum* and identification of ontogenetic variation in those taxa.
(DOC)

S4 Table Summary of character score modifications to the matrix of Huttenlocker et al. [5]. Based directly on Anderson et al. [4]; result from anatomical re-description of *M*. *pelikani* and identification of ontogenetic variation in that taxon.
(DOC)
