# Supplementary figures and images for: Skeletal Morphogenesis of Microbrachis and Hyloplesion (Tetrapoda: Lepospondyli), and Implications for the Developmental Patterns of Extinct, Early Tetrapods
Source: PLoS One. 2015 Jun 17;10(6):e0128333. doi: 10.1371/journal.pone.0128333 (PMC4470922; doi:10.1371/journal.pone.0128333)

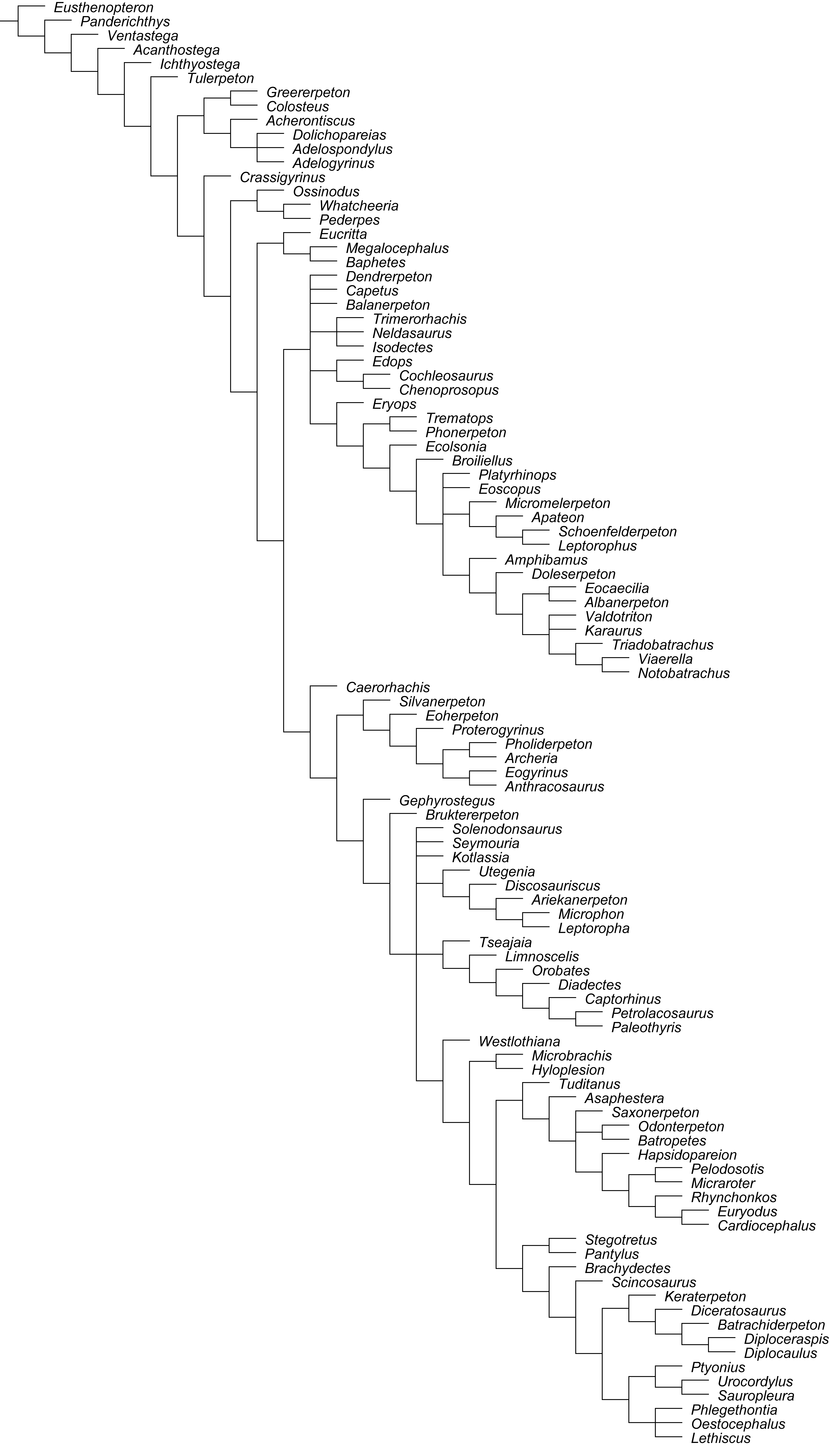

Supplement: S1 Fig — (TIF) [file pone.0128333.s003.tif]

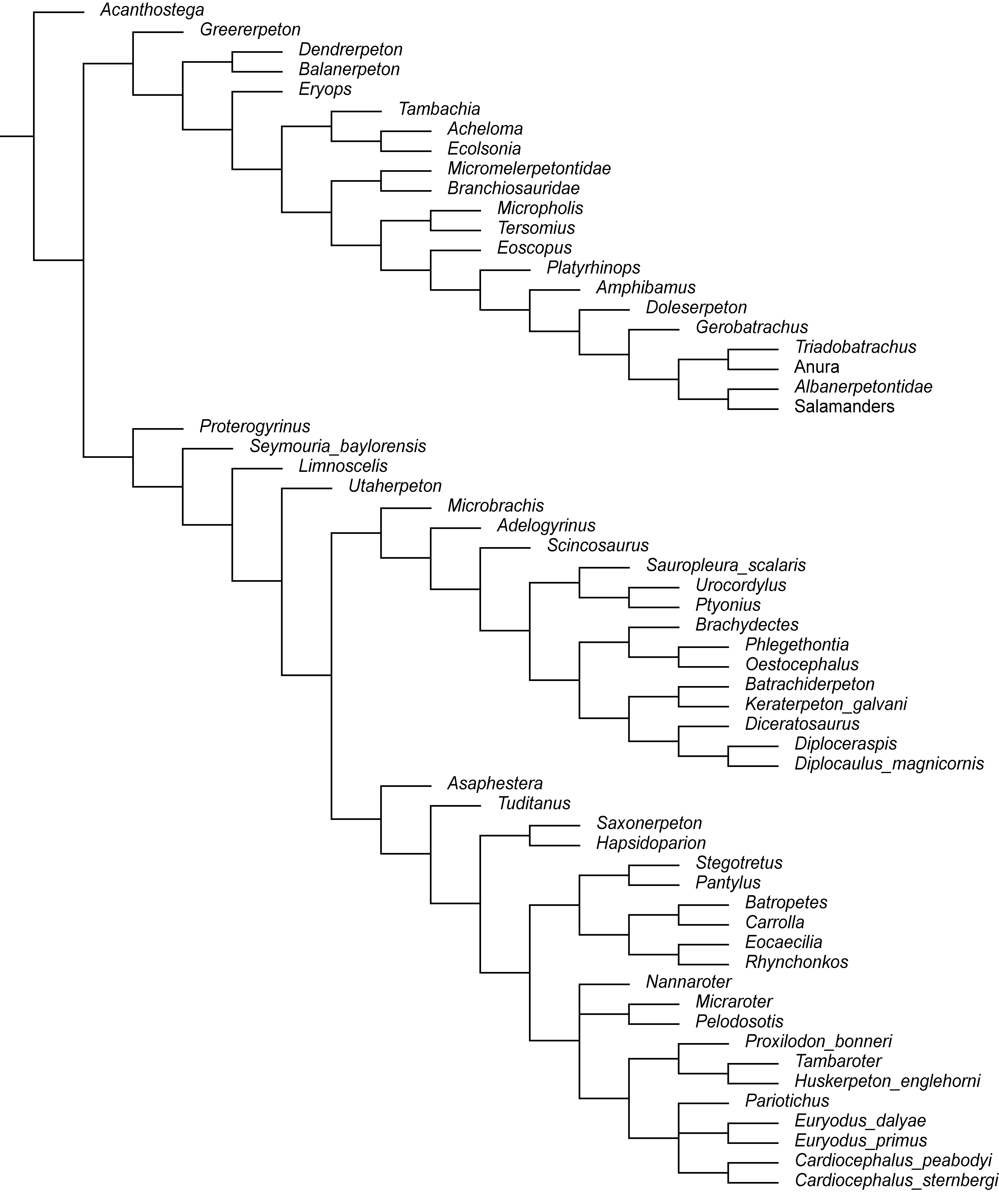

Supplement: S2 Fig — (TIF) [file pone.0128333.s004.tif]

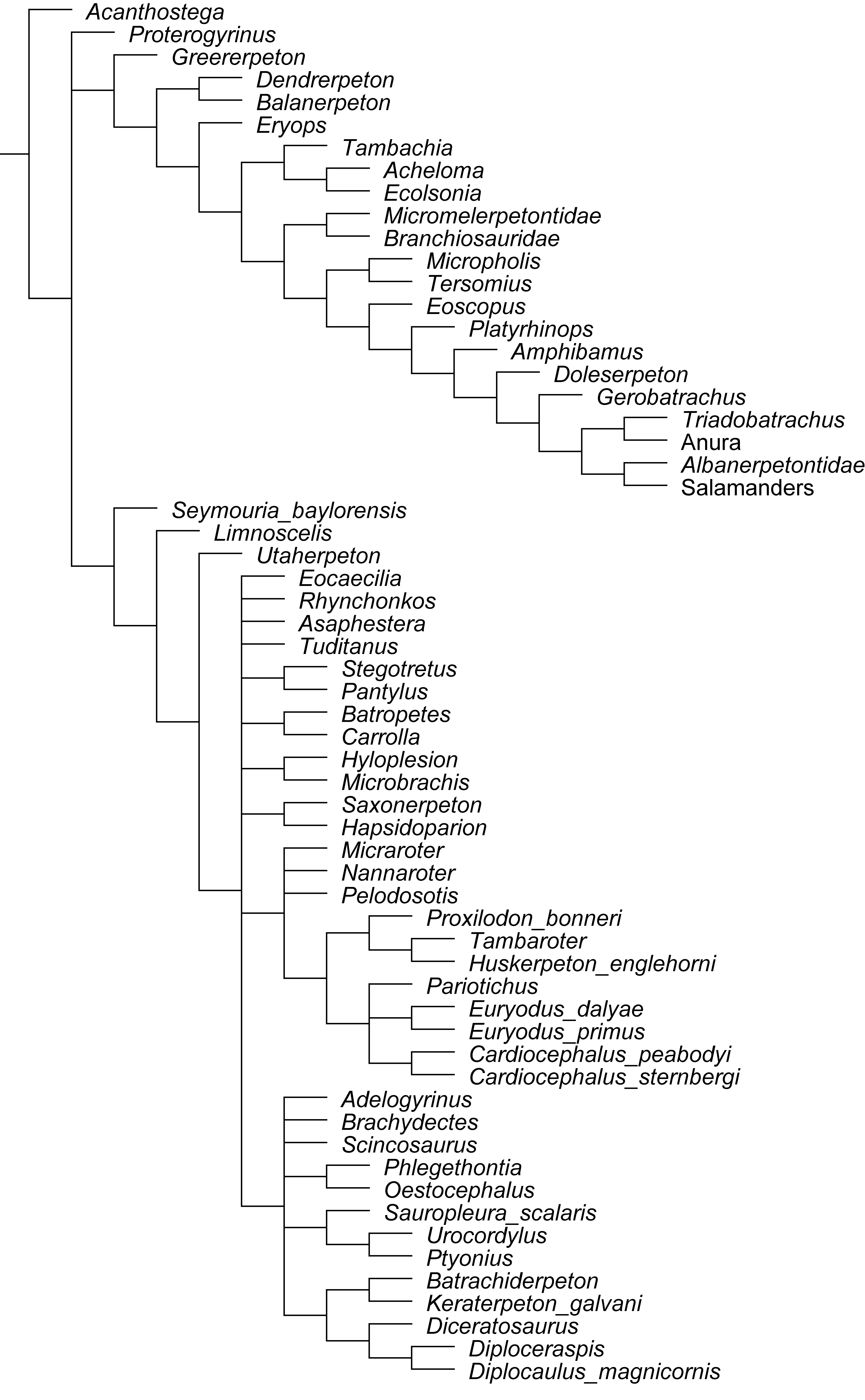

Supplement: S3 Fig — (TIF) [file pone.0128333.s005.tif]
