# Supplementary material for: Skeletal Morphogenesis of Microbrachis and Hyloplesion (Tetrapoda: Lepospondyli), and Implications for the Developmental Patterns of Extinct, Early Tetrapods
Source: PLoS One. 2015 Jun 17;10(6):e0128333. doi: 10.1371/journal.pone.0128333 (PMC4470922; doi:10.1371/journal.pone.0128333)
Supplement: S1 Table — Arranged by maturity of skeleton, based on number of ossified elements (see [16]). Abbreviations: sl, skull length; tl, trunk length. (DOCX) [file pone.0128333.s008.docx]

**S1 Table.** **All sampled specimens of *M. pelikani*.** Arranged by maturity of skeleton, based on number of ossified elements (see Olori, 2013a). Abbreviations: sl, skull length; tl, trunk length.

| **specimen** | **sl** | **tl** |
| --- | --- | --- |
| **CGH267** | 8.2 | . |
| **M1686** | 16.5 | . |
| **St.190** | 9.4 | 46 |
| **NHMW1983_32_66** | 7 | . |
| **MB.Am.809** | . | 45 |
| **NHMW1894_2400** | . | 46 |
| **NHMW1983_32_59** | . | 47+ |
| **NHMW1898_x_29** | 10 | 54 |
| **St.203** | 11+ | . |
| **MB.Am.813** | 13 | 64 |
| **NHMW1983_32_50&52** | 13 | 58? |
| **NHMW1898_X_33** | 13 | 68? |
| **St.204** | 13.2 | 53 |
| **MB.Am.821.1-2** | 14 | 65 |
| **M1381** | 14 | 65 |
| **NHMW1899_III_8** | 14.5 | . |
| **MB.Am.827** | 14+ | . |
| **St.199** | 15 | 67 |
| **St.208** | 15 | . |
| **MB.Am.833** | 15 | 64 |
| **NHMW1898_X_30** | 15 | 70? |
| **MB.Am.815.1-6** | 15 | 88 |
| **M1700** | 15.5 | 71 |
| **NHMW1983_32_64&74** | 15.7 | . |
| **M1681** | 15+ | 80 |
| **CGH69** | . | . |
| **CGH139** | 15+ | 71 |
| **MB.Am.811** | 16 | 76+ |
| **St.193** | 11 | 52 |
| **NHMW1983_32_72** | 17 | . |
| **MB.Am.825.1-3** | 17 | . |
| **M384** | 17.5 | . |
| **MB.Am.814** | 18 | . |
| **M1693** | 19 | . |
| **M 4884** | 19 | . |
| **NHMW1894_2332** | 19 | 77.5 |
| **NHMW1983_32_80ab** | 20 | 71 |
| **M3321-3322** | 20.1 | . |
| **CGH251** | 26 | . |
| **MB.Am.812.1-2** | 20? | 75? |
| **NHMW1894-2399** | . | . |

| **specimen** | **sl** | **tl** |
| --- | --- | --- |
| **MB.Am.840.1-2** | . | . |
| **MB.Am.74** | . | . |
| **MB.Am.828** | . | . |
| **M1694** | 17+ | 79 |
| **M4883** | 17 | 74 |
| **MB.Am.823** | 17 | 71+ |
| **St.201** | 18 | . |
| **NHMW1983_32_76** | 19 | 87? |
| **NHMW1983_32_49ab** | 19? | 79.5 |
| **CGH34** | 19.5 | . |
| **CGH138** | 20.5 | 90+ |
| **NHMW1896_X_36** | 20.5 | 101? |
| **NHMW1894-2364** | 22 | . |
| **NHMW1899_III_7** | 23 | . |
| **M1688=MB.Am.834** | 25 | . |
| **MB.Am.830.1-3** | 26 | . |
| **St.207** | 27 | 110+ |
| **CGH5** | . | 78? |
| **CGH2098** | 22 | 82 |
| **St.198** | 19 | . |
| **M639** | 16 | 92 |
| **MB.Am.810.1-2** | 17 | 80 |
| **Amnh2557** | 17.5 | 69 |
| **NHMW1983_32_67** | 27.5 | . |
| **CGH142** | . | . |
| **CGH727** | 17 | . |
| **CGH254** | 20? | 73 |
| **M4886** | 26 | 115 |
| **MB.Am.822.1=MB.Am.17** | 28 | . |
| **CGH3018** | 28 | . |
| **MB.Am.838.1-3** | 29 | . |
| **St.116** | 20+ | 120 |
| **M1683** | . | . |
| **CGH256** | . | . |
| **M1689** | . | . |
| **NHMW1983_32_3** | . | . |
