# Supplementary material for: Skeletal Morphogenesis of Microbrachis and Hyloplesion (Tetrapoda: Lepospondyli), and Implications for the Developmental Patterns of Extinct, Early Tetrapods
Source: PLoS One. 2015 Jun 17;10(6):e0128333. doi: 10.1371/journal.pone.0128333 (PMC4470922; doi:10.1371/journal.pone.0128333)
Supplement: S2 Table — Arranged by maturity of skeleton, based on number of ossified elements (see [16]). Abbreviations: sl, skull length; tl, trunk length. (DOCX) [file pone.0128333.s009.docx]

**S2 Table.** **All sampled specimens of *H. longicostatum.*** Arranged by maturity of skeleton, based on number of ossified elements (see Olori, 2013a). Abbreviations: sl, skull length; tl, trunk length.

| **specimen** | **sl** | **tl** |
| --- | --- | --- |
| **CGH3** | 3.9 | 13 |
| **NHMW1983/32/54** | 6.5 | 28 |
| **NHMW1899_IX_8** | 8 | 26 |
| **St.152** | 8 | 31 |
| **CGH3028** | 8.9 | 35 |
| **M4885a,b** | 9 |  |
| **CGH45** | . | . |
| **CGH15** | . | . |
| **M1377** | 12 | 45 |
| **NHMW1898_x_23** | 9.3 | 31.5 |
| **St. 209** | 19 | 45 |
| **RSM1899.32.3** | 17.2 | 58 |
