# Supplementary material for: Skeletal Morphogenesis of Microbrachis and Hyloplesion (Tetrapoda: Lepospondyli), and Implications for the Developmental Patterns of Extinct, Early Tetrapods
Source: PLoS One. 2015 Jun 17;10(6):e0128333. doi: 10.1371/journal.pone.0128333 (PMC4470922; doi:10.1371/journal.pone.0128333)
Supplement: S3 Table — Result from anatomical re-description of M. pelikani and H. longicostatum and identification of ontogenetic variation in those taxa. (DOC) [file pone.0128333.s010.doc]

**S3 Table. Summary of character score modifications to the matrix of Ruta and Coates (2007).** Result from anatomical re-description of *M. pelikani* and *H. longicostatum* and identification of ontogenetic variation in those taxa.

| **Character Number** | **Character** | **Taxon** | **Original Score** | **Modified Score** | **Redundant Characters** |
| --- | --- | --- | --- | --- | --- |
| 120 | Lateral line system on skull table *fully* enclosed (0), mostly enclosed, short sections in grooves (1), mostly in grooves, short sections enclosed  (2), all in grooves (3), absent (4) | *Hyloplesion*  *Microbrachis* | 4  2,3 | 3  3 | 46 of Anderson (2007); 64 of Anderson et al., 2008 |
| 121 | Mandibular canal (of lateral line system) fully enclosed (0), mostly  enclosed, short sections in grooves (1), mostly in grooves, short sections enclosed (2), all in grooves (3), absent (4). | *Hyloplesion* | 0,1 | 3 |  |
| 123 | Vomer with (0) or without (1) fangs equal to or larger than marginal teeth | *Hyloplesion* | ? | 1 | 10 of Zanon (1985; see also 12); 90 of Anderson (2007); 116 of Anderson et al. (2008) |
| 125 | Vomer does not (0) or does contribute to (1) interpterygoid vacuities | *Hyloplesion* | 1 | 0 | 10 of Zanon (1985; see also 12); 90 of Anderson (2007); 116 of Anderson et al. (2008) |
| 150 | Pterygoids sutured with each other: present (0); absent (1) | *Hyloplesion*  *Microbrachis* | 0  ? | 1  1 |  |
| 176 | Elongate, strut-like cultriform processof Parasphenoid: absent (0) or present (1) | *Hyloplesion* | 0 | 1 |  |
| 241 | Interclavicle wider than long: absent (0); present (1) | *Hyloplesion* | ? | 1 |  |
| 253 | Entepicondyle foramen is present (0) or absent (1) | *Hyloplesion* | ? | 0 |  |
| 255 | ectepicondyle ridge is present (0) or absent (1) | *Microbrachis* | ? | 1 | 160 of Anderson (2007); 199 of Anderson et al. (2008); 7 of Zanon (1985) |
| 258 | Waisted shaft of humerus: absent (0); present (1) | *Microbrachis* | 0 | 1 |  |
| 259 | Radial condyle position is terminal (0) or ventral (1) | *Hyloplesion* | ? | 1 |  |
| 269 | Radius longer than (0) equal to (1), or  shorter than (2) ulna | *Hyloplesion* | 2 | 1 |  |
| 271 | Olecranon process of ulna is absent (0) or present (1) | *Microbrachis* | 0 | 1 | 166 of Anderson, (2007); 205 of Anderson et al. (2008) |
| 272 | Dorsal iliac process is absent (0) or present (1) | *Hyloplesion* | 0 | 1 | 170 of Anderson, (2007); 211 of Anderson et al. (2008) |
| 291 | Proximal tarsal ossifications: absent (0); present, single (1); present, more than two(2) | *Microbrachis* | ? | 2 |  |
| 292 | Proximal tarsal element is not (0), or is (1) L-shaped | *Microbrachis* | ? | 0 |  |
| 303 | Axis neural arch not fused (0) or fused (1) to axis centrum | *Hyloplesion* | ? | 1 | 117 of Anderson, (2007); 149 of Anderson et al. (2008) |
| 307 | Neural and haemal spines aligned dorsoventrally: no (0); yes (1). | *Hyloplesion* | ? | 0 |  |
| 308 | Haemal spines not fused (0) or fused  (1) to caudal centra | *Hyloplesion* | ? | 0 | 124 of Anderson, (2007); 157 of Anderson et al. (2008) |
| 309 | Accessory articulations on haemal spines: absent (0); present (1). | *Hyloplesion* | ? | 0 |  |
| 310 | Ossified pleurocentra is absent (0) or  present (1) | *Hyloplesion* | ? | 0 |  |
| 311 | Trunk pleurocentra are not (0) or are (1) fused midventrally | *Hyloplesion* | ? | 1 |  |
| 312 | Trunk pleurocentra are not (0) or are (1) fused middorsally | *Hyloplesion* | ? | 1 |  |
| 314 | Neural arches of trunk vertebrae not (0) or are (1) fused to centra | *Hyloplesion*  *Microbrachis* | ?  0 | 0/1  0/1 | 117 of Anderson, (2007); 149 of Anderson et al. (2008) |
| 316 | Trunk intercentra are present (0) or absent (1) | *Hyloplesion* | ? | 1 |  |
| 319 | haemal spines of tail vertebrae without (0) or with (1) strong proximal emargination along  anterior and posterior margins | *Hyloplesion* | ? | 0 |  |
| 320 | vertebral centra without (0) or with (1) striated ornamentation | *Hyloplesion* | ? | 0 |  |
