# Supplementary material for: Skeletal Morphogenesis of Microbrachis and Hyloplesion (Tetrapoda: Lepospondyli), and Implications for the Developmental Patterns of Extinct, Early Tetrapods
Source: PLoS One. 2015 Jun 17;10(6):e0128333. doi: 10.1371/journal.pone.0128333 (PMC4470922; doi:10.1371/journal.pone.0128333)
Supplement: S4 Table — Based directly on Anderson et al. [4]; result from anatomical re-description of M. pelikani and identification of ontogenetic variation in that taxon. (DOC) [file pone.0128333.s011.doc]

**S4 Table. Summary of character score modifications to the matrix of Huttenlocker et al. (2013).** Based directly on Anderson et al. (2008); result from anatomical re-description of *M. pelikani* and identification of ontogenetic variation in that taxon.

| **Character Number** | **Character Description** | **Taxon** | **Original Score** | **Modified Score** | **Redundant Characters** |
| --- | --- | --- | --- | --- | --- |
| 14 | Participation of lacrimal in naris: present (0); absent (1) | *Microbrachis* | 1 | 0 | 10 of Anderson (2007) |
| 51 | Contact between the parietal and squamosal: absent (0); present (1) | *Microbrachis* | 1 | 0 | 35 of Anderson (2007) |
| 116 | Pterygoids contact one another anteriorly: present (0); absent (1) | *Microbrachis* | 1 | 0 |  |
| 121 | * no change, but note that states were revised from Anderson (2008) | *Microbrachis* | 0 | 0 |  |
| 141 | Ossified hyoids: present (0); absent (1) | *Microbrachis* | 0 | ? | 111 of Anderson (2007) |
| 149 | Trunk neural arch: loosely articulated (0); sutured (1); or fused (2) to centrum | *Microbrachis* | 1 | 1/2 | 117 of Anderson, (2007); 303 and 314 of Ruta and Coates (2007) |
| 163 | Trunk arches: paired (0); or fused (1) | *Microbrachis* | 1 | 0/1 |  |
| 198 | Ossification of scapulocoracoid: both elements (0); scapula only (1); absent (2) | *Microbrachis* | 1 | 0 | 159 of Anderson (2007) |
| 201 | Deltapectoral crest: weak (0); intermediate (1); prominent (2) | *Microbrachis* | 0 | 1 | 162 of Anderson (2007) |
| 205 | Olecranon process: unossified (0); ossified (1) | *Microbrachis* | 0 | 1 | 166 of Anderson (2007); 271 of Ruta and Coates (2007) |
| 221 | Area of sella turcica weakly ossified and sometimes open ventrally in parasphenoid: (0) absent; (1) present | *Microbrachis* | 0 | ? |  |
| 222 | Dorsal sinus between synotic tectum and parietals: (0) absent; (1) present | *Microbrachis* | 0 | ? |  |
| 223 | Frontal ventral process connection to sphenethmoid: (0) absent; (1) mediated via orbitosphenoid; (2) direct connection to sphenethmoid | *Microbrachis* | - | ? |  |
